# Phylogenetic and Evolutionary Patterns in Microbial Carotenoid Biosynthesis Are Revealed by Comparative Genomics

**DOI:** 10.1371/journal.pone.0011257

**Published:** 2010-06-22

**Authors:** Jonathan L. Klassen

**Affiliations:** Department of Biological Sciences, University of Alberta, Edmonton, Alberta, Canada; Universidad Miguel Hernandez, Spain

## Abstract

**Background:**

Carotenoids are multifunctional, taxonomically widespread and biotechnologically important pigments. Their biosynthesis serves as a model system for understanding the evolution of secondary metabolism. Microbial carotenoid diversity and evolution has hitherto been analyzed primarily from structural and biosynthetic perspectives, with the few phylogenetic analyses of microbial carotenoid biosynthetic proteins using either used limited datasets or lacking methodological rigor. Given the recent accumulation of microbial genome sequences, a reappraisal of microbial carotenoid biosynthetic diversity and evolution from the perspective of comparative genomics is warranted to validate and complement models of microbial carotenoid diversity and evolution based upon structural and biosynthetic data.

**Methodology/Principal Findings:**

Comparative genomics were used to identify and analyze *in silico* microbial carotenoid biosynthetic pathways. Four major phylogenetic lineages of carotenoid biosynthesis are suggested composed of: (i) Proteobacteria; (ii) Firmicutes; (iii) Chlorobi, Cyanobacteria and photosynthetic eukaryotes; and (iv) Archaea, Bacteroidetes and two separate sub-lineages of Actinobacteria. Using this phylogenetic framework, specific evolutionary mechanisms are proposed for carotenoid desaturase CrtI-family enzymes and carotenoid cyclases. Several phylogenetic lineage-specific evolutionary mechanisms are also suggested, including: (i) horizontal gene transfer; (ii) gene acquisition followed by differential gene loss; (iii) co-evolution with other biochemical structures such as proteorhodopsins; and (iv) positive selection.

**Conclusions/Significance:**

Comparative genomics analyses of microbial carotenoid biosynthetic proteins indicate a much greater taxonomic diversity then that identified based on structural and biosynthetic data, and divides microbial carotenoid biosynthesis into several, well-supported phylogenetic lineages not evident previously. This phylogenetic framework is applicable to understanding the evolution of specific carotenoid biosynthetic proteins or the unique characteristics of carotenoid biosynthetic evolution in a specific phylogenetic lineage. Together, these analyses suggest a “bramble” model for microbial carotenoid biosynthesis whereby later biosynthetic steps exhibit greater evolutionary plasticity and reticulation compared to those closer to the biosynthetic “root”. Structural diversification may be constrained (“trimmed”) where selection is strong, but less so where selection is weaker. These analyses also highlight likely productive avenues for future research and bioprospecting by identifying both gaps in current knowledge and taxa which may particularly facilitate carotenoid diversification.

## Introduction

Carotenoids comprise a large secondary metabolite family of over 600 isoprenoid compounds and are produced by most plants and many microorganisms [1]. Depending on the length of their conjugated double bond chain and the nature of its substituents, carotenoids most often absorb light in the 300–600 nm range to appear yellow, orange or red [2]. Carotenoids are structurally divided into two classes: carotenes, which are exclusively hydrocarbons, and xanthophylls, which are oxygenated [2].

Carotenoid function is perhaps best understood in photosynthetic light-harvesting complexes, where carotenoids dissipate excess energy and radicals from excited oxygen and (bacterio)chlorophyll molecules, physically structure the photosynthetic reaction center and act as accessory light-harvesting pigments [3]–[5]. In all organisms carotenoids may function as antioxidants and promote oxidative stress resistance (e.g., [6], [7]), and even act as a virulence factor in *Staphylococcus aureus* by promoting resistance to neutrophil oxidative burst [8]. Membrane fluidity and proton permeability may also be modulated by carotenoids in all organisms, depending on carotenoid structure and concentration [9], [10]; these latter functions remain poorly studied, especially *in vivo*. Carotenoids can also be cleaved to form apocarotenoids. These include retinal (Vitamin A), the cofactor of the photoactive rhodopsin protein found in many microorganisms [11], [12] and functionally similar light-sensing proteins in vertebrates [13]. At least one rhodopsin (xanthorhodopsin) also interacts directly with antennae carotenoids [14]. Other apocarotenoids include plant hormones, fungal pheromones and antifungal compounds [15].

Carotenoids are biotechnologically high-value compounds with an annual market estimated to exceed one billion US dollars by 2010 (cited in [16]). Applications include natural pigments [17] and nutraceuticals based on the potential of carotenoids to decrease the risk of several human diseases [18]–[20]. This biotechnological interest has prompted extensive research into both natural [16] and recombinant carotenoid production, particularly in microbes [21]. As part of the latter approach, carotenoids are a model system [22] to study recombinant biosynthetic pathway engineering [23]–[25], by which novel compounds are produced by combining genes from multiple organisms in a heterologous host. This approach has resulted in novel carotenoids with enhanced biotechnologically relevant properties such as antioxidative strength [26], [27]. Despite underlying pathway engineering initiatives, however, microbial carotenoid biosynthetic and structural diversity and distribution have been significantly underestimated due to utilization of methods lacking either taxonomic breadth or structural resolution [28].

Carotenoid diversity has been hitherto described from structural [1] and biosynthetic perspectives [29]–[33]. Whereas evolutionary models based upon chemical data are weakened by the lack of phylogenetic signal that these data contain, the genes and proteins coding for their cognate biosynthetic functions are well-studied, character-rich and evolve in concert with their biosynthetic products. Their sequences are therefore ideal for determining the evolution of carotenoid biosynthesis, and by extension, carotenoid structural diversity. Unfortunately, except for photosynthetic microbes [31], [33], syntheses of carotenoid biosynthesis have focused exclusively (or nearly so) on proteins with biochemically- or genetically-demonstrated functions to the neglect of their homologs in other organisms (e.g., [34], [35]). The degree to which these relatively few studied taxa represent the vast majority of microbial life may therefore be questioned. Furthermore, whereas some studies demarcate phylogenetic lineages of microbial carotenoid evolution, they do so without proper consideration of the bootstrap support for their presented phylogenies [34], [35] and in one case misidentified *Paracoccus zeaxanthinifaciens* as *Flavobacterium* sp. ATCC 21588, the only member of the Bacteroidetes included [35]. Now that several hundred genome sequences are available, a re-evaluation of these data using robust phylogenetic and evolutionary methods is clearly warranted.

The objectives of the present research are three-fold. First, the overall phylogenetic structure of carotenoid biosynthesis is determined by considering the phylogenetic distribution of microbial carotenoid structural diversity and how it relates to phylogenies of core carotenoid biosynthetic proteins. These analyses allow inference of significant patterns and events in microbial carotenoid evolution. Secondly, this phylogenetic structure is used to re-evaluate the evolution of two major carotenoid biosynthetic protein families: carotenoid desaturase CrtI-family enzymes and carotenoid cyclases. Whereas the evolution of these protein families have been discussed previously [34], [36], [37], this has been primarily from the perspective of biochemistry and not phylogeny. Finally, these data are used to ask both whether the evolutionary mechanisms acting on microbial carotenoid biosynthesis are equivalent in all taxa, and to what extent this process might accurately be arrayed as “tree-like” as conjectured previously [22], [28], whereby conserved core enzymes form the “root” and more terminal “branches” diverge from it. These patterns are also used to suggest likely avenues for productive future research and bioprospecting.

## Methods

### Dataset Construction

Carotenoid biosynthetic enzymes with known function were identified from the literature (see Table S1) and their corresponding amino acid sequences retrieved from GenBank (http://www.ncbi.nlm.nih.gov/). Enzymes were considered of demonstrated biosynthetic function if (by order of confidence): (i) they had been confirmed by *in vitro* biochemical studies; (ii) their recombinant expression in a non-carotenogenic host resulted in an appropriate anabolic reaction; or (iii) *in vivo* mutation of their cognate gene resulted in a loss of function. In the later case, functional assignments were subsequently confirmed by homology of these sequences with relatives of known function due to the possibility of polar mutations eliciting misleading phenotypes. In a few cases, amino acid sequences for proteins of confirmed function were unidentifiable due to missing GenBank accession numbers or genomic gene identifiers in the literature; these sequences were omitted from the initial seed database because alternative close homologs were available.

**Figure 1:**
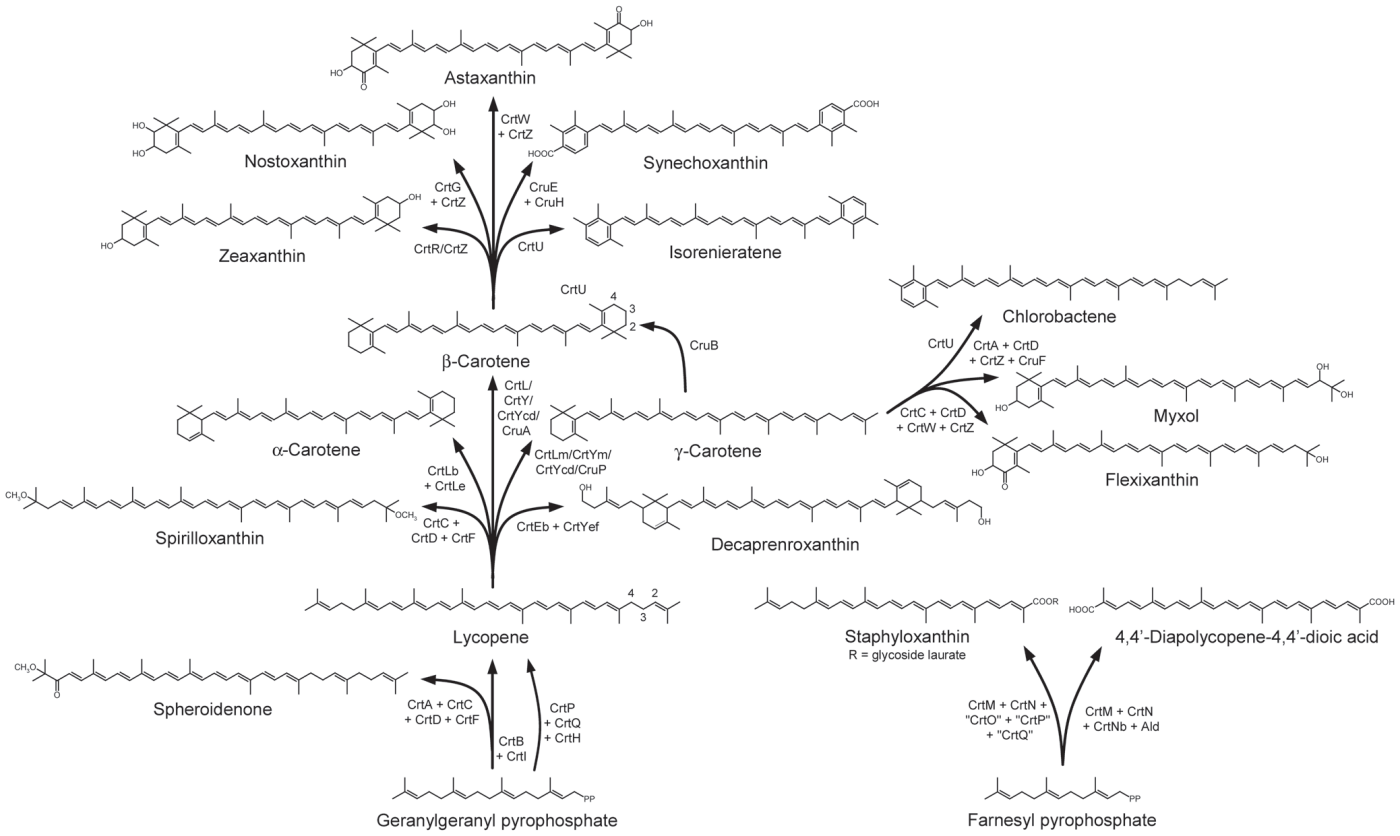
Known carotenoid biosynthetic pathways. For simplicity, only representative carotenoids and major intermediates are shown. Functionally equivalent enzymes are indicated by a slash; for alternative names of homologous sequences see Table S3. Carbon numbers are indicated for lycopene and β-carotene.

Non-bootstrapped phylogenetic trees for each protein type in the initial seed database were constructed and representatives from each obtained phylogenetic cluster were used to iteratively search the Integrated Microbial Genome (IMG) database version 2.4 [38], last updated December 2007, using BLASTp [39]. For each protein type, all BLAST hits with an expectation value <1×10^−20^ were exported along with their corresponding nucleotide sequences. To eliminate obviously spurious and paralogous sequences, non-bootstrapped phylogenetic trees were constructed to determine to which, if any, carotenoid biosynthetic enzyme family the recovered sequences belonged. Sequences were annotated based primarily upon phylogenetic clustering with those of demonstrated functions from the initial seed database, either in obvious clades or adjacent to them in accordance with the taxonomy of their originating organisms. Sequences were also annotated based upon the construction of logical carotenoid biosynthetic pathways, according to both currently described carotenoid biosynthetic pathways and known chemical structures (Figure 1, Table S1). In all cases sequence assignments were made conservatively, i.e. sequences were removed if there was no clear reason for their inclusion, favoring a lower rate of false-positive assignment at the expense of a higher false-negative assignment rate.

Because the IMG database is updated only intermittently, representative sequences for each protein type retrieved from the IMG database were used as inputs for PSI-BLAST [40] searches against the GenBank reference protein sequence database. Non-genome derived sequences present in the GenBank non-redundant database were excluded because their organismal identities typically lacked corroborating evidence. Three PSI-BLAST iterations were conducted with an expectation value threshold set such that all previously identified sequences were recovered. Sequences obtained by this approach were compared to those from the IMG and initial seed databases using non-bootstrapped phylogenetic trees, and sequences unique to the GenBank database and that clustered internal to previously recovered IMG and seed sequences were retained. In cases where a particular sequence was absent from a biosynthetic pathway inferred from the sorted sequence database, the corresponding genome was specifically queried for that homolog using BLAST. Where multiple closely related strains (i.e. nearly 100% protein sequence identity for all protein types) were recovered, only one sequence was retained as a representative (Table S1). Whereas in most cases seed sequences (i.e. those recovered from the literature) were used in preference to genomic data, occasionally a genome-sequenced strain was chosen as the representative due to the greater number of putative carotenoid biosynthesis enzyme sequences present (Table S1). Because the IMG and GenBank databases contained few algal genomes, the genome database sites for *Cyanidioschyzon merolae* (http://merolae.biol.s.u-tokyo.ac.jp/), *Galdieria sulphuraria* (http://genomics.msu.edu/galdieria/), *Phaeodactylum tricornutum* (http://genome.jgi-psf.org/Phatr2/Phatr2.home.html/) and *Thalassiosira pseudonana* (http://genomeportal.jgi-psf.org/Thaps3/Thaps3.home.html) were individually searched with previously identified algal and cyanobacterial sequences using BLAST.

In addition to whole-genome sequence data, carotenoid biosynthetic protein sequences from uncultured organisms represented by large-insert fosmid clones from oceanic surface waters of Monterey Bay and the North Pacific Subtropical Gyre [41] were included to better represent natural proteorhodopsin diversity. Only fosmid clones containing a putative full carotenoid biosynthetic pathway leading to rhodopsin and a clear phylogenetic identity were included in the dataset to best facilitate pathway reconstruction. The presence of rhodopsin genes in the analyzed genome sequences was determined by searching the GenBank refseq database using three sequential PSI-BLAST iterations with a 1×10^−5^ expectation value cut-off. Searches were conducted using rhodopsins from *Halobacterium salinarium*, *Nostoc* sp. PCC 7120 and *Pelagibacter ubique* HTCC1062 (GenBank accession numbers 0501217A, NP_487205 and AAZ21446, respectively) as seed sequences, and recovered all proteorhodopsin sequences annotated previously [12], [42]. Sequences below this threshold were compared phylogenetically without bootstrapping to exclude sequences outlying those with previously demonstrated function, those from the included metagenomic study [41] or organisms lacking appropriate carotenoid biosynthetic enzyme homologs.

To include carotenoid biosynthetic sequences from *Candidatus* “Chloracidobacterium thermophilum”, the fosmid-cloned sequences reported by Bryant *et al*. [43] were BLAST-searched using known carotenoid biosynthetic protein sequences. Whereas the CrtH and CrtP proteins described in this study were recovered (GenBank accession numbers ABV27216 and ABV27362, respectively), the additionally described CrtB protein was not. However, a geranylgeranyl pyrophosphate synthase (CrtE; ABV27206) was detected in these searches; it is possible that this sequence was misannotated as CrtB in the paper by Bryant *et al.* [43].

16S rRNA gene sequences were obtained using either BLAST searches against each individual genome or directly from GenBank to scaffold carotenoid biosynthetic pathways upon organismal phylogenies. The 16S rRNA gene was chosen primarily because it is most routinely used for organism identification, and therefore many partial sequences were available for organisms for which complete genome sequences were unavailable.

Note that the present analysis includes organisms present in the IMG and GenBank databases as of early 2008. Whereas this obviously limits the present study in that organisms added subsequent to that date are excluded, similar limitations are characteristic of any database-mining exercise and an inevitable bias in any comparative genomics analysis. However, the present dataset captures the bulk of available phylogenetic diversity from which meaningful observations can be drawn with a reasonable degree of confidence to identify major phylogenetic and evolutionary patterns in carotenoid evolution. The present analysis should be viewed as a framework upon which alternative hypotheses can be built and tested, not a comprehensive description of microbial carotenoid biosynthesis. Those researchers particularly interested in carotenoid biosynthesis in a specific organism are referred to the compiled source data in Table S1 for further information.

### Phylogenetic Methods

All sequences were aligned using CLUSTALW v.2.0.5 [44] or CLUSTALX v.1.83 [45]. Alignments were examined visually and obviously aberrant sequences (e.g. those from incomplete draft genome sequences) were omitted. Extreme 5′ and 3′ sequence ends, which were often of uneven length and poorly aligned, were excluded, as were indels present in only one sequence. Other lineage-specific indels were included to maximize phylogenetic signal for intra-clade phylogenies, even at the expense of resolution at deeper nodes. All conclusions discussed in the text are supported by separate analyses using reduced datasets in which all indels were removed (data not shown). Heterodimeric sequences, where present, were trimmed such that only a single domain was included (Table S2). When occurring separately, heterodimeric CrtYcd or CrtYef sequences were fused to match their monomeric homologs and to maximize the phylogenetic signal.

Phylogenetic analyses were conducted primarily using RAxML v.7.0.4 [46] as implemented through the CIPRES web portal (http://www.phylo.org/). In all cases the Jones-Taylor-Thornton (JTT) substitution matrix was used, the proportion of invariant sites estimated automatically and the best scoring tree used for visualization. Preliminary RAxML experiments using other substitution matrices (BLOSUM62, DAYHOFF and WAG) gave equivalent results, albeit with slightly lower median bootstrap values (data not shown). Nucleotide trees were also created using RAxML according to the default parameters, again using the best tree and estimating the proportion of invariant sites. Further experiments using parsimony (PROTPARS, one jumble per replicate) and distance (PROTDIST, Dayhoff PAM matrix and NEIGHBOR, neighbor joining method) tree construction methods implemented in PHYLIP v.3.66, 3.67 or 3.68 [47] also yielded congruent results. Because nodes were often non-equivalent between methods due to differential placement of poorly-supported and deep-branching sequences between methods, bootstrap values obtained using multiple methods cannot be presented on the same tree; parsimony and distance results are therefore not shown for simplicity. Most trees were rooted to their midpoint using RETREE (PHYLIP). In preliminary experiments, trees rooted using basal-branching outgroup sequences were consistently rooted within the same clade in multiple analyses, but with an unclear intra-clade rooting pattern (data not shown). In these experiments, outgroup sequences were selected from a neighboring COG family showing homology over the entire sequence length, as determined using the NCIB Conserved Domain Database [48]. Midpoint-rooted trees were therefore used here to avoid the intra-clade phylogenetic distortions caused by uncertainly placed roots; relevant observations from rooted trees are indicated.

### Statistical Methods

Non-synonymous (d_n_) and synonymous (d_s_) substitution rates were calculated separately using the Nei-Gojobori method with the Jukes-Cantor correction for same-site mutations, as implemented in MEGA v.4.0 [49] and the d_n_/d_s_ calculated in EXCEL for all pair-wise comparisons with d_s_<1.5 (to account for mutational saturation) and d_n_>0.01 (to ensure a sufficient number of informative substitutions), similar to cutoffs used elsewhere [50]. Nucleotide sequences were aligned in MEGA as translated amino acid sequences for this analysis to conserve codon groupings. Two-tailed *P* values were calculated in SPSS v17.0 using the Mann-Whitney U test by comparing all elevated d_n_/d_s_ pair-wise comparisons for a particular carotenoid biosynthetic gene type and phylogenetic lineage to those not elevated, excluding values generated by pair-wise comparison of two sequences with elevated d_n_/d_s_ ratios. To identify putative recombination events, third codon-position, ungapped nucleotide sequence alignments from each cluster were created using MEGA and maximum-likelihood trees were created using the HKY+gamma substitution matrix implemented in PAUP* v.4.0 (Sinauer Associates, Inc. Publishers, Sunderland Massachusetts). Evolutionary rate heterogeneity [51] was determined using 1000 bootstrap replications for each tree using PIST v.1.0 (http://evolve.zoo.ox.ac.uksoftware.html?id=PIST/).

## Results

### Phylogenetic Structure of Microbial Carotenoid Biosynthesis: Phytoene and 4,4′-Diapophytoene Synthases CrtB and CrtM

Phytoene synthase (CrtB) catalyzes the formation of the C_40_ carotenoid phytoene by the head-to-head condensation of two molecules of C_20_ geranylgeranyl pyrophosphate (Figure 1; [52]). Analogously, the 4,4′-diapophytoene synthase CrtM synthesizes the C_30_ carotenoid 4,4′-diapophytoene from two molecules of C_15_ farnesyl pyrophosphate (Figure 1; [53]). These homologous enzymes are conserved in all carotenogenic taxa and together represent the first dedicated step in carotenoid biosynthesis, making them highly informative to determine the overall phylogenetic topology of carotenoid biosynthesis.

**Figure 2:**
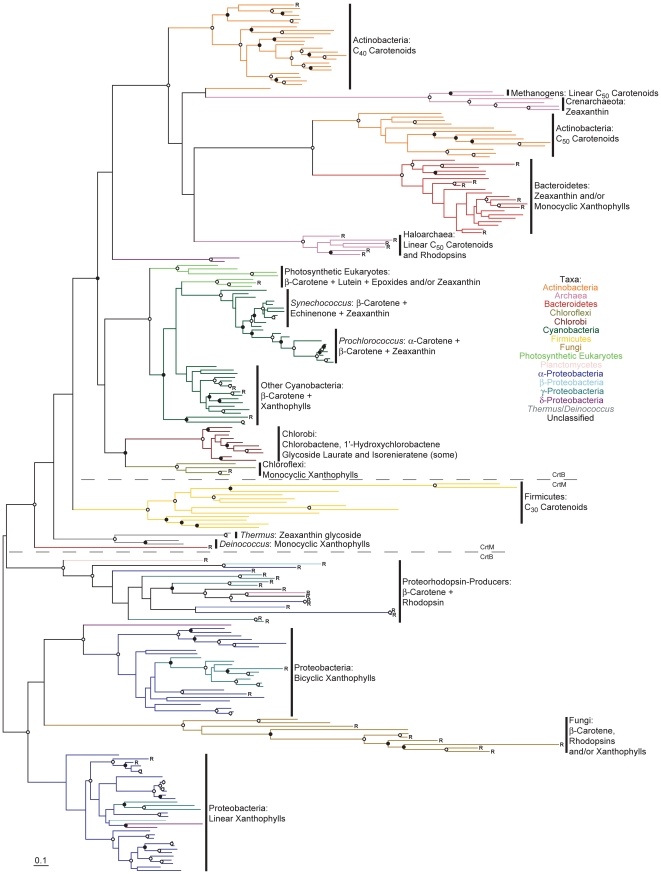
Phylogenetic tree of CrtB and CrtM protein sequences constructed using RAxML. Bootstrap values are indicated as a percentage of the automatically determined number of replicates determined using the CIPRES web portal; those ≥80% are indicated by an open circle and those ≥60% but <80% by a filled circle. For a version of this tree containing sequence names and numerical bootstrap values see Figure S1. Genomes containing a rhodopsin homolog are indicated by an “R”. Carotenoids typical of each lineage are indicated to the right of each clade; note that not all structures are included. The scale bar represents 10% sequence divergence. The tree is rooted to its midpoint to maximize the clarity of intraclade relationships.

A maximum likelihood phylogenetic tree of all analyzed CrtB and CrtM amino acid sequences is shown in Figure 2 (see also Figure S1 in which taxa names and precise bootstrap values are shown). Preliminary experiments using outgroups indicated that CrtM lies at the root of the CrtB/M tree, although which particular CrtM sequence lay closest to the CrtB root remained poorly resolved (data not shown); the tree in Figure 2 is therefore instead rooted to its midpoint for clarity. This tree generally, but not universally, agrees with those generated previously using a much more limited subset of CrtB and CrtM sequences [34], [35]. Where disagreements occur, they are best explained by the much greater numbers of sequences analyzed in the present study compared to those conducted previously. One major exception is the CrtB sequence from *Paracoccus* sp. AC-1 (previously labeled *Agrobacterium aurantiacum*), which clusters strongly with other bicyclic xanthophyll-producing Proteobacteria in this analysis (Figure S1) and not on its own, deeply divergent branch as reported previously [34].

Four main CrtB/M phylogenetic lineages can be defined by considering the well-supported deep phylogenetic nodes in Figure 2. One lineage comprises primarily proteobacterial sequences and is composed of four sub-clades comprising fungi, proteorhodopsin-producers, linear and bicyclic xanthophyll-producing Proteobacteria, respectively. A second well supported lineage comprises sequences from Firmicutes, and has an unresolved relationship with sequences from *Deinococcus*/*Thermus* except for their common exclusion from all other lineages. A third lineage comprises sequences primarily from C_40_-carotenoid producing Actinobacteria (hereafter “C_40_ Actinobacteria”), from which descend clades comprises sequences from haloarchaea, Crenarchaeota, methanogens, primarily C_50_-producing Actinobacteria (hereafter “C_50_ Actinobacteria”) and Bacteroidetes. The final lineage comprises the well-supported pairing of sequences from photosynthetic eukaryotes and Cyanobacteria and the less-supported pairing of sequences from Chlorobi and Chloroflexi. This latter pairing has been recovered to some extent by others [35], [54]. Particularly interesting in this fourth lineage is the well supported basal branching of sequences from red algae in relation to those from green algae and Cyanobacteria. Similar observations have been make previously [55], although in this study all trees were arbitrarily rooted between Cyanobacteria and photosynthetic eukaryotes. This result obviously requires confirmation, although this is outside of the scope of the current study.

### Phylogenetic Structure of Microbial Carotenoid Biosynthesis: Phytoene Synthase CrtI

Phytoene is desaturated in most bacteria by the phytoene desaturase CrtI to produce lycopene (4 desaturations; Figure 1; [56], [57]) or, in spheroidene and spheroidenone-producing Proteobacteria, neurosporene (3 desaturations; Figure 1; [58]). Analogous to CrtB and CrtM, in C_30_ carotenoid-producing organisms a CrtI homolog CrtN (4,4′-diapophytoene desaturase) desaturates 4,4′-diapophytoene to produce 4,4′-diapolycopene (4 desaturations; Figure 1; [59]) or 4,4′-diaponeurosporene (3 desaturations; Figure 1; [53]). In Cyanobacteria, photosynthetic eukaryotes and Chlorobi, the conversion of phytoene to lycopene involves three separate enzymes: the phytoene desaturase CrtP (PDS in eukaryotes), which converts phytoene to ζ-carotene (3 desaturations different from those producing neurosporene; [60], [61]); the ζ-carotene desaturase CrtQ (ZDS in eukaryotes), which converts ζ-carotene into 7,9,7′,9′-*cis-*lycopene (1 desaturation; [62]); and the 7,9,7′9′-*cis*-lycopene isomerase CrtH (CRTISO in eukaryotes), which converts 7,9,7′9′-*cis*-lycopene into all-*trans* lycopene [63], [64]. A second isomerase converting 9,15,9′-ζ-carotene into 9,9′-ζ-carotene has also been identified in some photosynthetic eukaryotes [65]. Whereas CrtP and CrtQ are highly homologous to each other but only distantly related to CrtI [34], CrtH is more closely related to CrtI and its relatives [63]. A second ζ-carotene (and also neurosporene) desaturase CrtQa was also identified [66]; this enzyme, in contrast with CrtQ, produces all-*trans* lycopene and is more closely related to CrtI and its relatives than CrtP and CrtQ [34], [36]. Unequivocal orthologs of CrtQa have not been identified in any other organism ([36]; see also Table S1), and it is annotated as plasmid-borne in the *Nostoc* PCC 7180 genome sequence (which also contains a CrtQ homolog; Table S1). CrtQ is therefore the major microbial ζ-carotene desaturase, not CrtQa as originally thought [66].

**Figure 3:**
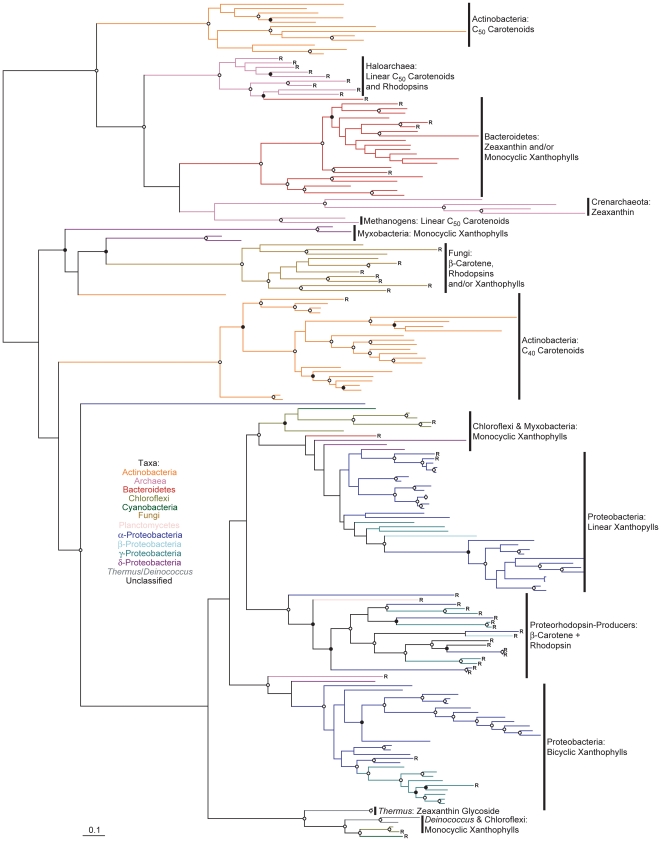
Phylogenetic tree of CrtI protein sequences constructed using RAxML. Bootstrap values are indicated as a percentage of the automatically determined number of replicates determined using the CIPRES web portal; those ≥80% are indicated by an open circle and those ≥60% but <80% by a filled circle. For a version of this tree containing sequence names and numerical bootstrap values see Figure S2. Genomes containing a rhodopsin homolog are indicated by an “R”. Carotenoids typical of each lineage are indicated to the right of each clade; note that not all structures are included. The scale bar represents 10% sequence divergence. The tree is rooted to its midpoint to maximize the clarity of intraclade relationships.

A phylogenetic tree of CrtI is shown in Figure 3 (see also Figure S2 in which taxa names and precise bootstrap values are shown). The CrtI phylogeny is split with strong bootstrap support into two principal lineages (Figure 3), congruent with those determined for CrtB (Figure 2). Note that this phylogeny lacks Chlorobi and most Cyanobacteria due to the presence of CrtP, CrtQ and CrtH instead of CrtI in these taxa (see Figures S3 and S4 for their phylogenies). One CrtI lineage comprises primarily proteobacterial sequences, with sub-clades comprising sequences from proteorhodopsin-producers, bicyclic and linear xanthophyll-producing Proteobacteria. This latter clade clusters strongly with sequences from Chloroflexi and the cyanobacterium *Gloeobacter*, at variance with their position in the CrtB phylogeny (Figure 2); this unusual clustering pattern has been obtained by others previously [67]. The other major CrtI lineage includes clade of sequences from C_40_ Actinobacteria, C_50_ Actinobacteria, haloarchaea, Bacteroidetes, Crenarchaeota and methanogens. Where high bootstrap values are present in both trees, the branching order in this second major CrtI lineage differs from that observed for CrtB (although the C_40_ Actinobacteria is basal in both), as does the phylogenetic position of the fungi (Figures 2 and 3). Again, some, but not all, of these clades have been recognized previously [34], [35].

Phylogenetic trees for CrtP, CrtQ and CrtH were also constructed (Figures S3 and S4) and are almost entirely consistent with the CrtB phylogeny. The one notable observation from these trees is that sequences from the Acidobacterium “*Candidatus* Chloracidobacterium thermophilum” cluster closest those from Chlorobi. This is congruent with phylogenies of the type I photosynthetic reaction centre protein PscA determined for these organisms [43]. “*Candidatus* Chloracidobacterium thermophilum” is known to produce both echinenone and canthaxanthin in culture [68], but the biosynthesis of these compounds in this organism remains otherwise unknown.

In summary, CrtB, CrtI, CrtP, CrtQ and CrtH phylogenies all suggest the same phylogenetic subdivisions, the membership of which corresponds well to the distribution of carotenoid structural types that they are known or inferred to produce. This phylogenetic structure therefore forms a valid framework to address more specific questions concerning microbial carotenoid evolution. Several of these more detailed analyses are presented below.

### Evolution of Microbial Carotenoid Biosynthetic Protein Families: CrtI and its homologs

One notable feature of carotenoid biosynthesis is the multitude of biochemically-distinct CrtI-family enzymes. The biochemistry of these enzymes has been well-studied and their evolution discussed extensively from this perspective (e.g., [36]). CrtI-family enzymes include the previously discussed desaturases CrtI, CrtN and CrtQa and the isomerase CrtH. Other variants include the 3,4-dihydro-1-hydroxy-ψ-end group desaturase CrtD [56], [69], [70], the β-end group ketolase CrtO [71] and the 4,4′-diaponeurosporene and 4,4′-diapolycopene oxidase CrtNb ([59], [72]; confusingly labeled CrtP by Pelz *et al.*), which produces an aldehyde or carboxylic acid, depending on the organism. Additionally, *Myxococcus xanthus* contains two CrtI homologs which are responsible for different steps in the desaturation of phytoene to lycopene [73]. All these CrtI-family members have only limited sequence homology to CrtP and CrtQ and their relative, the β-ionone desaturase CrtU [74]; these latter sequences are therefore not considered further here.

**Figure 4:**
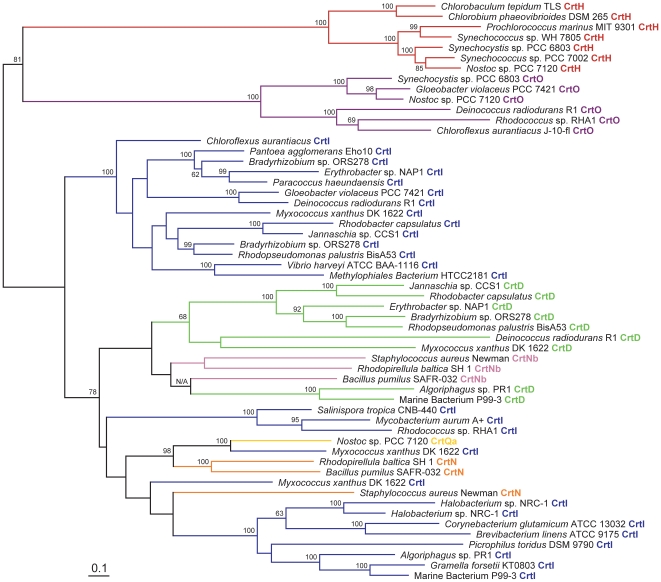
Phylogenetic tree of representative CrtD, CrtH, CrtI, CrtN, CrtNb, CrtO and CrtQa protein sequences constructed using RAxML. Protein types are color-coded and indicated to the right of the sequence name. Bootstrap values ≥60% are indicated as a percentage of the automatically determined number of replicates determined using the CIPRES web portal. The scale bar represents 10% sequence divergence. The tree is rooted to its midpoint to maximize the clarity of intraclade relationships.

Perhaps surprisingly, there exists no published phylogenetic tree containing together all CrtI-family enzymes, despite longstanding knowledge of their shared homology (CrtO and CrtH are most typically excluded; e.g., [34], [35]. Representative members from the current dataset (see Figures 2, 3, S4, S8 and [75] for the rationale behind their selection) were used to construct such a tree (Figure 4), rooted here to its midpoint because using CrtU, CrtP and CrtQ as roots yielded low bootstrap values at the root node. Both CrtH and CrtO formed monophyletic clades related to each other with high bootstrap support, suggesting their ancient paralogous divergence and subsequent conservation of function. Parsimony suggests that the ancestor of these proteins was of the Chlorobi-Cyanobacteria lineage, perhaps existing prior to its acquisition of CrtP and CrtQ. According to this model, CrtO was acquired later by *Rhodococcus*, *Chloroflexus* and *Deinococcus* via horizontal transfer. Contrariwise, CrtI is not monophyletic, including CrtD, CrtN, CrtNb and CrtQa sequences as sister or interspersed lineages. Surprisingly, CrtD did not originate due to paralogous gene duplication of CrtI within a presently CrtD-comprising lineage and instead clusters with CrtN, CrtNb, CrtQa and CrtI from the Actinobacteria/Archaea/Bacteroidetes lineage. Possible explanations for this result include the early evolution of CrtD prior to the divergence of CrtI-comprising sub-lineages or horizontal transfer from either the Proteobacteria or Actinobacteria/Archaea/Bacteroidetes lineage.

In summary, protein phylogenies suggest a much more complicated evolution of CrtI-family desaturases compared to that recognized previously [36], including multiple instances of paralogous gene duplication and divergence, horizontal transfer and differential loss between phylogenetic lineages. This analysis provides a solid phylogenetic framework upon which the extensively researched biochemistry of these proteins can be overlaid.

### Evolution of Microbial Carotenoid Biosynthetic Protein Families: Carotenoid Cyclases

Carotenoid cyclases form a second major carotenoid biosynthetic protein family. Like CrtI-family desaturases, carotenoid cyclases can have multiple, varied functions including the formation of one or two β- and/or ε-ionone-type rings in either C_40_ or C_50_ carotenoids (Figure 1). However, unlike CrtI-family desaturases, multiple, non-homologous cyclases exist which catalyze equivalent biochemical reactions. The evolutionary rationale behind this diversity has been discussed extensively, albeit primarily from a biochemical perspective [34], [37].

Three unique types of carotenoid cyclases are currently known, each of which can be divided further into sub-types. The β-bicyclase CrtY was the first cyclase described [57] and was subsequently shown to be homologous to the cyanobacterial cyclase CrtL [76] and cyclases from photosynthetic eukaryotes [37]. Monocyclic CrtY and CrtL cyclases are also known [77], [78]. Two different CrtL types, CrtLb and CrtLe, occur in some Cyanobacteria, where they function as β- and ε-cyclases, respectively [79]; functionally similar proteins also exist in many photosynthetic eukaryotes [37]. Secondly, CrtYcd-type cyclases are known from Actinobacteria, Archaea and Bacteroidetes, in which they occur either as two proteins (CrtYc and CrtYd; [79]) or a single CrtYcd peptide ([80], [81]; the latter is a monocyclase). CrtYcd homologs from fungi have also described fused to a phytoene synthase [82]. CrtYef and LitAB are homologous to CrtYcd and form ε- and β-ionone-type rings, respectively, in C_50_ carotenoids [83], [84]. Finally, CruA-type cyclases have been described in Cyanobacteria and Chlorobi [85], including the lycopene mono- and bicyclases CruA and CruP [85] and the γ-carotene cyclase CruB [86].

**Figure 5:**
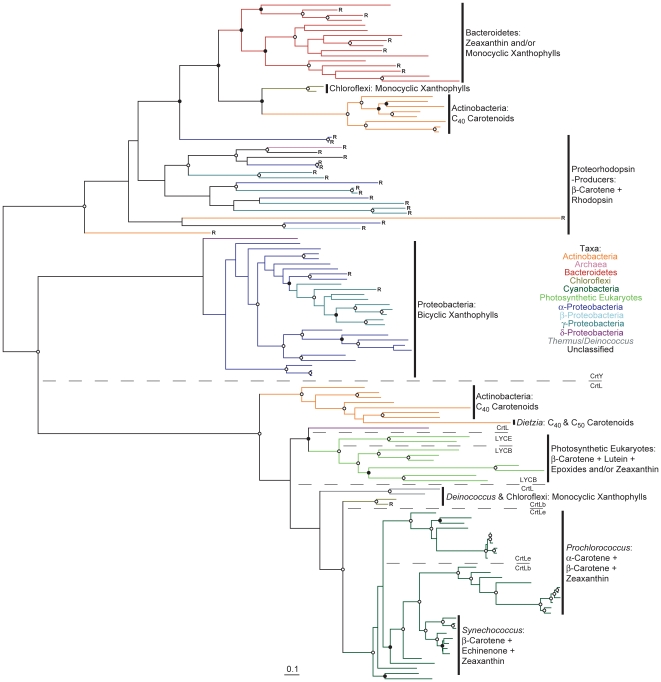
Phylogenetic tree of CrtL and CrtY protein sequences constructed using RAxML. Bootstrap values are indicated as a percentage of the automatically determined number of replicates determined using the CIPRES web portal; those ≥80% are indicated by an open circle and those ≥60% but <80% by a filled circle. For a version of this tree containing sequence names and numerical bootstrap values see Figure S5. Genomes containing a rhodopsin homolog are indicated by an “R”. Carotenoids typical of each lineage are indicated to the right of each clade; note that not all structures are included. The scale bar represents 10% sequence divergence. The tree is rooted to its midpoint to maximize the clarity of intraclade relationships.

**Figure 6:**
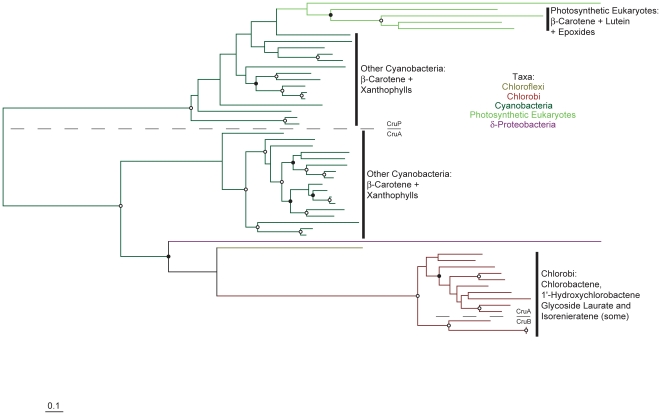
Phylogenetic tree of CruA, CruB and CruP protein sequences constructed using RAxML. Bootstrap values are indicated as a percentage of the automatically determined number of replicates determined using the CIPRES web portal; those ≥80% are indicated by an open circle and those ≥60% but <80% by a filled circle. For a version of this tree containing sequence names and numerical bootstrap values see Figure S6. Genomes containing a rhodopsin homolog are indicated by an “R”. Carotenoids typical of each lineage are indicated to the right of each clade; note that not all structures are included. The scale bar represents 10% sequence divergence. The tree is rooted to its midpoint to maximize the clarity of intraclade relationships.

**Figure 7:**
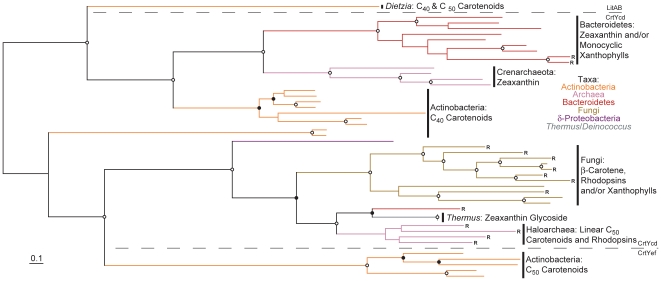
Phylogenetic tree of CrtYcd, CrtYef and LitAB protein sequences constructed using RAxML. Fungal bifunctional proteins and LitBC have been trimmed (see Table S2) and, where applicable, individual CrtYc and CrtYd or CrtYe and CrtYf proteins fused to facilitate comparison of equivalent sequences. Bootstrap values are indicated as a percentage of the automatically determined number of replicates determined using the CIPRES web portal; those ≥80% are indicated by an open circle and those ≥60% but <80% by a filled circle. For a version of this tree containing sequence names and numerical bootstrap values see Figure S7. Genomes containing a rhodopsin homolog are indicated by an “R”. Carotenoids typical of each lineage are indicated to the right of each clade; note that not all structures are included. The scale bar represents 10% sequence divergence. The tree is rooted to its midpoint to maximize the clarity of intraclade relationships.

Carotenoid cyclase evolution involves both extensive horizontal gene transfer and paralogous duplication followed by functional divergence (Figures 5, 6 and 7; see also Figures S5, S6 and S7 in which taxa names and precise bootstrap values are shown). Independent gene duplications and subsequent divergence have likely generated paralogous CrtL-type β- and ε-cyclases in both *Prochlorococcus* and photosynthetic eukaryotes (Figure 5). The cyanobacterial bi- and monocyclases, CruA and CruP respectively [85], are also paralogs which likely diverged early in their evolution (Figure 6). Further paralogous duplication and divergence of CruA within the Chlorobi allowed evolution of the γ-carotene cyclase CruB in some strains [86]. Contrariwise, no obvious paralogy exists for CrtYcd-type cyclases (Figure 7). Here, functional divergence has likely occurred instead between orthologs and/or xenologs (i.e., horizontally transferred orthologs; e.g., LitAB).

Based upon the overarching phylogenies of CrtB, CrtI, CrtP and CrtQ (Figures 2, 3 and S3), which together define the phylogenetic topology of carotenoid biosynthesis as discussed above, parsimony analysis can be applied to the evolution of carotenoid cyclase distribution. CruA-type cyclases most parsimoniously evolved at the base of the Cyanobacteria/Chlorobi lineage, based upon their presence in both the “other Cyanobacteria” and the Chlorobi (Figure 6); both of these clades branch basal to *Prochlorococcus* and *Synechococcus* in CrtB, CrtP, CrtQ and CrtH phylogenies (Figures 2, S3 and S4) indicating their evolutionarily more ancient position within the Cyanobacteria/Chlorobi lineage. CruA was likely displaced in the *Prochlorococcus*/*Synechococcus* lineage due to horizontal transfer of CrtL from the CrtL-comprising C_40_ Actinobacteria lineage or its descendents (Figure 5). Similarly, the presence of CrtYcd-type cyclases throughout the entire Actinobacteria/Archaea/Bacteroidetes lineage may suggest the ancestral presence of CrtYcd-type cyclases within it (Figure 7). However, it is also possible that CrtL was ancestral within the Actinobacteria/Archaea/Bacteroidetes lineage, based upon its deep branching position within the CrtY/L tree (Figure 5), with CrtYcd evolving later elsewhere and being subsequently horizontally transferred into Actinobacteria/Archaea/Bacteroidetes lineage to replace CrtL. This latter scenario is consistent with the relatively long branch length separating the C_40_ Actinobacteria CrtL sequences from Proteobacteria CrtY sequences (Figure 5), as expected from the deep division between the Proteobacteria and Actinobacteria/Archaea/Bacteroidetes lineage in the CrtB and CrtI trees (Figures 2 and 3). Contrariwise, the relatively short branch lengths separating the C_40_ Actinobacteria and Bacteroidetes CrtY sequences from those of proteorhodopsin-producers (Figure 5) is not congruent with the more distant relationship between these taxa in the CrtB and CrtI trees (Figures 2 and 3); this instead suggests horizontal transfer of CrtY from a proteorhodopsin-producer into the C_40_ Actinobacteria and Bacteroidetes. Horizontal gene transfer likely also accounts for the heterogeneous distribution of cyclase types in *Deinococcus*, *Thermus* and Chloroflexi.

In summary, the evolution of carotenoid cyclases is very complex, featuring both paralogous functional diversification and horizontal transfer. Consideration only of carotenoid cyclase biochemistry and not their phylogenies, especially relative to other core carotenoid biosynthesis proteins, masks much of these proteins' diversification. Despite extensive research, the rationale behind the existence of multiple cyclase families still remains unclear. It is possible that functional equivalence between cyclase types might make them especially prone to horizontal gene transfer compared to other carotenoid biosynthetic proteins, leading to the repeated fixation of one cyclase type in a lineage at the expense of another preexisting type. Unfortunately, biochemical properties relevant to this hypothesis (e.g., co-factor requirements of different cyclase types) are known in too few cases to be informative.

### Lineage-Specific Evolutionary Mechanisms of Microbial Carotenoid Biosynthesis: Horizontal Transfer

**Figure 8:**
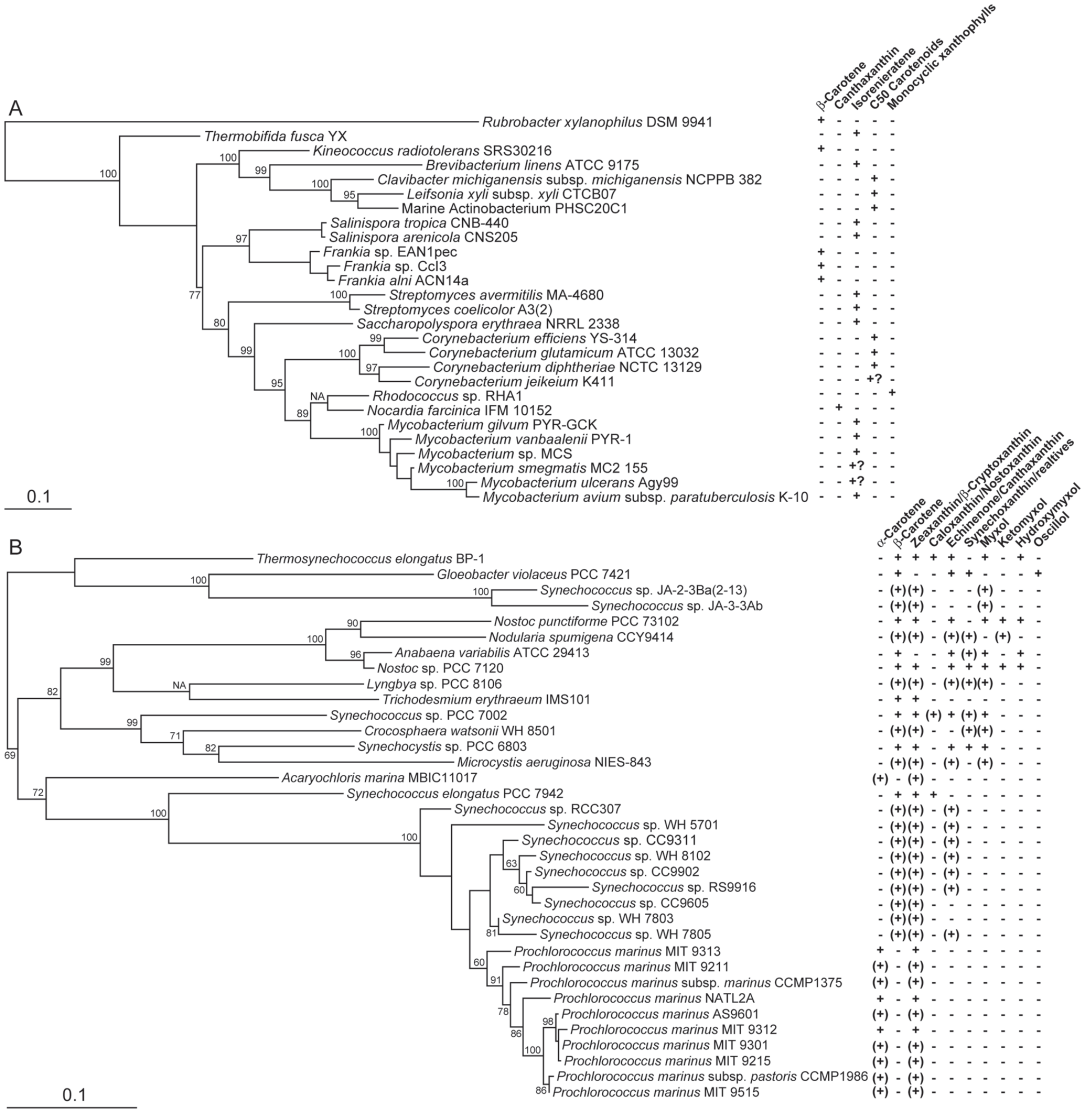
Phylogenetic trees constructed from nearly full-length 16S rRNA genes from carotenoid-producing members of (A) Actinobacteria and (B) Cyanobacteria constructed using RAxML. Bootstrap values ≥60% are indicated as a percentage of the automatically determined number of replicates determined using the CIPRES web portal. All trees are rooted to their midpoint, and the scale bar represents 10% sequence divergence. NA indicates the ML basal node for which no bootstrap value was given. Question marks indicate organisms for which carotenoid biosynthetic pathways are incomplete, likely from genomic decay. For Cyanobacteria, known carotenoids are derived from the compilations of Maresca et al. [31] and Takaichi and Mochimaru [33], with inferences derived from in silico pathway reconstructions (Table S1) indicated in brackets. For Actinobacteria, carotenoid pathway products are nearly exclusively derived from pathway reconstructions (Table S1) due to the lack of 16S rRNA genes for most biochemically studied strains. Note that for clarity, not all terminal pathway modifications (especially glycosylations) are indicated, and carotenoids similarly modified at each end are grouped together because of the difficulty in determining this level of substrate specificity via exclusively in silico analysis.

According to the phylogenetic analyses presented thus far, horizontal transfer is a major diversifying mechanism in microbial carotenoid biosynthesis. This is evident, for instance, from the strongly supported relationship between C_40_ and C_50_ Actinobacteria, Archaea and Bacteroidetes in CrtB, CrtI and CrtYcd phylogenies (Figures 2, 3 and 7). This relationship is highly discordant with the accepted taxonomic separation of these organisms into different super-phyla (e.g., [87]) and strongly suggests horizontal transfer. Similarly, the existence of CrtY-type cyclases in C_40_ Actinobacteria and Bacteroidetes implies horizontal transfer from proteorhodopsin-producers, as discussed above. Other examples of horizontal transfer between C_40_ and C_50_ Actinobacteria are evident by comparing known and inferred carotenoid biosynthesis within these organisms with their 16S rRNA gene phylogeny (Figure 8A). Examples include isorenieratene (C_40_) production in *Brevibacterium* of the C_50_ lineage due to the presumed displacement of the lycopene elongase CrtEb and CrtYef (together leading to cyclic C_50_ carotenoid biosynthesis) by a C_40_ lineage β-carotene desaturase CrtU. Another example is the transfer of a C_40_ linage CrtL into the C_50_ lineage member *Dietzia* sp. CQ4 (Figure 3) enabling canthaxanthin (C_40_) production; in this case the C_50_ carotenoid C.p.450 is still produced [84]. The production of 4-keto-γ-carotene by *Rhodococcus* and canthaxanthin by *Nocardia* and *Dietzia* also suggests horizontal transfer of the ketolase CrtO from distant lineages (Figures 4 and S8). Finally, the clustering of *Corynebacterium* with C_40_ Actinobacteria in the 16S rRNA gene tree (Figure 8A) suggests further horizontal transfer of C_50_ carotenoid biosynthetic genes into this taxon.

In contrast to Actinobacteria, horizontal gene transfer occurs only sporadically within other carotenoid biosynthetic lineages. In Cyanobacteria, the only unequivocal example of horizontal transfer is in *Nodularia spumigena* CCY9414, where CrtP has been replaced by a related ortholog, likely without phenotypic divergence. Similarly, the only unequivocal example of horizontal transfer within the linear xanthophyll-producing Proteobacteria is that of CrtA from the Bacteroidetes, in which it functions as a hydroxylase, into *Rubrivivax gelatinosus* and *Hoeflea phototrophica*. CrtA in *R. gelatinosus* performs not one but two hydroxylations followed by water elimination, thereby functioning as a ketolase and producing spheroidenone 2,2′-diketospirilloxanthin (Table S1; [88]); the evolution of this protein has been described in greater detail elsewhere [75]. These lineages, therefore, likely experience relatively low levels of horizontal transfer.

Whereas zeaxanthin is the primary carotenoid produced by most Bacteroidetes and bicyclic xanthophyll-producing Proteobacteria due to the presence of the hydroxylase CrtZ, several organisms within these lineages also or instead produce ketolated carotenoids due to the presence of the ketolase CrtW. However, phylogenies of these proteins (Figures S9 and S10) do not allow clear differentiation between putative horizontal transfer events versus gene gain followed by differential loss, due both to low bootstrap support at the relative nodes and the lack of clear descendant relationships between the included taxa. Likewise, the poor resolution of Bacteroidetes phylogenies makes it difficult to determine whether bicyclic or monocyclic xanthophylls were most likely produced ancestrally in this lineage. The importance of horizontal transfer versus differential gain and loss in the evolution of their carotenoid biosynthetic pathways is therefore currently difficult to ascertain for both Bacteroidetes and bicyclic xanthophyll-producing Proteobacteria.

### Lineage-Specific Evolutionary Mechanisms of Microbial Carotenoid Biosynthesis: Gene Gain Followed by Differential Loss

In contrast to the above discussion, parsimony analysis suggests that xanthophyll biosynthetic pathway distribution in the “other Cyanobacteria” might be better described by differential gene loss than horizontal transfer. The known and inferred distribution of synechoxanthin and various monocyclic xanthophylls is highly sporadic when compared to the 16S rRNA gene phylogeny of the “other Cyanobacteria” (Figure 8B). This uneven distribution contrasts with carotenoid biosynthetic protein phylogenies for these organisms, which are instead highly congruent (Figures 2, 3, 6, S1, S3-S6, S8, S10-S13). The most parsimonious explanation of these results is that monocyclic xanthophylls were produced ancestrally by “other Cyanobacteria”, and synechoxanthin by a subset of these, with subsequent differential gene loss within this lineage. Pathway diversification in “other Cyanobacteria” also occurs by paralogous gene duplication and divergence, such as that for CrtW in *Nostoc punctiforme* PCC 73102 (Figure S10) to accommodate production of both canthaxanthin and ketomyxol [89]. As discussed above, similar paralogous duplications also exist for CrtL- and CruA-type cyclases (Figures 5 and 6) but were not detected in other carotenoid biosynthetic lineages. Paralogous gene duplication and differential gene loss may therefore be prominent mechanisms of pathway evolution within Cyanobacteria.

### Lineage-Specific Evolutionary Mechanisms of Microbial Carotenoid Biosynthesis: Co-Evolution with Other Biochemical Structures

Carotenoid biosynthetic pathway diversification may not only be fostered by particular evolutionary mechanisms; it can also be hindered. This is particularly evident in the co-evolutionary relationships displayed by some carotenoid biosynthetic lineages with other biochemical structures, particularly proteorhodopsins and the photosynthetic reaction center. In linear xanthophyll-producing Proteobacteria, conserved sub-lineages exist comprising organisms producing as end products either spheroidenone or spirilloxanthin [75]. The membership of the spirilloxanthin-producing lineage is particularly diverse, containing representatives from the α- β- and γ-Proteobacteria [75], [90]. This pattern reflects horizontal transfer of the entire photosynthetic gene supercluster, which includes carotenoid biosynthetic genes, between different subgroups of the Proteobacteria [91], [92]. Evolution of this carotenoid biosynthetic pathway, therefore, does not principally involve an expansion of carotenoid structural diversity (being constrained by the obligation to interact productively with the photosynthetic reaction center) but instead involves expansion of the taxa in which the pathway occurs in conjunction with purple bacterial phototrophy. Note, however, that there exist many other carotenoids known to be produced by purple bacteria with unknown biosynthetic pathways [90]; the extent to which these carotenoids co-evolve with the photosynthetic reaction center remains unknown.

A second carotenoid biosynthesis lineage clearly co-evolving with another biochemical structure is that comprising proteorhodopsin-producing organisms. In this case, further diversification of carotenoid biosynthesis is constrained by the obligation of this lineage to provide the apocarotenoid retinal, a β-carotene cleavage product, as a cofactor critical to proteorhodopsin function [11]. Like proteobacterial-type phototrophy (see above), proteorhodopsins and their associated carotenoid biosynthetic genes have been extensively transferred between taxa (Figures 2, 3 and 5; [12], [41]). Whereas shuffling of genes within this cluster can be detected (e.g., clone HF10_29C11; Figures 2 and 3; [41]), this process appears to be less frequent than horizontal transfer of the entire cluster. Again, co-evolution of carotenoids with other biochemical structures expands the breadth of carotenoid-containing taxa but not carotenoid structural diversity.

In related studies, Sharma et al. [12], [42] performed phylogenetic analyses of microbial rhodopsins. They obtained two major lineages of rhodopsin evolution, one comprising sequences from haloarchaea and fungi and another proteorhodopsins. Carotenoid biosynthetic proteins form similar clusters, albeit alongside other lineages not typified by the presence of rhodopsins (Figures 2 and 3; “R” designates the presence of a rhodopsin homolog in that organism). Interestingly, the rhodopsins clustering phylogenetically nearest the proteorhodopsins [12], [42] (as opposed archaeal and fungal rhodopsins) are from organisms that are widely distributed in CrtB and CrtI trees; these include *Nostoc* sp. PCC 7120, *Gloeobacter violaceus*, *Kineococcus radiotolerans*, *Rubrobacter xylanophilus* and the *Bacteroidetes* (Figures S1 and S2). In these cases, the lack of co-clustering between rhodopsins and carotenoid biosynthetic genes suggests that retinal production evolved by co-opting a preexisting carotenoid biosynthetic pathway. The proteorhodopsin progenitor therefore likely underwent numerous horizontal transfers as a single gene before its linkage with a specific carotenoid biosynthetic lineage, following which it was transferred as part of the proteorhodopsin gene cluster, constraining carotenoid biosynthesis in this lineage from further diversification due to retinal production. Co-evolution of rhodopsins and carotenoid biosynthetic proteins also occurred in fungi and archaea, although with greater carotenoid diversification, perhaps accommodated in part by carotenoid biosynthetic gene duplication. Inclusion of actinorhodopsins [42] in this evolutionary model will be especially interesting once their cognate carotenoid biosynthetic protein sequences are available.

Lastly, cyanobacterial carotenoid biosynthetic proteins are also expected to evolve in conjunction with the cyanobacterial photosynthetic reaction centre due to their intricate involvement with the photochemistry of this structure [93], [94]. However, cyanobacterial carotenoid biosynthesis has continued to diversify despite this structural obligation, as described above. This paradox might be reconciled by functional non-equivalence of cyanobacterial carotenoids. In support of this hypothesis, β-carotene was the only carotenoid present in the crystal structures of the cyanobacterial photosynthetic reaction centre [93], [94], and several carotenoids have been shown to partition differentially into various cyanobacterial membrane and cytosolic fractions [95]. In Cyanobacteria, therefore, constriction of carotenoid diversification due to interaction with the photosynthetic reaction centre may be evaded by partitioning of different carotenoid structures into different functional roles. The regulatory mechanisms which might allow such diversification (e.g., by creating multiple β-carotene pools) remain unknown.

### Lineage-Specific Evolutionary Mechanisms of Microbial Carotenoid Biosynthesis: Selection

Positive evolutionary selection may increase carotenoid biosynthetic protein diversity by selecting for altered protein functions leading to evolutionarily advantageous phenotypes, especially following gene duplication or horizontal transfer. This phenomenon is detectable as an elevated non-synonymous/synonymous nucleotide substitution ratio (d_n_/d_s_; [96]). Genes for each protein type and carotenoid biosynthetic lineage were compared in a pair-wise manner, considering only d_n_ values >0.1 to ensure sufficient sequence variation and d_s_ values <1.5 to account for mutational saturation due to divergence (i.e., resulting from back mutations; [96]), in general agreement with cutoffs used elsewhere [50]. These cutoff levels, while eliminating obviously aberrant comparisons, also resulted in rejection (due to d_s_ values >1.5) of most comparisons of cyanobacterial sequences, many of which are obviously only minimally divergent (Figures 2, 5 and 6). Sequence comparisons within these groups also showed low d_n_ values suggesting strong negative selection operating on these genes. Selection in the evolution of carotenoid biosynthesis in the purple bacteria is analyzed in greater detail elsewhere [75] and therefore is considered only briefly here.

**Figure 9:**
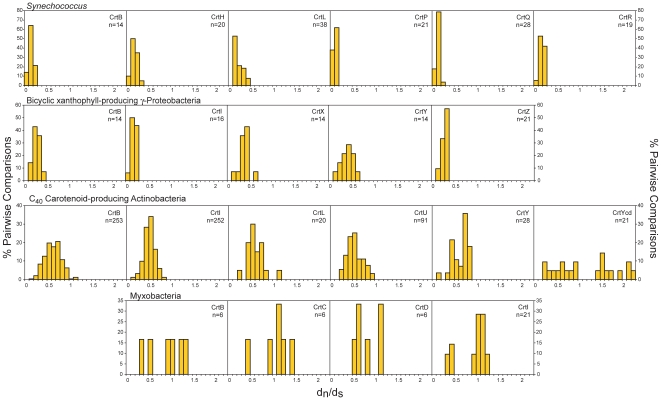
Distributions of pair-wise d_n_/d_s_ values, rounded to one decimal place, for *Synechococcus*, bicyclic xanthophyll-producing γ-Proteobacteria, C_40_ Actinobacteria and myxobacteria, expressed as a percentage of the total number of comparisons (n) for each sequence cluster protein. Only values with d_n_>0.01 and d_s_<1.5 were included; note that these cut-offs underestimate values at the lower range of the distributions shown, especially for *Synechococcus*. Results for other taxa are shown in Figure S14.

To determine potentially lineage-specific evolutionary mechanisms in carotenoid biosynthesis, pair-wise d_n_/d_s_ comparisons were binned by rounding to one decimal place and the frequency of each value plotted (Figures 9 and S14). Positive selection upon sequences within these datasets was inferred if the resulting distribution was bimodal (as opposed to unimodal if selection was approximately uniform among the sequences analyzed) with one peak centered about a value of 1 or greater. Upon detection, the original pair-wise matrices were examined to determine the sequence(s) that might be responsible for the elevated values (Figure S15) which were then compared statistically with non-elevated values from the same lineage (Table 1). This approach was chosen over other, more statistically informative analyses such as codeml [97] due to its better accommodation of divergent sequences and lower demand for computational resources required by the large datasets analyzed in this study. Using this method, d_n_/d_s_ ratios >1 were detected for *Mycobacterium aurum* A+ and *Frankia alni* ACN14a *crtYcd*, *Dietzia* sp. CQ4 *crtYef* and all carotenoid biosynthetic genes from *Stigmatella aurantiaca* DW4/3-1 and *Myxococcus xanthus* DK 1622 (Table 1 and Figures 9, S14 and S15). Therefore, elevated d_n_/d_s_ ratios can occur either for specific genes (Actinobacteria) or for entire pathways and phylogenetic lineages (*Myxococcus* and *Stigmatella*). Intriguingly, *M. xanthus* contains two CrtI proteins responsible for separate desaturations [73], possibly a result of recent divergence due to positive selection. Although its underlying cause remains unclear, CrtI diversification in myxobacteria is consistent with the large genome size and abundance of gene duplications reported for these organisms [98].

**Table 1:** Inferred positive selection on carotenoid biosynthetic genes.

Sequences	d_n_/d_s_ (mean ± standard deviation)^a^	Number of pair-wise comparisons (elevated/non-elevated)	Mann-Whitney U test versus other sequences from the same lineage
*Mycobacterium aurum* A+ and *Frankia alni* ACN14a *crtYcd*	1.75±0.30	10/10	Two-tailed *P = *0.000; Z = −3.780
*Dietzia* sp. CQ4 *crtYef*	1.05±0.08	3/5	Two-tailed *P = *0.025; Z = −2.236
*Myxococcus xanthus* DK 1622 and *Stigmatella aurantiaca* DW4/3-1 *crtB*	1.11±0.17	4/1	-b
*Myxococcus xanthus* DK 1622 and *Stigmatella aurantiaca* DW4/3-1 *crtC*	1.20±0.15	4/1	-
*Myxococcus xanthus* DK 1622 and *Stigmatella aurantiaca* DW4/3-1 *crtD*	1.05±0.03	4/1	-
*Myxococcus xanthus* DK 1622 and *Stigmatella aurantiaca* DW4/3-1 *crtI*	1.05±0.08	16/3	Two-tailed *P = *0.007; Z = −2.683

*^a^*Pair-wise comparisons between sequences with elevated d_n_/d_s_ ratios were aberrantly low and excluded from this calculation; see Figure S15.

*^b^*Insufficient number of sequences available for statistical comparison.

Aside from the evidence of positive selection highlighted above, differences between the overall d_n_/d_s_ ratios over the entire pathway between phylogenetic groups were also detected, albeit with the caveats concerning the conservativeness of the d_s_ cutoffs used and methodological accommodations for the divergent sequences analyzed. Considering all carotenoid biosynthetic pathway genes together, d_n_/d_s_ ratios were lowest in Cyanobacteria (d_n_/d_s_ centered about ≈0.1–0.2; Figures 9 and S14), followed by the spheroidenone-producing Proteobacteria, bicyclic xanthophyll-producing γ-Proteobacteria, *Sphingomonadales* and Bacteroidetes (d_n_/d_s_ centered about ≈0.2–0.3; Figures 9 and S14) and finally, spirilloxanthin-producing Proteobacteria, bicyclic xanthophyll-producing α-Proteobacteria, proteorhodopsin-producers, *Deinococcus-Thermus*, haloarchaea, Firmicutes and C_40_ and C_50_ Actinobacteria (d_n_/d_s_ centered about ≈0.4–0.5; Figures 9 and S14). While not considered in greater detail here, the differences in selection operative on the carotenoid biosynthetic pathways of different phylogenetic lineages is clearly a topic for future study. Interestingly, differences between d_n_/d_s_ ratios for different pathway steps were not apparent, in contrast to the plant anthocyanin pathway [99], [100]. Whether this is a general feature resulting from the metabolic pathway topology of carotenoid biosynthesis might also benefit from future study.

### Lineage-Specific Evolutionary Mechanisms of Microbial Carotenoid Biosynthesis: Recombination

One striking feature of all phylogenetic trees analyzed in this study was the poor bootstrap support for the Chlorobi and Bacteroidetes lineages. A similar result reported by others was attributed to low levels of phylogenetically informative sequence positions despite long branch lengths [86]. While bootstrap values were improved in maximum likelihood phylogenies considering only Chlorobi sequences, this was not true of Bacteroidetes CrtB, CrtI and CrtZ trees (data not shown). Interestingly, a recent study identified *Flavobacterium psychrophilum* as having the highest recombination rate of all tested organisms [101]. To determine the impact of recombination on the evolution of carotenoid biosynthetic pathways, the heterogeneous rate test [51] was applied to the same sequence groups used for d_n_/d_s_ calculation. In nearly all cases the ratio of two-state parsimony-informative sites to all polymorphic sites (*q*) was <0.35 (average *q* = 0.24) with low associated *P* values (data not shown), indicating that homologous recombination was not detected by this method, and therefore likely plays only a minor role in microbial carotenoid biosynthetic pathway evolution.

## Discussion

Carotenoids are undoubtedly best studied in their roles as antioxidants and accessory photosynthetic pigments. Accordingly, carotenoid structural and biosynthetic diversity has been especially well studied in purple bacteria and Cyanobacteria [31], [33], [90]. As argued previously [28], the study of non-photosynthetic microbes has hitherto lacked the same degree of systematization and has instead focused on the novel carotenoids and biosynthetic genes of specific microbes as they are discovered, without determination of the degree to which they are representative of related organisms. This is especially true of numerous studies concerning carotenoid structure which tend to focus on non-model organisms. (This is especially problematic with the older literature, for which correspondence of the studied organisms with currently described taxa is often impossible.) The present study takes the opposite approach, using publicly available genome sequences to determine the potential of diverse taxa to produce carotenoids based on the homology of their encoded genes to those known to be involved in carotenoid biosynthesis. Despite certain limitations (see methods; note also the neglect of esterifications and the lacking specification of enzymatic transformation of one versus both carotenoid ends during *in silico* biosynthetic pathway reconstruction; Table S1), comparative genomics is currently one of the best methods for studying pathway diversity because it allows hypotheses of novel diversity to be formulated based upon apparent knowledge gaps, and for phylogenetic relatedness and evolutionary patterns to be qualitatively determined.

Building on (and in some cases, in contrast to) related studies conducted previously [34], [35], the phylogenies presented here delineate four major lineages of carotenoid evolution composed of: (i) Firmicutes; (ii) Cyanobacteria, Chlorobi and photosynthetic eukaryotes; (iii) linear and bicyclic xanthophyll-producing Proteobacteria and proteorhodopsin-producers; and (iv) C_50_ Actinobacteria, C_40_ Actinobacteria, Archaea and Bacteroidetes. In addition (and not discussed extensively above), genes from several taxa are independent from or associated with more than one of the described lineages; these include sequences from *Deinococcus*/*Thermus*, fungi, *Rubrobacter xylanophilus*, δ-Proteobacteria and Chloroflexi. More study is needed to determine to what extent these divergent sequences fit with this proposed model of carotenoid biosynthetic evolution. This is also true of taxa known to be carotenogenic but without sequenced genomes (at least during data mining for this study), including Acidobacteria [43] and Verrucomicrobia [102]. Surprisingly, carotenogenesis was highly conserved in some analyzed taxa (Figure S16), with putative carotenoid biosynthetic pathways encoded by approximately 1/3 and 2/3 of analyzed Bacilli and Actinobacteria, respectively, and all analyzed Flavobacteria and Sphingobacteria. These results suggest the potentially underappreciated importance of carotenoid biosynthesis in these taxa.

One striking feature of all carotenoid biosynthetic trees generated in this study is the monophyletic clustering of sequences from particular phyla to the exclusion of those from other related phyla. Exceptional in this regard are those sequences which have been horizontally transferred between phyla as part of a larger gene cluster (e.g., alongside proteorhodopsins). These observations suggest that carotenoid biosynthesis is an ancient process, having evolved prior to or concurrent with the diversification of the major organismal phylogenetic lineages. The deviance of carotenoid biosynthetic phylogenies from those typical of “core” genome proteins [87] suggest significant horizontal transfer of the entire biosynthetic pathway during this period (e.g., indicated by the close relationships between Actinobacteria, Archaea and Bacteroidetes; Figures 2, 3 and 7). In some cases, these transfers involved only particular pathway components (e.g., indicated by different branching orders between Actinobacteria, Archaea and Bacteroidetes for CrtB and CrtI; Figures 2 and 3).

Many scenarios for the earliest organisms postulate a heterotrophic lifestyle (the “Oparin-Haldane theory”; [103]), potentially under increased levels of UV radiation [104]. Given the apparently early origin of carotenoid biosynthesis, it is quite plausible that these pigments evolved originally to play a role in membrane stabilization and UV tolerance [10], [105]. Indeed, some have even argued for the emergence of terpenoid lipids (including carotenoids) prior to fatty acids [106]; this scenario particularly posits carotenoids functioning to hold membrane bilayers together as “molecular rivets”. Carotenoid-producing organisms would also be particularly well-adapted to the development of increasingly oxidative conditions (e.g., resulting from photosynthesis), a prime stressor in the evolution of life on Earth. An ancient role of carotenoids as antioxidants is appealing given their ability to autonomously quench oxidative processes (e.g., dissipation of energy from ^1^O_2_ as heat, autoxidation of carotenoid radicals by cleavage or addition along the conjugated double bond chain), although the niche over which carotenoids might convey an adaptive phenotype is bounded in part by the conditions under which carotenoids function pro-oxidatively [2], [19]. The simplicity of these systems, and their potential to be selectively favorable for reasons other than their antioxidative properties, makes a strong case for the involvement of carotenoids in early cellular evolution. Over time carotenoid physiology would have further diversified, in conjunction with the formation of other antioxidant systems (e.g., ascorbic acid; [19]) and/or other structures such as rhodopsins and those involving photosynthesis. The later adaptation of carotenoids to function in photosynthesis is especially supported by the wide variety of carotenoids produced in various photosynthetic taxa: C_40_ linear xanthophylls in purple bacteria; C_30_ linear xanthophylls in Heliobacteria; β-carotene and bicyclic xanthophylls in photosynthetic eukaryotes, Acidobacteria and Cyanobacteria (which also produce monocyclic xanthophylls); and monocyclic xanthophylls in Chlorobi and Chloroflexi. This diversity suggests that carotenoids were co-opted from preexisting structural diversity during the evolution of photosynthesis in these various taxa. Whereas the suggestion from this work that Firmicutes CrtM sequences root the CrtB tree (and therefore, perhaps, carotenoid biosynthesis more generally) is reminiscent of the hypothesis of a heliobacterial (Firmicutes) origin for photosynthesis [107], the presence of similar carotenoids in many non-photosynthetic Firmicutes argues against this being the major selective force during carotenoid evolution in these organisms.

As discussed previously [22], carotenoid biosynthesis can be arranged into a “tree-like” hierarchy based upon structural and biosynthetic interrelations. To what extent does the synthesis presented here reflect this tree-like structure? Core carotenoid biosynthetic proteins (CrtB and CrtI) are highly conserved both functionally and phylogenetically (Figures 2 and 3), consistent with their identification with the “root” of the carotenoid tree-like hierarchy. However, carotenoid biosynthetic gene presence and function in different taxa begins to diverge following these steps, leading to a myriad of biosynthetic “branches”. At this point, the phylogenetic and biosynthetic viewpoints diverge; instead of distinct branches, phylogenetic analysis reveals many web-like evolutionary interactions resulting from extensive horizontal gene transfer, paralogous gene duplication with concomitant functional divergence and differential gene loss; this is especially exemplified by the evolution of carotenoid cyclases. While not well resolved in this present study due to the lack of reference data and genomic sequences at an appropriate depth, terminal biosynthetic enzymes may be especially prone to non-vertical modes of evolution ([28]; consider also cyanobacterial monocyclic xanthophyll biosynthesis), presumably resulting from the minor adaptive significance of these changes. Note, however, that where strong selection exists, such as during co-evolution of carotenoids with the purple bacterial photosynthetic reaction center, terminal biosynthetic pathway steps may be less evolutionarily plastic [75]. I therefore suggest that carotenoid biosynthetic pathway evolution might more representatively be envisioned as a “bramble”, where interior nodes branching from the root are highly reticulated due to non-vertical modes of evolution. Where selection for a particular carotenoid structure is relatively weak, the edge of this structural “bramble” will be ragged and multiple, related structures may coexist in relatively close phylogenetic neighbors. Elsewhere, these “ragged edges” may be trimmed by more intensive selection, resulting in only certain structural types existing in those phyla and restricting their further diversification.

Understanding the evolutionary rationale behind observed phylogenetic patterns in metabolite distribution may be a beneficial approach to understanding their diversity. The homogeneous phylogenetic distribution of a metabolite or biosynthetic pathway may suggest its adaptivity, a testable hypothesis. Reciprocally, phyla within which metabolites or biosynthetic pathways are under relatively weak selection may be excellent candidates to contain novel compounds and/or biosynthetic pathway enzymes with reduced substrate specificity. These may be particularly useful in recombinant biosynthetic pathway construction [22]. Some structures that do not confer a strong selective benefit to their hosts may be strongly adaptive in a different context (e.g., naturally-occurring carotenoids may also function in human nutrition). Indeed, this process is widespread in nature during xenologous gene transfer [108]. Evolution may therefore be understood as an applied concept for biotechnology. Placing future research within this context will undoubtedly be a key to fruitfully understanding and exploiting metabolic diversity.

## Supporting Information

Table S1 Carotenoid biosynthetic protein homologs and the (inferred) products of their corresponding biosynthetic pathways. IMG locus and GenBank accession numbers are indicated in the same order as their corresponding protein sequences. Carotenoids and biosynthetic proteins for which experimental evidence exists are underlined and the corresponding references indicated. Proteins leading to the production of apocarotenoids other than neurosporaxanthin are omitted. Also indicated are the presence of a detected rhodopsin homolog in an organism's genome and whether the genome analysed was completed at the time of study.
(0.75 MB DOC)

Table S2 Start and end amino acids for used in this study for carotenoid biosynthesis fusion proteins.
(0.05 MB DOC)

Table S3 Known microbial carotenoid biosynthetic proteins used for in silico carotenoid biosynthetic pathway reconstruction, their synonyms and biochemical functions.
(0.08 MB DOC)

Figure S1 Phylogenetic tree of CrtB and CrtM protein sequences constructed using RAxML. Bootstrap values ≥60% are indicated as a percentage of the automatically determined number of replicates determined using the CIPRES web portal. Genomes containing a rhodopsin homolog are indicated by an “R” and sequences with genetically or biochemically demonstrated functions are bolded. Carotenoids typical of each lineage are indicated to the right of each clade, with exceptions indicated by asterisks. The scale bar represents 10% sequence divergence. The tree shown is rooted to its midpoint to maximise the clarity of intraclade relationships. NA indicates the ML basal node for which no bootstrap value was given. Due to its extreme branch length the sequence from *Aspergillus niger*, while homologous to all other sequences, was excluded.
(13.78 MB DOC)

Figure S2 Phylogenetic tree of CrtI protein sequences constructed using RAxML. Bootstrap values ≥60% are indicated as a percentage of the automatically determined number of replicates determined using the CIPRES web portal. Genomes containing a rhodopsin homolog are indicated by an “R” and sequences with genetically or biochemically demonstrated functions are bolded. Carotenoids typical of each lineage are indicated to the right of each clade, with exceptions indicated by asterisks. The scale bar represents 10% sequence divergence. The tree shown is rooted to its midpoint to maximise the clarity of intraclade relationships. NA indicates the ML basal node for which no bootstrap value was given.
(9.95 MB DOC)

Figure S3 Phylogenetic tree of CrtP (PDS) and CrtQ (ZDS) protein sequences constructed using RAxML. Bootstrap values ≥60% are indicated as a percentage of the automatically determined number of replicates determined using the CIPRES web portal. Sequences with genetically or biochemically demonstrated function are bolded. Carotenoids typical of each lineage are indicated to the right of each clade, with exceptions indicated by asterisks. The tree shown is rooted to its midpoint, and the scale bar represents 10% sequence divergence. NA indicates the ML basal node for which no bootstrap value was given.
(7.92 MB TIF)

Figure S4 Phylogenetic tree of CrtH (CRTISO) protein sequences constructed using RAxML. Bootstrap values ≥60% are indicated as a percentage of the automatically determined number of replicates determined using the CIPRES web portal. Sequences with genetically or biochemically demonstrated function are bolded. Carotenoids typical of each lineage are indicated to the right of each clade, with exceptions indicated by asterisks. The tree shown is rooted to its midpoint, and the scale bar represents 10% sequence divergence. NA indicates the ML basal node for which no bootstrap value was given.
(3.98 MB TIF)

Figure S5 Phylogenetic tree of CrtY and CrtL protein sequences constructed using RAxML. Bootstrap values ≥60% are indicated as a percentage of the automatically determined number of replicates determined using the CIPRES web portal. Genomes containing a rhodopsin homolog are indicated by an “R” and sequences with genetically or biochemically demonstrated functions are bolded. Carotenoids typical of each lineage are indicated to the right of each clade, with exceptions indicated by asterisks. The scale bar represents 10% sequence divergence. The tree shown is rooted to its midpoint to maximise the clarity of intraclade relationships. NA indicates the ML basal node for which no bootstrap value was given. Because of its long branch length the CrtY sequence for uncultured marine bacterium HF10_49E08, although homologous to other CrtY sequences, was excluded.
(6.97 MB TIF)

Figure S6 Phylogenetic tree of CruA, CruB and CruP protein sequences constructed using RAxML. Bootstrap values ≥60% are indicated as a percentage of the automatically determined number of replicates determined using the CIPRES web portal. Sequences with genetically or biochemically demonstrated functions are bolded. Carotenoids typical of each lineage are indicated to the right of each clade, with exceptions indicated by asterisks. The scale bar represents 10% sequence divergence. The tree shown is rooted to its midpoint to maximise the clarity of intraclade relationships. NA indicates the ML basal node for which no bootstrap value was given.
(4.49 MB TIF)

Figure S7 Phylogenetic tree of CrtYcd, CrtYef and LitAB protein sequences constructed using RAxML. Sequences present as separate subunits were artificially fused prior to alignments. Bootstrap values ≥60% are indicated as a percentage of the automatically determined number of replicates determined using the CIPRES web portal. Genomes containing a rhodopsin homolog are indicated by an “R” and sequences with genetically or biochemically demonstrated functions are bolded. Carotenoids typical of each lineage are indicated to the right of each clade, with exceptions indicated by asterisks. The scale bar represents 10% sequence divergence. The tree shown is rooted to its midpoint to maximise the clarity of intraclade relationships. NA indicates the ML basal node for which no bootstrap value was given.
(3.99 MB TIF)

Figure S8 Phylogenetic tree of CrtO protein sequences constructed using RAxML. Bootstrap values ≥60% are indicated as a percentage of the automatically determined number of replicates determined using the CIPRES web portal. Sequences with genetically or biochemically demonstrated functions are bolded. Carotenoids typical of each lineage are indicated to the right of each clade, with exceptions indicated by asterisks. The scale bar represents 10% sequence divergence. The tree shown is rooted to its midpoint to maximise the clarity of intraclade relationships. NA indicates the ML basal node for which no bootstrap value was given.
(2.01 MB TIF)

Figure S9 Phylogenetic tree of CrtZ protein sequences constructed using RAxML. Bootstrap values ≥60% are indicated as a percentage of the automatically determined number of replicates determined using the CIPRES web portal. Sequences with genetically or biochemically demonstrated functions are bolded. Carotenoids typical of each lineage are indicated to the right of each clade, with exceptions indicated by asterisks. The scale bar represents 10% sequence divergence. The tree shown is rooted to its midpoint to maximise the clarity of intraclade relationships. NA indicates the ML basal node for which no bootstrap value was given.
(4.63 MB TIF)

Figure S10 Phylogenetic tree of CrtW protein sequences constructed using RAxML. Bootstrap values ≥60% are indicated as a percentage of the automatically determined number of replicates determined using the CIPRES web portal. Sequences with genetically or biochemically demonstrated functions are bolded. Carotenoids typical of each lineage are indicated to the right of each clade, with exceptions indicated by asterisks. The scale bar represents 10% sequence divergence. The tree shown is rooted to its midpoint to maximise the clarity of intraclade relationships. NA indicates the ML basal node for which no bootstrap value was given.
(2.89 MB TIF)

Figure S11 Phylogenetic tree of CrtG protein sequences constructed using RAxML. Bootstrap values ≥60% are indicated as a percentage of the automatically determined number of replicates determined using the CIPRES web portal. Sequences with genetically or biochemically demonstrated functions are bolded. Carotenoids typical of each lineage are indicated to the right of each clade, with exceptions indicated by asterisks. The scale bar represents 10% sequence divergence. The tree shown is rooted to its midpoint to maximise the clarity of intraclade relationships. NA indicates the ML basal node for which no bootstrap value was given.
(1.76 MB TIF)

Figure S12 Phylogenetic tree of CrtR protein sequences constructed using RAxML. Bootstrap values ≥60% are indicated as a percentage of the automatically determined number of replicates determined using the CIPRES web portal. Sequences with genetically or biochemically demonstrated functions are bolded. Carotenoids typical of each lineage are indicated to the right of each clade, with exceptions indicated by asterisks. The scale bar represents 10% sequence divergence. The tree shown is rooted to its midpoint to maximise the clarity of intraclade relationships. NA indicates the ML basal node for which no bootstrap value was given.
(3.24 MB TIF)

Figure S13 Phylogenetic trees of (A) CruE, (B) CruF, (C) CruG and (D) CruH protein sequences constructed using RAxML. Bootstrap values ≥60% are indicated as a percentage of the automatically determined number of replicates determined using the CIPRES web portal. Sequences with genetically or biochemically demonstrated functions are bolded. Carotenoids typical of each lineage are indicated to the right of each clade, with exceptions indicated by asterisks. The scale bar represents 10% sequence divergence. The trees shown are rooted to their midpoint to maximise the clarity of intraclade relationships. NA indicates the ML basal node for which no bootstrap value was given.
(2.54 MB DOC)

Figure S14 Distributions of pairwise d_n_/d_s_ values, rounded to one decimal place, for phylogenetic groups described in the text, expressed as a percentage of the total number of comparisons (n) for each sequence cluster protein. Only values with d_n_>0.01 and d_s_<1.5 were included; note that this underestimates the values at the lower end of the distributions shown, especially for Cyanobacteria and Chlorobi. Results for *Synechococcus*, bicyclic xanthophyll-producing γ-Proteobacteria, C_40_ carotenoid-producing Actinobacteria and myxobacteria are shown in Figure 8.
(5.93 MB DOC)

Figure S15 Pairwise d_n_/d_s_ values for: (A) C_40_ carotenoid-producing Actinobacteria *crtYcd*; (B) C_50_ carotenoid-producing Actinobacteria *crtYef* and myxobacterial *crtB* (C), *crtC* (D), *crtD* (E) and *crtI* (F). Matrices are one-sided, with cells of the opposite side filled with a dash. Bolded values are those highlighted in the text. In some cases a pairwise comparison of two sequences otherwise determined to have a high d_n_/d_s_ values yielded an unexpectedly low d_n_/d_s_ value; these ratios are iticized. NC indicates comparisons for which MEGA 4.0 could not calculate d_s_ value.
(0.08 MB DOC)

Figure S16 Distribution of carotenoid biosynthetic pathways (as inferred from Supplementary Table S1) in genome sequences of the IMG database, version 2.4. Except Cyanobacteria, each species was considered only once despite the presence of multiple strains. Because incomplete genomes were included this analysis represents an underestimate.
(1.72 MB TIF)
