# Supplementary material for: Phylogenetic and Evolutionary Patterns in Microbial Carotenoid Biosynthesis Are Revealed by Comparative Genomics
Source: PLoS One. 2010 Jun 22;5(6):e11257. doi: 10.1371/journal.pone.0011257 (PMC2889829; doi:10.1371/journal.pone.0011257)
Supplement: Table S1 — Carotenoid biosynthetic protein homologs and the (inferred) products of their corresponding biosynthetic pathways. IMG locus and GenBank accession numbers are indicated in the same order as their corresponding protein sequences. Carotenoids and biosynthetic proteins for which experimental evidence exists are underlined and the corresponding references indicated. Proteins leading to the production of apocarotenoids other than neurosporaxanthin are omitted. Also indicated are the presence of a detected rhodopsin homolog in an organism's genome and whether the genome analysed was completed at the time of study. (0.75 MB DOC) [file pone.0011257.s001.doc]

Table S1. Carotenoid biosynthetic protein homologs and the (inferred) products of their corresponding biosynthetic pathways. IMG locus and GenBank accession numbers are indicated in the same order as their corresponding protein sequences. Carotenoids and biosynthetic proteins for which experimental evidence exists are underlined and the corresponding references indicated. Proteins leading to the production of apocarotenoids other than neurosporaxanthin are omitted. Also indicated are the presence of a detected rhodopsin homolog in an organism’s genome and whether the genome analysed was completed at the time of study.

| **Organism** | **Carotenoid biosynthesis protein homologs^a^** | **IMG Loci (plain text) or GenBank (italics) identifiers** | **Major carotenoid(s) produced^a, b^** | **Rhodopsin^a^** | **Complete genome** | **References** |
| --- | --- | --- | --- | --- | --- | --- |
| **α-Proteobacteria** | | | | | | |
| **Caulobacterales** | | | | | | |
| *Brevundimonas* sp. SD212 | CrtB, CrtG, CrtI, CrtW, CrtY, CrtZ | *BAD99409*, *BAD99415*, *BAD99408*, *BAD99406*, *BAD99407*, *BAD99414* | 2-Hydroxyastaxanthin | N/A | N/A | [1,2] |
| *Brevundimonas vesicularis* DC263 | CrtG, CrtI, CrtW, CrtY, CrtZ | *ABC50107*, *ABC50114*, *ABC50116*, *ABC50115*, *ABC50108* | 2,2’-Dihydroxyastaxanthin, 2,2’-Dihydroxyadonixanthin | N/A | N/A | [3] |
| *Caulobacter* sp. K31 | CrtB, CrtG, CrtI, CrtY, CrtZ | CaulDRAFT_3785, CaulDRAFT_4828, CaulDRAFT_3784, CaulDRAFT_3783, CaulDRAFT_3786 | Caloxanthin/Nostoxanthin | N | Y |  |
| **Parvularculales** | | | | | | |
| *Parvularcula bermudensis* HTCC2503 | CrtB, CrtI, CrtW, CrtY, CrtZ | PB2503_11014, PB2503_11019, PB2503_11009, PB2503_11024, PB2503_11004 | Astaxanthin | N | N |  |
| **Rhizobiales - Aurantimonadaceae** | | | | | | |
| *Aurantimonas* sp. SI85-9A1 | CrtB, CrtI, CrtW, CrtX, CrtY, CrtZ | SI859A1_00462, SI859A1_00461, SI859A1_00535, SI859A1_00459, SI859A1_00460, SI859A1_00536 | Astaxanthin glycoside | N | N |  |
| *Fulvimarina pelagi* HTCC2506 | CrtB, CrtC, CrtD, CrtF, CrtI, CrtW, CrtX, CrtY, CrtZ | FP2506_14039, FP2506_11697, FP2506_11702, FP2506_11707, FP2506_14044, FP2506_12624, FP2506_12634, FP2506_14049, FP2506_12644 | Astaxanthin glycoside, Spirilloxanthin | Y | N |  |
| **Rhizobiales - Bradyrhizobiaceae** | | | | | | |
| *Bradyrhizobium* sp. BTAi1 | CrtB, CrtC, CrtD, CrtF, CrtI | BBta_6444, BBta_6442, BBta_6441, BBta_6439, BBta_6445 | Spirilloxanthin | N | Y | [4] |
| *Bradyrhizobium* sp. ORS278 | CrtB (2x), CrtC, CrtD, CrtF, CrtI (2x), CrtW, CrtY | BRADO1611, *AAF78202*, *AAR98493*, *AAR98494*, BRADO1616, *AAR98491*, *AF218415*, *AAF78203*, *AAF78200* | Canthaxanthin, Spirilloxanthin | N | Y | [4-6] |
| *Rhodopseudomonas palustris* BisA53 | CrtB, CrtC, CrtD, CrtF, CrtI | RPE_1316, RPE_1320, RPE_1323, RPE_1325, RPE_1315 | Spirilloxanthin | N | N |  |
| *Rhodopseudomonas palustris* BisB18 | CrtB, CrtC, CrtD, CrtF, CrtI | RPC_1262, RPC_1288, RPC_1289, RPC_1291, RPC_1261 | Spirilloxanthin | N | N |  |
| *Rhodopseudomonas palustris* BisB5 | CrtB, CrtC, CrtD, CrtF, CrtI | RPD_3765, RPD_3761, RPD_3760, RPD_3758, RPD_3766 | Spirilloxanthin | N | N |  |
| *Rhodopseudomonas palustris* CGA009 | CrtB, CrtC, CrtD, CrtF, CrtI | RPA1513, RPA1517, RPA1518, RPA1520, RPA1512 | Spirilloxanthin | N | N |  |
| *Rhodopseudomonas palustris* HaA2 | CrtB, CrtC, CrtD, CrtF, CrtI | RPB_4010, RPB_4006, RPB_4005, RPB_4003, RPB_4011 | Spirilloxanthin | N | N |  |
|  |  |  |  |  |  |  |
| *Rhodopseudomonas palustris* TIE-1 | CrtB, CrtC, CrtD, CrtF, CrtI | *ZP_02303360*, *ZP_02303365*, *YP_001990709*, *ZP_02303368*, *ZP_02303359* | Spirilloxanthin | N | N |  |
| **Rhizobiales - Methylobacteriaceae** | | | | | | |
| *Methylobacterium* sp. 4-46 | CrtB, CrtC, CrtD, CrtF, CrtI | M446DRAFT_2531, M446DRAFT_2575, M446DRAFT_2574, M446DRAFT_2572, M446DRAFT_2532 | Spirilloxanthin | Y | Y |  |
| *Methylobacterium* *chloromethanicum* CM4 | CrtB, CrtC, CrtD, CrtF, CrtI | MchlDRAFT_3516, MchlDRAFT_3862, MchlDRAFT_3773, MchlDRAFT_3771, MchlDRAFT_3517 | Spirilloxanthin | N | N |  |
| *Methylobacterium extorquens* AM1 | CrtI | *AAQ65246* | ? | N/A | N/A | [7] |
| *Methylobacterium extorquens* PA1 | CrtB, CrtC, CrtD, CrtF, CrtI | MextDRAFT_0800, MextDRAFT_3418, MextDRAFT_2857, MextDRAFT_2859, MextDRAFT_0801 | Spirilloxanthin | N | Y |  |
| *Methylobacterium populi* BJ001 | CrtB, CrtC, CrtD, CrtF, CrtI | *ZP_02198499*, *ZP_02200220*, *ZP_02200038*, *ZP_02200040*, *ZP_02198498* | Spirilloxanthin | N | N |  |
| **Rhizobiales - Phyllobacteriaceae** | | | | | | |
| *Hoeflea phototrophica* DFL-43 | CrtA(P), CrtB, CrtC, CrtD, CrtF, CrtI | *ZP_02167527,* *ZP_02167509*, *ZP_02167511*, *ZP_02167512*, *ZP_02167515*, *ZP_02167508* | Spirilloxanthin/2,2’-Diketosprilloxanthin, Spheroidene/Spheroidenone | N | N |  |
| **Rhizobiales - Xanthobacteraceae** | | | | | | |
| *Xanthobacter autotrophicus* Py2 | CrtB, CrtI, CrtX, CrtY, CrtZ | *AAL02001*, *AAL02000*, Xaut_3579, *AAL01999*, Xaut_4535 | Zeaxanthin diglucoside | N | Y | [8] |
| **Rhodobacterales - Rhodobacteraceae** | | | | | | |
| α-Proteobacterium HTCC2255 | CrtB (2x), CrtI (2x), CrtY | OM2255_09541, OM2255_14570, OM2255_09536, OM2255_14565, OM2255_09546 | β-Carotene | Y | N |  |
| *Dinoroseobacter shibae* DFL 12 | CrtA(R), CrtB, CrtC, CrtD, CrtF, CrtI | DshiDRAFT_2007, DshiDRAFT_2014, DshiDRAFT_2502, DshiDRAFT_2017, DshiDRAFT_2019, DshiDRAFT_2013 | Spheroidene/Spheroidenone | N | Y | [9] |
| *Jannaschia* sp. CCS1 | CrtA(R), CrtB, CrtC, CrtD, CrtF, CrtI | Jann_0145, Jann_0142, Jann_0184, Jann_0183, Jann_0181, Jann_0143 | Spheroidene/Spheroidenone | N | Y |  |
| *Loktanella vestfoldensis* SKA53 | CrtA(R), CrtB, CrtC, CrtD, CrtF, CrtI | SKA53_12108, SKA53_12123, SKA53_12308, SKA53_12313, SKA53_12323, SKA53_12118 | Spheroidene/Spheroidenone | N | N |  |
| *Paracoccus haeundaensis* | CrtB, CrtI, CrtW, CrtY, CrtZ | *AAY28421*, *AAY28420*, *AAY28417*, *AAY28419*, *AAY28418* | Astaxanthin | N/A | N/A | [10,11] |
| *Paracoccus* sp. N81106 | CrtB, CrtI, CrtW, CrtY, CrtZ | *P54975*, *BAA09594*, *BAA09591*, *BAA09593*, *BAA09592* | Astaxanthin | N/A | N/A | [12,13] |
| *Paracoccus* sp. PC1 | CrtW, CrtZ | *Q44261*, *Q44262* | Astaxanthin | N/A | N/A | [12,13] |
| *Paracoccus zeaxanthinifaciens* R1534 | CrtB, CrtI, CrtY, CrtZ | *AAC44849*, *AAC44850*, *AAC44851*, *AAC44852* | Zeaxanthin | N/A | N/A | [14,15] |
| *Rhodobacter capsulatus* | CrtA(R), CrtB, CrtC, CrtD, CrtF, CrtI | *1613414A*, *1613414C*, *1613414E*, *1613414F*, *1613414H*, *1613414B* | Spheroidene/Spheroidenone | N | N | [4,16] |
| *Rhodobacter sphaeroides* 2.4.1 | CrtA(R), CrtB, CrtC, CrtD, CrtF, CrtI | 640071482, *AAB31139*, *AAF24292*, *AAF24293*, *AAF24295*, *AAF24289* | Spheroidene, Spheroidenone | N | Y | [4,17,18] |
|  |  |  |  |  |  |  |
|  |  |  |  |  |  |  |
| *Rhodobacter sphaeroides* ATCC 17025 | CrtA(R), CrtB, CrtC, CrtD, CrtF, CrtI | Rsph17025_1022, Rsph17025_1025, Rsph17025_2041, Rsph17025_2040, Rsph17025_2038, Rsph17025_1024 | Spheroidene/Spheroidenone | N | Y |  |
| *Roseobacter denitrificans* OCh 114 | CrtB, CrtC, CrtD, CrtF, CrtI | RD1_0119, RD1_0116, RD1_0115, RD1_0113, RD1_0120 | Spirilloxanthin/2,2’-Diketoxanthin, Spheroidene/Spheroidene | N | Y | [4] |
| *Roseobacter litoralis* OCh 149 | CrtA(R), CrtB, CrtC, CrtD, CrtF, CrtI | *ZP_02142877*, *ZP_02142880*, *ZP_02142883*, *ZP_02142884*, *ZP_02142886*, *ZP_02142879* | Spheroidene/Spheroidenone | N | N | [4] |
| *Roseobacter* sp. AzwK-3b | CrtA(R), CrtB, CrtC, CrtD, CrtF, CrtI | RAZWK3B_19701, RAZWK3B_19686, RAZWK3B_19676, RAZWK3B_19671, RAZWK3B_19661, RAZWK3B_19691 | Spheroidene/Spheroidenone | N | N |  |
| *Roseobacter* sp. CCS2 | CrtA(R), CrtB, CrtC, CrtD, CrtF, CrtI | RCCS2_09379, RCCS2_09364, RCCS2_08389, RCCS2_08384, RCCS2_08374, RCCS2_09369 | Spheroidene/Spheroidenone | N | N |  |
| *Roseovarius* sp. 217 | CrtA(R), CrtB, CrtC, CrtD, CrtF, CrtI | ROS217_22377, ROS217_22362, ROS217_22352, ROS217_22347, ROS217_22337, ROS217_22367 | Spheroidene/Spheroidenone | N | N |  |
| *Roseovarius* sp. TM1035 | CrtA(R), CrtB, CrtC, CrtD, CrtF, CrtI | RTM1035_06838, RTM1035_06853, RTM1035_12198, RTM1035_12193, RTM1035_12183, RTM1035_06848 | Spheroidene/Spheroidenone | N | N |  |
| **Rhodobacterales - Rhodospirillaceae** | | | | | | |
| *Magnetospirillum magnetotacticum* MS-1 | CrtB, CrtD, CrtI, CrtI | Magn03002575, Magn03004919, Magn03002576, Magn03006152 | ? | Y? | N |  |
| *Rhodospirillum rubrum* ATCC 11170 | CrtB, CrtC, CrtD, CrtF, CrtI | Rru_A0494, Rru_A2985, Rru_A2984, Rru_A2982, Rru_A0493 | Spirilloxanthin | N | Y | [4] |
| ***Rickettsiales*** | | | | | | |
| Candidatus *Pelagibacter ubique* HTCC1002 | CrtB, CrtI, CrtY | *ZP_01264713*, PU1002_05751, PU1002_05741 | β-Carotene | Y | N |  |
|  |  |  |  |  |  |  |
| Candidatus *Pelagibacter ubique* HTCC1062 | CrtB, CrtI, CrtY | *YP_265548*, SAR11_0120, *YP_265549* | β-Carotene^c^ | Y | Y | [19] |
| **Sphingomonadales** | | | | | | |
| α-Proteobacterium BAL199 | CrtI, CrtY | *ZP_02189377*, *ZP_02189374* | β-Carotene | Y | N |  |
| *Erythrobacter litoralis* HTCC2594 | CrtB, CrtG, CrtI, CrtW, CrtY, CrtZ | ELI_09895, ELI_12610, ELI_09885, ELI_03320, ELI_09880, ELI_03325 | Erythroxanthin sulphate | N | Y |  |
| *Erythrobacter longus* OCH101 | CrtI, CrtY | *BAA20276*, *BAA20275* | β -Carotene, Caloxanthin, Erythroxanthin sulphate, Spirilloxanthin, Zeaxanthin | N/A | N/A | [4,20] |
| *Erythrobacter* sp. NAP1 | CrtB, CrtC, CrtD, CrtF, CrtG, CrtI, CrtW, CrtY, CrtZ | NAP1_10293, NAP1_09287, NAP1_09292, NAP1_09297, NAP1_13433, NAP1_10278, NAP1_09087, NAP1_10273, NAP1_09082 | Erythroxanthin sulphate, Spirilloxanthin |  | N | [21] |
| *Erythrobacter* sp. SD-21 | CrtB, CrtG, CrtI, CrtY, CrtZ | ED21_21989, ED21_19052, ED21_22004, ED21_22009, ED21_23836 | Caloxanthin/Nostoxanthin | N | N |  |
| *Novosphingobium aromaticivorans* DSM 12444 | CrtB, CrtG, CrtI, CrtY, CrtZ | Saro_1814, Saro_0236, Saro_1816, Saro_1817, Saro_1168 | Caloxanthin/Nostoxanthin | N | Y |  |
| *Sphingomonas* sp. SKA58 | CrtB, CrtG, CrtI, CrtY, CrtZ | SKA58_15537, SKA58_00155, SKA58_15527, SKA58_15522, SKA58_17557 | Caloxanthin/Nostoxanthin | N | N |  |
| *Sphingopyxis alaskensis* RB2256 | CrtB, CrtG, CrtI, CrtY, CrtZ | Sala_3132, Sala_3136, Sala_3134, Sala_3135, Sala_2128 | Caloxanthin/Nostoxanthin | N | Y |  |
| **Other α-Proteobacteria** | | | | | | |
| *Methylomonas* sp. 16a | CrtN, CrtNb, Ald | *AAX46183*, *AAX46185*, *AAX46184* | 4,4’-Diapolycopene-4-oic acid,  4,4’-Diapolycopene-4,4’-dioic acid | N/A | N/A | [22] |
| *Methylophilales* Bacterium HTCC2181 | CrtB, CrtI, CrtY | MB2181_00930, MB2181_00935, *ZP_01551534* | β-Carotene | Y | N |  |
| *Thiocapsa roseopersicina* | CrtC, CrtD, CrtF | *AAP50935*, *AAP59036*, *AAP59038* | Spirilloxanthin | N/A | N/A | [4,23] |
| **β-Proteobacteria** | | | | | | |
| *Rubrivivax gelatinosus* S1 | CrtA(P), CrtB, CrtC, CrtD, CrtF, CrtI | *AAO93123*, *AAB87738*, *AAO93124*, *AAC44798*, *AAO93114*, *AAO93135* | Spirilloxanthin/2,2’-Diketoxanthin, Spheroidene/Spheroidenone | N/A | N/A | [4,24-32] |
| Uncultured Marine Bacterium EB0_41B09 | CrtB, CrtI, CrtY | *ABL97760*, *ABL97761*, *ABL97759* | β -Carotene | Y | N/A |  |
|  | | | | | | |
| **γ-Proteobacteria** | | | | | | |
| *Congregibacter litoralis* KT71 | CrtB, CrtC, CrtD, CrtF, CrtI | KT71_19483, KT71_19478, KT71_07854, KT71_19468, KT71_19488 | Spirilloxanthin | N | N | [33] |
| *Cronobacter sakazakii* ATCC BAA-894 | CrtB, CrtI, CrtX, CrtY, CrtZ | ESA_00342, ESA_00343, ESA_00345, ESA_00344, ESA_00341 | Zeaxanthin glucoside | N/A | N/A | [34] |
| *Enterobacteriaceae* Bacterium DC260 | CrtB, CrtI, CrtX, CrtY, CrtZ | *AAZ73131*, *AAZ73130*, *AAZ73128*, *AAZ73129*, *AAZ73132* | Zeaxanthin glucoside | N/A | N/A | [35] |
| *Enterobacteriaceae* Bacterium DC404 | CrtB, CrtI, CrtY, CrtZ | *AAZ73137*, *AAZ73136*, *AAZ73135*, *AAZ73138* | Zeaxanthin glucoside | N/A | N/A | [35] |
| *Enterobacteriaceae* Bacterium DC413 | CrtB, CrtI, CrtX, CrtY, CrtZ | *AAZ73150*, *AAZ73149*, *AAZ73147*, *AAZ73148*, *AAZ73151* | Zeaxanthin glucoside | N/A | N/A | [35] |
| *Enterobacteriaceae* Bacterium DC416 | CrtB, CrtI, CrtX, CrtY, CrtZ | *AAZ73143*, *AAZ73142*, *AAZ73140*, *AAZ73141*, *AAZ73144* | Zeaxanthin glucoside | N/A | N/A | [35] |
| *Halorhodospira halophila* SL1 | CrtB, CrtC, CrtD, CrtF, CrtI | Hhal_1618, Hhal_1615, Hhal_1614, Hhal_1612, Hhal_1619 | Spirilloxanthin | N | Y | [4] |
| Marine γ-Proteobacterium HTCC2080 | CrtB, CrtC, CrtD, CrtF, CrtI | MGP2080_10278, MGP2080_10273, MGP2080_10308, MGP2080_10263, MGP2080_10283 | Spirilloxanthin | N | N |  |
| Marine γ-Proteobacterium HTCC2143 | CrtB, CrtI, CrtY | GP2143_03303, GP2143_03298, GP2143_03308 | β-Carotene | Y | N |  |
| Marine γ-Proteobacterium HTCC2207 | CrtB, CrtI, CrtY | GB2207_10296, GB2207_10301, GB2207_10291 | β-Carotene | Y | N |  |
|  |  |  |  |  |  |  |
| *Marinobacter* sp. ELB17 | CrtB, CrtI, CrtY | MELB17_06699, MELB17_06694, MELB17_06704 | β-Carotene | Y | Y |  |
| *Pantoea agglomerans* Eho10 | CrtB, CrtI, CrtX, CrtY, CrtZ | *AAA21264*, *AAA21263*, *AAA64979*, *AAA21262*, *AAA64983* | Zeaxanthin glucoside | N/A | N/A | [36,37] |
| *Pantoea ananatis* ATCC 19321 | CrtB, CrtI, CrtX, CrtY, CrtZ | *P21683*, *BAA14127*, *BAA14125*, *BAA14126*, *BAA14129* | Zeaxanthin glucoside | N/A | N/A | [38] |
| *Photobacterium* sp. SKA34 | CrtB, CrtI, CrtY | SKA34_07099, SKA34_07104, SKA34_07094 | β-Carotene | Y | N |  |
| *Photorhabdus luminescens* subsp. *laumondii* TTO1 | CrtB, CrtI, CrtY | plu4343, plu4342, plu4341 | β-Carotene | N | Y |  |
|  |  |  |  |  |  |  |
| *Pseudomonas stutzeri* A1501 | CrtB, CrtI, CrtX, CrtY, CrtZ | PST_3872, PST_3873, PST_3875, PST_3874, PST_3871 | Zeaxanthin glucoside | N | N |  |
| *Vibrio angustum* S14 | CrtB, CrtI, CrtY | VAS14_08785, VAS14_08780, VAS14_08790 | β-Carotene | Y | N |  |
| *Vibrio campbellii* AND4 | CrtB, CrtI, CrtY | *ZP_02194908*, *ZP_02194909*, *ZP_02194907* | β-Carotene | Y | Y |  |
| *Vibrio harveyi* ATCC BAA-1116 | CrtB, CrtI, CrtY | VIBHAR_02163, VIBHAR_02162, *YP_001445356* | β-Carotene | Y | N |  |
| **δ-Proteobacteria** | | | | | | |
| *Bdellovibrio bacteriovorus* HD100 | CrtB, CrtG, CrtI, CrtY | Bd1725, Bd1729, Bd1724, Bd1730 | Xanthophyll | N | Y |  |
| **Myxobacteria** | | | | | | |
| *Myxococcus xanthus* DK 1622^d^ | CrtB, CrtC, CrtD, CrtI (3x) CrtW, CrtYc, CrtYd | MXAN_0896, MXAN_0898, MXAN_0897, MXAN_0895, MXAN_4052, MXAN_7517, MXAN_6049, *YP_629162*, *YP_629161* | 4-Ketotorulene | N | Y | [39-41] |
| *Plesiocystis pacifica* SIR-1 | CrtB, CrtC, CrtD, CrtI, CrtL, CrtU | PPSIR1_32939, PPSIR1_30569, PPSIR1_30564, PPSIR1_32944, PPSIR1_19284, PPSIR1_36107 | Chlorobactene-like? | N | N |  |
| *Sorangium cellulosum* | CrtB, CrtC, CrtD, CrtI, CrtZ, CruA | *YP_001611218*, *YP_001611221*, *YP_001611222*, *YP_001611217*, *YP_001611220*, *YP_001611219* | Myxol | N | Y |  |
| *Stigmatella aurantiaca* DW4/3-1 | CrtB, CrtC, CrtD, CrtI (2x), CrtYcd | STIAU_8492, STIAU_8490, STIAU_8491, STIAU_8493, STIAU_0585, ZP_01463227 | Myxol-like | N | N |  |
| **Unclassified Proteobacteria** | | | | | | |
| Uncultured Marine Bacterium HF10_19P19 | CrtB, CrtI, CrtY | *ABL60985*, *ABL60986*, *ABL60984* | β-Carotene | Y | N |  |
| Uncultured Marine Bacterium HF10_25F10 | CrtB, CrtI, CrtY | *ABL61010*, *ABL61009*, *ABL61011* | β-Carotene | Y | N |  |
| **Bacteroidetes** | | | | | | |
| Unidentified Eubacterium SCB49 | CrtB, CrtI, CrtYcd, CrtZ | SCB49_01402, SCB49_01397, SCB49_01412, SCB49_01407 | Zeaxanthin | N | N |  |
| ***Flavobacteria*** | | | | | | |
| *Cellulophaga* sp. MED134 | CrtB, CrtI, CrtY, CrtZ | MED134_13071, MED134_13076, MED134_08681, MED134_13066 | Zeaxanthin^c^ | Y | N | [42] |
| *Croceibacter atlanticus* HTCC2559 | CrtB, CrtI, CrtY, CrtZ | CA2559_00920, CA2559_00915, CA2559_00930, CA2559_00925 | Zeaxanthin | N | N |  |
| Flavobacteria Bacterium BAL38 | CrtB, CrtI, CrtY, CrtZ | FBBAL38_01010, FBBAL38_01005, FBBAL38_05810, FBBAL38_01015 | Zeaxanthin | Y | N |  |
| Flavobacteria Bacterium BBFL7 | CrtA(P), CrtB, CrtD, CrtI, CrtY, CrtZ | BBFL7_00795, BBFL7_00790, BBFL7_00792, BBFL7_00789, BBFL7_00794, BBFL7_00791 | Myxol | N | N |  |
|  |  |  |  |  |  |  |
| *Flavobacteriales* Bacterium ALC-1 | CrtB, CrtI, CrtYcd, CrtZ | *ZP_02183100*, *ZP_02183098*, *ZP_02183102*, *ZP_02183101* | Zeaxanthin | N | N |  |
| *Flavobacteriales* Bacterium HTCC2170 | CrtB, CrtI, CrtYcd, CrtZ | FB2170_07629, FB2170_07624, FB2170_07639, FB2170_07634 | Zeaxanthin | N | N |  |
| *Flavobacterium johnsoniae* UW101 | CrtB, CrtI, CrtY, CrtZ | Fjoh_0058, Fjoh_0057, Fjoh_0926, Fjoh_0059 | Zeaxanthin | N | Y |  |
| *Flavobacterium psychrophilum* JIP02/86 | CrtB, CrtI, CrtY, CrtZ | FP1450, FP1449, FP1447, FP1451 | Zeaxanthin | N | Y |  |
| *Gramella forsetii* KT0803 | CrtB, CrtI, CrtY, CrtZ | orf2472, orf2471, orf2474, orf2473 | Zeaxanthin | N | Y |  |
| *Kordia algicida* OT-1 | CrtB, CrtI, CrtY, CrtZ | *ZP_02163393*, *ZP_02163392*, *ZP_02163354*, *ZP_02163394* | Zeaxanthin | N | N |  |
| *Leeuwenhoekiella blandensis* MED217 | CrtB, CrtI, CrtY, CrtZ | MED217_11644, MED217_11639, MED217_17665, MED217_11649 | Zeaxanthin | N | N |  |
| Marine Bacterium P99-3 | CrtA(P), CrtD, CrtF, CrtI, CrtL, CrtZ | *BAC77674*, *BAC77671*, *AAR98496*, *BAC77668*, *BAC77673*, *BAC77670* | Myxol | N/A | N/A | [43-45] |
| *Polaribacter irgensii* 23-P | CrtA(P), CrtB, CrtD, CrtI, CrtY, CrtZ | PI23P_00055, PI23P_12132, PI23P_11812, PI23P_12137, PI23P_04492, PI23P_11807 | Myxol | Y | N |  |
| *Psychroflexus torquis* ATCC 700755 | CrtA(P), CrtB (2x), CrtD, CrtI, CrtY (2x), CrtYcd (2x) | P700755_03257, P700755_03232, P700755_26055, P700755_03242, P700755_03227, P700755_03252, P700755_07212, P700755_26050, P700755_32979 | Myxol | Y | N |  |
| *Robiginitalea biformata* HTCC2501 | CrtB, CrtI, CrtY, CrtZ | RB2501_11842, RB2501_11847, RB2501_05565, RB2501_11837 | Zeaxanthin | N | N |  |
| *Tenacibaculum* sp. MED152 | CrtA(P), CrtB, CrtD, CrtI, CrtY, CrtZ (2x) | *ZP_01052136*, MED152_02670, MED152_02575, MED152_02675, MED152_02660, MED152_02565, MED152_02665 | Myxol^c^ | Y | N | [42] |
| **Sphingobacteria** | | | | | | |
| *Algoriphagus* sp. KK10020C | CrtI, CrtW, CrtYcd | *AAB88949*, *ABB88952*, *ABB88950* | Flexixanthin | N/A | N/A | [46] |
| *Algoriphagus* sp. PR1 | CrtB, CrtD, CrtI, CrtW, CrtYcd, CrtZ | ALPR1_00745, ALPR1_03175, ALPR1_00750, ALPR1_00730, ALPR1_00740, ALPR1_00765 | Ketomyxol-like | N | N |  |
| *Cytophaga hutchinsonii* ATCC 33406 | CrtB, CrtI, CrtYcd, CrtZ | CHU_2036, CHU_2033, CHU_2039, CHU_2038 | Zeaxanthin | N | Y |  |
| *Microscilla marina* ATCC 23134 | CrtB, CrtD, CrtI, CrtYcd, CrtZ | M23134_07222, M23134_07225, M23134_07221, M23134_07228, M23134_07227 | Myxol-like | N | N |  |
| *Pedobacter* sp. BAL39 | CrtB, CrtI, CrtY, CrtZ | PBAL39_25495, PBAL39_25490, PBAL39_23642, PBAL39_25510 | Zeaxanthin | N | N |  |
| *Salinibacter ruber* DSM 13855 | CrtB, CrtI (2x), CrtO, CrtYcd | SRU_1430, SRU_0743, SRU_2060, SRU_1502, YP_445624 | Salinixanthin | Y | Y | [47,48] |
| **Firmicutes** | | | | | | |
| **Bacillales** | | | | | | |
| *Bacillus* sp. NRRL B-14911 | CrtM, CrtN (2x), CrtNb, CrtO(at), CrtQ(gt) | B14911_18170, B14911_18150, B14911_20973, B14911_18165, *ZP_01169745*, *ZP_01169743* | Staphyloxanthin | N | N |  |
| *Bacillus pumilus* SAFR-032 | CrtM, CrtN, CrtNb | *YP_001486256*, BPUM_1011, *YP_001487908* | 4,4’-Diapolycopene oxide/4,4’-Diaponeurosporene oxide | N | Y |  |
| *Bacillus selenitireducens* MLS10 | CrtM, CrtN, CrtNb | *ZP_02170848*, *ZP_02170846*, *ZP_02171655* | 4,4’-Diapolycopene oxide/4,4’-Diaponeurosporene oxide | N | N |  |
| *Oceanobacillus iheyensis* HTE831 | CrtM, CrtN, CrtNb, CrtO(at), CrtQ(gt) | OB2460, OB2461, OB2459, *NP_693378*, *NP_693379* | Staphyloxanthin | N | Y |  |
| *Exiguobacterium sibiricum* 255-15 | CrtM, CrtN, CrtNb | *ZP_00538724*, ExigDRAFT_1694, ExigDRAFT_2090 | 4,4’-Diapolycopene oxide/4,4’-Diaponeurosporene oxide | Y | Y |  |
| *Staphylococcus aureus aureus* Newman^e^ | CrtM, CrtN, CrtNb, CrtO(at), CrtQ(gt) | *CAA52097*, *CAA52098*, *Q2FV57*, NWMN_2465, *Q53590* | Staphyloxanthin | N | Y | [22,49,50] |
| *Staphylococcus haemolyticus* JCSC1435 | CrtM, CrtN, CrtNb, CrtO(at), CrtQ(gt) | SH0490, SH0491, SH0488, *YP_252402*, SH0489 | Staphyloxanthin | N | Y |  |
| **Lactobacillales** | | | | | | |
| *Carnobacterium* sp. AT7 | CrtM, CrtN, CrtNb, CrtO(at), CrtQ(gt) | *ZP_02184019*, *ZP_02184020*, *ZP_02184018*, *ZP_02184015*, *ZP_02184016* | Staphyloxanthin | N | N |  |
| *Lactobacillus plantarum* WCFS1 | CrtM, CrtN | lp_3263, lp_3262 | 4,4’-Diapolycopene/4,4’-Diaponeurosporene | N | Y |  |
| *Leuconostoc citreum* KM20 | CrtM, CrtN | *YP_001728065*, *YP_001728064* | 4,4’-Diapolycopene/4,4’-Diaponeurosporene | N | Y |  |
| *Leuconostoc mesenteroides* subsp. *mesenteroides* ATCC 8293 | CrtM, CrtN | LEUM_1047, LEUM_1046 | 4,4’-Diapolycopene/4,4’-Diaponeurosporene | N | Y |  |
| **Clostridiales - Heliobacteriaceae** | | | | | | |
| *Heliobacterium modesticaldum* Ice1 | CrtN | *YP_001679882* | 4,4 -Diaponeurosporene | N | ? | [51] |
| **Mollicutes - Acholeplasmatales** | | | | | | |
| *Acholeplasma laidlawii* PG-8A | CrtM, CrtN | *YP_001621382*, *YP_001621383* | 4,4’-Diapolycopene/4,4’-Diaponeurosporene | N | Y |  |
| **Actinobacteria** | | | | | | |
| Marine Actinobacterium PHSC20C1 | CrtB, CrtEb, CrtI, CrtYe, CrtYf | A20C1_09079, A20C1_09059, A20C1_09074, *ZP_01129023*, *ZP_01129022* | Decaprenoxanthin/C.P.450 | N | N |  |
| *Rubrobacter xylanophilus* DSM 9941 | CrtB, CrtI, CrtY (2x) | Rxyl_0844, Rxyl_0845, Rxyl_2038, YP_645780 | β-Carotene | Y | Y |  |
|  | | | | | | |
| **Corynebacterineae** | | | | | | |
| *Corynebacterium diphtheriae* NCTC 13129 | CrtB, CrtI | DIP1870, DIP1871 | ? | N | Y |  |
| *Corynebacterium efficiens* YS-314 | CrtB, CrtEb, CrtI, CrtYe, CrtYf | CE0641, CE0637, CE0640, CE0638, CE0639 | Decaprenoxanthin | N | Y |  |
| *Corynebacterium glutamicum* ATCC 13032^f^ | CrtB (2x), CrtEb, CrtI (2x, one broken), CrtYe, CrtYf | cg0721, cg2672, cg0717, cg0720, cg2668+cg2670 (fused), cg0718, cg0719 | Decaprenoxanthin | N | Y | [52] |
| *Corynebacterium jeikeium* K411 | CrtB, CrtI | jk0515, jk0735 | ? | N | Y |  |
| *Dietzia* sp. CQ4 | CrtB, CrtEbYe (LitAB), CrtI, CrtL, CrtYf (LitC) | *ABD24399*, *ABD24402*, *ABD24400*, *ABD24404*, *ABD24401* | Canthaxanthin, C.P.450 | N/A | N/A | [53] |
| *Gordonia* sp. TM414 | CrtB, CrtI | *BAC75675*, *BAC75676* | ? | N/A | N/A | [54] |
| *Mycobacterium aurum* A+ | CrtB, CrtI, CrtU, CrtYc, CrtYd | *CAB94795*, *CAB94794*, *CAB94798*, *CAB94797*, *CAB94796* | Isorenieratene | N/A | N/A | [55] |
| *Mycobacterium avium* subsp. *paratuberculosis* K-10^h^ | CrtB, CrtI, CrtU, CrtYc, CrtYd | MAP3071, MAP3070, MAP3075, MAP3072, MAP3073 | Isorenieratene | N | Y |  |
| *Mycobacterium gilvum* PYR-GCK | CrtB, CrtI, CrtL, CrtU | Mflv_1846, Mflv_1847, Mflv_0950, Mflv_1844 | Isorenieratene | N | Y |  |
| *Mycobacterium smegmatis* MC2 155 | CrtB, CrtI, CrtU | MSMEG_2346, MSMEG_2347, MSMEG_2344 | Isorenieratene? | N | Y |  |
| *Mycobacterium* sp. MCS^g^ | CrtB, CrtI, CrtU, CrtYc, CrtYd | Mmcs_5076, Mmcs_5075, Mmcs_5080, Mmcs_5077, Mmcs_5078 | Isorenieratene | N | Y |  |
| *Mycobacterium ulcerans* Agy99 | CrtB, CrtYc, CrtYd | MUL_0375, MUL_0376, MUL_0377 | ? | N | Y |  |
| *Mycobacterium vanbaalenii* PYR-1 | CrtB, CrtI, CrtL, CrtU | Mvan_1579, Mvan_1578, Mvan_5914, Mvan_1580 | Isorenieratene | N | Y |  |
|  |  |  |  |  |  |  |
| *Nocardia farcinica* IFM 10152 | CrtB (2x), CrtI (2x), CrtL (2x), CrtO | nfa17350, nfa43980, nfa17370, nfa43990, nfa7290, nfa34900, nfa17530 | Canthaxanthin | N | Y |  |
| *Rhodococcus erythropolis* AN12 | CrtI, CrtL, CrtO | *AAW23161*, *AAR98749*, *AAW23159* | 4-Keto-γ-carotene | N/A | N/A | [56,57] |
| *Rhodococcus* sp. RHA1 | CrtB, CrtI, CrtL, CrtO | RHA1_ro01109, RHA1_ro01107, RHA1_ro07203, RHA1_ro01101 | 4-Keto-γ-carotene | N | Y |  |
| **Frankineae** | | | | | | |
| *Frankia alni* ACN14a | CrtB, CrtI, CrtYc, CrtYd | FRAAL2154, FRAAL2160, *YP_715252*, *YP_715253* | β-Carotene | N | Y |  |
| *Frankia* sp. CcI3 | CrtB, CrtI, CrtYc, CrtYd | Francci3_1383, Francci3_1387, *YP_482155*, *YP_482154* | β-Carotene | N | Y |  |
| *Frankia* sp. EAN1pec | CrtB, CrtI, CrtYc, CrtYd | Franean1DRAFT_1529, Franean1DRAFT_1533, *YP_001506187*, *YP_001506188* | β-Carotene | N | Y |  |
| *Kineococcus radiotolerans* SRS30216 | CrtB, CrtI, CrtY | Krad_3229, Krad_3228, Krad_0091 | β-Carotene | Y | Y |  |
|  | | | | | | |
| **Micrococcineae** | | | | | | |
| *Brevibacterium linens* ATCC 9175 | CrtB, CrtI, CrtU, CrtYc, CrtYd, ORF10 | *AAF65581*, *AAF65582*, *AAF65586*, *AAF65588*, *AAF65587* , *AAF65589* | 3,3’-Dihydroxyisorenieratine | N/A | N/A | [58-60] |
| *Brevibacterium linens* BL2 | CrtB, CrtI, CrtU, CrtYc, CrtYd, ORF10 | BlinB01002637, BlinB01002636, BlinB01002631, *ZP_00378971*, *ZP_00378970*, *ZP_00378969* | 3,3’-Dihydroxyisorenieratine | N | N |  |
| *Clavibacter michiganensis* subsp. *michiganensis* NCPPB 382^i^ | CrtB, CrtEb, CrtI, CrtYe, CrtYf | CMM_2887, CMM_2884, CMM_2887, *YP_001223630*, *YP_001223631* | Decaprenoxanthin/C.P.450 | N | Y |  |
| *Leifsonia xyli* subsp. *xyli* CTCB07 | CrtB, CrtEb, CrtI, CrtYe, CrtYf | Lxx15620, Lxx15580, Lxx15610, *YP_062469*, *YP_062470* | Decaprenoxanthin/C.P.450 | N | Y |  |
| *Arthrobacter aurescens* TC1 | CrtB, CrtEb, CrtI | AAur_0319, AAur_0315, AAur_0318 | Linear C50 carotenoids | N | Y |  |
| **Micromonosporineae** | | | | | | |
| *Salinispora arenicola* CNS205 | CrtB, CrtI, CrtU, CrtY | SareDRAFT_4686, SareDRAFT_4684, SareDRAFT_0271, SareDRAFT_2473 | Isorenieratene | N | Y |  |
| *Salinispora tropica* CNB-440 | CrtB, CrtI, CrtU, CrtY | Strop_4441, Strop_4439, Strop_0241, Strop_2408 | Isorenieratene | N | Y |  |
| **Pseudonocardineae** | | | | | | |
| *Saccharopolyspora erythraea* NRRL 2338 | CrtB (2x), CrtI, CrtL, CrtU | SACE_3269, SACE_3539, SACE_1713, SACE_2184, SACE_3271 | Isorenieratene | N | Y |  |
|  | | | | | | |
|  | | | | | | |
| **Streptomycineae** | | | | | | |
| *Streptomyces avermitilis* MA-4680 | CrtB, CrtI, CrtU, CrtY | SAV1024, SAV1023, SAV1019, SAV1021 | Isorenieratene | N | Y |  |
| *Streptomyces coelicolor* A3(2) | CrtB, CrtI, CrtU, CrtY | SCO0187, NP_639818, SCO0186, SCO0189, SCO0191 | Isorenieratene | N | Y |  |
| *Streptomyces griseus* NCBI 3933 | CrtB, CrtI, CrtU, CrtY | *P54977*, *CAA64850*, *CAA64853*, *CAA64855* | Isorenieratene | N/A | N/A | [61] |
| *Streptomyces rochei* 7434AN4 plasmid pSLA2-L | CrtB, CrtI, CrtU, CrtY | pSLA2-L_p105, pSLA2-L_p106, pSLA2-L_p110, pSLA2-L_p108 | Isorenieratene | N/A | N/A |  |
| **Streptosporangineae** | | | | | | |
| *Thermobifida fusca* YX | CrtB, CrtI, CrtU, CrtY | Tfu_3076, Tfu_3075, Tfu_3090, Tfu_3088 | Isorenieratene | N | Y |  |
| **Cyanobacteria** | | | | | | |
| **Chroococcales** | | | | | | |
| *Crocosphaera watsonii* WH 8501 | CrtB, CrtH, CrtP, CrtQ, CrtR, CruA, CruE, CruF, CruG, CruH, CruP | CwatDRAFT_0948, CwatDRAFT_6111, CwatDRAFT_4423, CwatDRAFT_0947, CwatDRAFT_5404, CwatDRAFT_5424, CwatDRAFT_6211, CwatDRAFT_3579, CwatDRAFT_6133, CwatDRAFT_6134, CwatDRAFT_4939, CwatDRAFT_1341 | β-Carotene, Myxol-like, Synechoxanthin, Zeaxanthin/Cryptoxanthin | N | N |  |
| *Cyanothece* sp. CCY 0110 | CrtB, CrtG, CrtH, CrtO, CrtP, CrtQ, CrtR, CruA, CrtE, CrtF, CrtG, CrtH, CruP | CY0110_11242, CY0110_22577, CY0110_09580, CY0110_24336, CY0110_15365, Cy0110_11237, CY0110_26552, CY0110_08481, CY0110_10722, CY0110_29874, CY0110_00310, CY0110_00315, CY0110_21165, CY0110_12337 | β-Carotene, Caloxanthin/Nostoxanthin, Echinenone/Canthaxanthin, Myxol-like, Synechoxanthin, Zeaxanthin/Cryptoxanthin | Y | N |  |
| *Microcystis aeruginosa* NIES-843 | CrtB, CrtH, CrtO, CrtP, CrtQ, CrtR, CrtX, CruA, CruF, CruG, CruP | *YP_001656306*, *YP_001656250*, *YP_001660920*, *YP_001656307*, *YP_001660324*, *YP_001655751*, *YP_001655539*, *YP_001655787*, *YP_001661102*, *YP_001661103*, *YP_001660458* | β-Carotene, Echinenone/Canthaxanthin, Myxol-like, Zeaxanthin/Cryptoxanthin | N | Y |  |
| *Synechococcus* sp. BL107 | CrtB, CrtH, CrtL, CrtP, CrtQ, CrtR, CrtW | BL107_05144, BL107_11616, BL107_15440, BL107_05149, BL107_04944, BL107_08054, BL107_14110 | β-Carotene, Echinenone/Canthaxanthin, Zeaxanthin/Cryptoxanthin | N | N |  |
| *Synechococcus* sp. CC9311 | CrtB, CrtH, CrtL, CrtP, CrtQ, CrtR, CrtW | sync_2607, sync_1140, sync_0974, sync_2608, sync_2568, sync_0336, sync_1804 | β-Carotene, Echinenone/Canthaxanthin, Zeaxanthin/Cryptoxanthin | N | Y |  |
| *Synechococcus* sp. CC9605 | CrtB, CrtH, CrtL, CrtP, CrtQ, CrtR | Syncc9605_2394, Syncc9605_1681, Syncc9605_1941, Syncc9605_2395, Syncc9605_2356, Syncc9605_0286 | β-Carotene, Zeaxanthin/Cryptoxanthin | N | Y |  |
|  |  |  |  |  |  |  |
|  |  |  |  |  |  |  |
| *Synechococcus* sp. CC9902 | CrtB, CrtH, CrtL, CrtP, CrtQ, CrtR, CrtW | Syncc9902_0299, Syncc9902_1423, Syncc9902_0724, Syncc9902_0298, Syncc9902_0335, Syncc9902_2058, Syncc9902_0972 | β-Carotene, Echinenone/Canthaxanthin, Zeaxanthin/Cryptoxanthin | N | Y |  |
| *Synechococcus* sp. JA-2-3Ba(2-13) | CrtB, CrtH, CrtP, CrtQ, CrtR, CruA, CruF, CruG, CruP | CYB_1695, CYB_1298, CYB_1694, CYB_1060, CYB_0102, CYB_0376, CYB_0539, CYB_0176, CYB_2530 | β-Carotene, Myxol-like, Zeaxanthin/Cryptoxanthin | N | Y |  |
| *Synechococcus* sp. JA-3-3Ab | CrtB, CrtH, CrtP, CrtQ, CrtR, CruA, CruF, CruG, CruP | CYA_0317, CYA_1857, CYA_0316, CYA_0668, CYA_1931, CYA_0185, CYA_1532, CYA_0955, CYA_1571 | β-Carotene, Myxol-like, Zeaxanthin/Cryptoxanthin | N | Y |  |
| *Synechococcus* sp. PCC 7002 | CrtB, CrtH, CrtP, CrtQ, CrtR, CrtW, CruA, CruE, CruF, CruG, CruH, CruP | *YP_001735179*, *YP_001735135*, *YP_001735178*, *YP_001733792*, *YP_001734175*, *YP_001736033*, *YP_001733316*, *YP_001734502*, *YP_001735274*, *YP_001735273*, *YP_001735480*, *YP_001735389* | β-Carotene, Caloxanthin/Nostoxanthin, Myxol-like, Synechoxanthin, Zeaxanthin/Cryptoxanthin | N | Y | [62-64] |
| *Synechococcus* sp. RCC307 | CrtB, CrtH, CrtL, CrtP, CrtQ, CrtR, CrtW | SynRCC307_0239, SynRCC307_1542, SynRCC307_0743, SynRCC307_0238, SynRCC307_0275, SynRCC307_2209, SynRCC307_1993 | β-Carotene, Echinenone/Canthaxanthin, Zeaxanthin/Cryptoxanthin | N | Y |  |
| *Synechococcus* sp. RS9916 | CrtB, CrtH, CrtL, CrtP, CrtQ, CrtR | RS9916_35877, RS9916_31147, RS9916_31637, RS9916_35882, RS9916_35677, RS9916_39311 | β-Carotene, Zeaxanthin/Cryptoxanthin | N | N |  |
| *Synechococcus* sp. RS9917 | CrtB, CrtG, CrtH, CrtL, CrtP, CrtQ, CrtR, CrtW | RS9917_07105, RS9917_09626, RS9917_00762, RS9917_01237, RS9917_07100, RS9917_07310, RS9917_03663, RS9917_00687 | β-Carotene, Caloxanthin/Nostoxanthin, Echinenone/Canthaxanthin, Zeaxanthin/Cryptoxanthin | N | N |  |
| *Synechococcus* sp. WH 5701 | CrtB, CrtG, CrtH, CrtL, CrtP, CrtQ, CrtR, CrtW | WH5701_00830, WH5701_02055, WH5701_09029, WH5701_08084, WH5701_00835, WH5701_00665, WH5701_01215, WH5701_04005 | β-Carotene, Caloxanthin/Nostoxanthin, Zeaxanthin/Cryptoxanthin | N | N |  |
| *Synechococcus* sp. WH 7803 | CrtB, CrtH, CrtL, CrtP, CrtQ, CrtR, CrtW | SynWH7803_2269, SynWH7803_1380, SynWH7803_1588, SynWH7803_2273, SynWH7803_2224, SynWH7803_0337, SynWH7803_0928 | β-Carotene, Echinenone/Canthaxanthin, Zeaxanthin/Cryptoxanthin | N | Y |  |
| *Synechococcus* sp. WH 7805 | CrtB, CrtH, CrtL, CrtP, CrtQ, CrtR, CrtW | WH7805_11093, WH7805_03627, WH7805_04751, WH7805_11088, WH7805_11278, WH7805_07481, WH7805_01197 | β-Carotene, Echinenone/Canthaxanthin, Zeaxanthin/Cryptoxanthin | N | N |  |
| *Synechococcus* sp. WH 8102 | CrtB, CrtH, CrtL, CrtP, CrtQ, CrtR, CrtW | SYNW2256, SYNW0901, SYNW0728, SYNW2257, SYNW2213, SYNW0291, SYNW1368 | β-Carotene, Echinenone/Canthaxanthin, Zeaxanthin/Cryptoxanthin | N | Y |  |
| *Synechococcus elongatus* PCC 7942^j^ | CrtB, CrtG, CrtH, CrtL, CrtP, CrtQ, CrtR, CruP | *P37269*, Synpcc7942_0680, Synpcc7942_1246, *CAA52677*, *CAA39004*, Synpcc7942_1512, Synpcc7942_2439, Synpcc7942_0652 | β-Carotene, Caloxanthin/Nostoxanthin, Zeaxanthin/Cryptoxanthin | N | Y | [63-67] |
|  |  |  |  |  |  |  |
|  |  |  |  |  |  |  |
| *Synechocystis* sp. PCC 6803 | CrtB, CrtD, CrtG, CrtH, CrtO, CrtP, CrtQ, CrtR, CrtX, CruA, CruE, CruF, CrtG, CruH, CruP | slr1255, *BAA16840*, slr0224, *BAA10798*, *BAA10561*, *CAA44452*, *P74306*, *BAA17468*, *NP_439972*, sll0659, sll0253, sll0814, sl11004, cbaB, sl0147 | β-Carotene, Caloxanthin/Nostoxanthin, Echinenone, Myxol, Synechoxanthin, Zeaxanthin/Cryptoxanthin | N | Y | [63,68-74] |
| *Thermosynechococcus elongatus* BP-1 | CrtB, CrtD, CrtG, CrtP, CrtQ, CrtR, CruA, CruF, CruG | tll1560, tll0232, tlr1917, tll1561, tll0337, tlr1900, tlr1139, tlr0414, tlr2019 | β-Carotene, Caloxanthin/Nostoxanthin, Echinenone/Canthaxanthin Hydroxymyxol 2’-fucoside, Myxol 2’-fucoside, Zeaxanthin/Cryptoxanthin | N | Y | [64,75] |
| **Gloeobacteria** | | | | | | |
| *Gloeobacter violaceus* PCC 7421 | CrtB, CrtD, CrtI, CrtO, CrtW, CruA, CruE, CruF, CruG, CruH, CruP | BAC89685, gll2874, *BAC88808*, gvip032, gvip239, gll2484, gll1923, glr1356, glr1357, gll1922, gll3598 | β-Carotene, Oscillol, Synechoxanthin | Y | Y | [76,77] |
| **Nostocales** | | | | | | |
| *Anabaena variabilis* ATCC 29413 | CrtB, CrtD, CrtH, CrtO, CrtP, CrtQ, CrtR, CrtW (2x), CruA, CruE, CruF, CruH, CruP | Ava_4794, Ava_2342, Ava_3112, Ava_1581, Ava_4795, Ava_0200, Ava_1693, Ava_2048, Ava_3888, Ava_3214, Ava_0036, Ava_1513, Ava_1827, Ava_4521 | ß-Carotene, Canthaxanthin, Echinenone, 4-Hydroxymyxol, Myxol, Synechoxanthin | N | Y | [78] |
| *Nodularia spumigena* CCY9414 | CrtB, CrtD, CrtH, CrtO, CrtP, CrtQ, CrtR, CrtW, CruA, CruE, CruF, CruG, CruH | N9414_14563, N9414_14318, N9414_19217, N9414_21450, N9414_14558, N9414_04980, N9414_01572, N9414_07726, N9414_16776, N9414_21696, N9414_16701, N9414_16696, N9414_03388 | β-Carotene, Canthaxanthin/Echinenone,  Ketomyxol-like, Synechoxanthin, Zeaxanthin/Cryptoxanthin | N | N |  |
| *Nostoc* sp. PCC 7120 | CrtB, CrtD, CrtH, CrtO, CrtP, CrtQ, CrtQ-1, CrtR, CrtW (2x), CruA, CruE, CruF, CruG, CruH, CruP | alr1833, all5123, alr2064, all3744, alr1832, all2382, *BAA05091* (plasmid), alr4009, alr3189, *BAB78246* (plasmid), alr0920, alr2785, all0144, all0143, all3866, alr3524 | ß-Carotene, Echinenone, Ketomyxol glycoside, Myxol glycoside, Synechoxanthin | Y | Y | [79-82] |
| *Nostoc punctiforme* PCC 73102 | CrtB, CrtD, CrtH, CrtO (2x), CrtP, CrtQ, CrtR, CrtW (2x), CruA, CruF, CruG, CruP | Npun02003603, Npun02007228, Npun02004596, Npun02000238, Npun02000982, Npun02003602, Npun02000603, Npun02006805, *ZP_00111258*, *ZP_00345866*, Npun02000486, Npun02002145, Npun02002146, Npun02004577 | ß-Carotene, Echinenone, Ketomyxol glycoside, Myxol glycoside | N | N | [81,83] |
| **Oscillatoriales** | | | | | | |
| *Lyngbya* sp. PCC 8106 | CrtB, CrtD, CrtH, CrtI, CrtO, CrtP, CrtQ, CrtR, CruA, CruE, CruF, CruG, CruH, CruP | L8106_05750, L8106_04976, L8106_26227, L8106_14465, L8106_26402, L8106_05755, L8106_07831, L8106_30215, L8106_12075, L8106_26267, L8106_14400, L8106_14395, L8106_26272, L8106_18117 | β-Carotene, Canthaxanthin/Echinenone,  Myxol-like, Synechoxanthin, Zeaxanthin/Cryptoxanthin | N | N |  |
| *Trichodesmium erythraeum* IMS101 | CrtB, CrtD, CrtH, CrtP, CrtQ, CrtR, CrtX, CruA, CruP | Tery_4010, Tery_4343, Tery_2192, Tery_4011, Tery_3954, Tery_2925, *YP_721820*, Tery_0494, Tery_1762 | β-Carotene, Zeaxanthin/Cryptoxanthin | N | Y | [63] |
| **Prochlorales** | | | | | | |
| *Prochlorococcus marinus* AS9601 | CrtB, CrtH, CrtLb, CrtLe, CrtP, CrtQ, CrtR | A9601_01601, A9601_12211, A9601_11691, A9601_06891, A9601_01611, A9601_01331, A9601_02571 | α-Carotene, Zeaxanthin/Cryptoxanthin | N | Y |  |
| *Prochlorococcus marinus* MIT 9211 | CrtB, CrtH, CrtLb, CrtLe, CrtP, CrtQ, CrtR | P9211_08067, P9211_05912, P9211_03117, P9211_05097, P9211_08062, P9211_08202, P9211_07547 | α-Carotene, Zeaxanthin/Cryptoxanthin | N | Y |  |
| *Prochlorococcus marinus* MIT 9215 | CrtB, CrtH, CrtLb, CrtLe, CrtP, CrtQ, CrtR | P9215_01601, P9215_12511, P9215_11991, P9215_07161, P9215_01611, P9215_01331, P9215_02581 | α-Carotene, Zeaxanthin/Cryptoxanthin | N | Y |  |
| *Prochlorococcus marinus* MIT 9301 | CrtB, CrtH, CrtLb, CrtLe, CrtP, CrtQ, CrtR | P9301_01621, P9301_12221, P9301_11701, P9301_06601, P9301_01631, P9301_01321, P9301_02581 | α-Carotene, Zeaxanthin/Cryptoxanthin | N | Y |  |
| *Prochlorococcus marinus* MIT 9312 | CrtB, CrtH, CrtLb, CrtLe, CrtP, CrtQ, CrtR | PMT9312_0145, PMT9312_1126, PMT9312_1075, PMT9312_0633, PMT9312_0146, PMT9312_0118, PMT9312_0238 | α-Carotene, Zeaxanthin/Cryptoxanthin | N | Y | [63] |
| *Prochlorococcus marinus* MIT 9313^k^ | CrtB, CrtH, CrtLb, CrtLe, CrtP, CrtQ, CrtR | PMT2003, PMT1051, PMT1773, PMT1123, PMT2004, PMT1968, PMT1816 | α-Carotene, Zeaxanthin/Cryptoxanthin | N | Y | [63] |
| *Prochlorococcus marinus* MIT 9515 | CrtB, CrtH, CrtLb, CrtLe, CrtP, CrtQ, CrtR | P9515_01711, P9515_12061, P9515_11541, P9515_06991, P9515_01721, P9515_01291, P9515_02681 | α-Carotene, Zeaxanthin/Cryptoxanthin | N | Y |  |
| *Prochlorococcus marinus* NATL2A^l^ | CrtB, CrtH, CrtLb, CrtLe, CrtP, CrtQ, CrtR | PMN2A_1509, PMN2A_0636, PMN2A_0688, PMN2A_0073, PMN2A_1510, PMN2A_1484, PMN2A_1603 | α-Carotene, Zeaxanthin/Cryptoxanthin | N | Y | [63] |
| *Prochlorococcus marinus* subsp. *marinus* CCMP1375 | CrtB, CrtH, CrtLb, CrtLe, CrtP, CrtQ, CrtR | Pro0166, Pro0584, Pro1136, Pro0790, Pro0167, Pro0136, Pro0266 | α-Carotene, Zeaxanthin/Cryptoxanthin | N | Y |  |
| *Prochlorococcus marinus* subsp. *pastoris* CCMP1986 | CrtB, CrtH, CrtLb, CrtLe, CrtP, CrtQ, CrtR | PMM0143, PMM1115, *CAE19093*, *CAE19092*, PMM0144, PMM0115, PMM0236 | α-Carotene, Zeaxanthin/Cryptoxanthin | N | Y | [84] |
| **Unclassified** | | | | | | |
| *Acaryochloris marina* MBIC11017 | CrtB, CrtH, CrtL, CrtP, CrtQ, CrtR, CruA (2x), CruP | *YP_001519114*, *YP_001515816*, *YP_001520326*, *YP_001519115*, *YP_001517998*, *YP_001517943*, *YP_001519700*, *YP_001516943*, *YP_001516710* | α-Carotene, Zeaxanthin/Cryptoxanthin | N | Y | [85] |
| **Chlorobi** | | | | | | |
| *Chlorobaculum tepidum* TLS | CrtB, CrtC, CrtH, CrtP, CrtQ, CrtU, CruA, CruC, CruD | *AAM72615*, *AAM71547*, *AAM71888*, *AAM72043*, *AAM72642*, *AAM71569*, *AAM71699*, *AAM73205*, *AAM72202* | Chlorobactene, 1’-Hydroxychlorobacetene/  glycoside/glycoside laurate | N | Y | [4,62,86,87] |
| *Chlorobium chlorochromatii* CaD3 | CrtB, CrtC, CrtH, CrtP, CrtQ, CruA, CruC, CruD | Cag_1175, Cag_0393, Cag_1888, Cag_1188, Cag_1590, Cag_0265, Cag_0212, Cag_0595 | Chlorobactene, 1’-Hydroxychlorobacetene/  glycoside/glycoside laurate | N | Y |  |
|  |  |  |  |  |  |  |
| *Chlorobium clathratiforme* BU-1 | CrtB, CrtC, CrtH, CrtP, CrtQ, CrtU, CruA, CruB, CruC, CruD | PphaDRAFT_2098, PphaDRAFT_0849, PphaDRAFT_0104, PphaDRAFT_2540, PphaDRAFT_2291, PphaDRAFT_1677, PphaDRAFT_0832, PphaDRAFT_2724, PphaDRAFT_2155, PphaDRAFT_2526 | Chlorobactene, 1’-Hydroxychlorobacetene/  glycoside/glycoside laurate, Isorenieratene | N | N | [4] |
| *Chlorobium ferrooxidans* DSM 13031 | CrtB, CrtC, CrtH, CrtP, CrtQ, CrtU, CruA, CruC, CruD | CferDRAFT_0237, CferDRAFT_1001, CferDRAFT_0390, CferDRAFT_0138, CferDRAFT_0317, CferDRAFT_2005, CferDRAFT_0992, CferDRAFT_0710, CferDRAFT_0466 | Chlorobactene, 1’-Hydroxychlorobacetene/  glycoside/glycoside laurate | N | N | [4] |
| *Chlorobium limicola* DSM 245 | CrtB, CrtC, CrtH, CrtP, CrtQ, CrtU, CruA, CruC, CruD | ClimDRAFT_1659, ClimDRAFT_1225, ClimDRAFT_1699, ClimDRAFT_2229, ClimDRAFT_2229, ClimDRAFT_1832, ClimDRAFT_2111, ClimDRAFT_0833, ClimDRAFT_0278 | Chlorobactene, 1’-Hydroxychlorobacetene/  glycoside/glycoside laurate | N | N |  |
| *Chlorobium luteolum* DSM 273 | CrtB, CrtC, CrtH, CrtP, CrtQ, CrtU, CruA, CruC, CruD | Plut_1356, Plut_1720, Plut_0626, Plut_1283, Plut_1415, Plut_0435, Plut_1700, Plut_0242, Plut_1188 | Chlorobactene, 1’-Hydroxychlorobacetene/  glycoside/glycoside laurate, Isorenieratene | N | N | [4] |
| *Chlorobium phaeobacteroides* BS1 | CrtB, CrtC, CrtH, CrtP, CrtQ, CrtU, CruA, CruB CruC, CruD | Cphamn1DRAFT_2436, Cphamn1DRAFT_2230, Cphamn1DRAFT_2570, Cphamn1DRAFT_2471, Cphamn1DRAFT_2632, Cphamn1DRAFT_2517, Cphamn1DRAFT_3315, Cpham1DRAFT_2874, Cphamn1DRAFT_3003, Cphamn1DRAFT_2103 | Chlorobactene, 1’-Hydroxychlorobacetene/  glycoside/glycoside laurate, Isorenieratene | N | N |  |
| *Chlorobium phaeobacteroides* DSM 266 | CrtB, CrtC, CrtH, CrtP, CrtQ, CrtU, CruA, CruB, CruC, CruD | Cpha266_1738, Cpha266_0428, Cpha266_0874, Cpha266_1173, Cpha266_0830, Cpha266_0659, Cpha266_0192, Cpha266_0474, Cpha266_2356, Cpha266_1013 | Chlorobactene, 1’-Hydroxychlorobacetene/  glycoside/glycoside laurate, Isorenieratene | N | Y | [4,88] |
| *Chlorobium phaeovibrioides* DSM 265 | CrtB, CrtC, CrtH, CrtP, CrtQ, CrtU, CruA, CruC, CruD | Cvib_0699, Cvib_1502, Cvib_1153, Cvib_1032, Cvib_1233, Cvib_0486, Cvib_1484, Cvib_0308, Cvib_0769 | Chlorobactene, 1’-Hydroxychlorobacetene/  glycoside/glycoside laurate | N | Y | [4] |
| *Prosthecochloris aestuarii* DSM 271 | CrtB, CrtC, CrtD, CrtH, CrtP, CrtU, CruA, CruC, CruD | PaesDRAFT_0157, PaesDRAFT_0561, PaesDRAFT_1494, PaesDRAFT_1466, PaesDRAFT_1265, PaesDRAFT_0420, PaesDRAFT_1729, PaesDRAFT_2266, PaesDRAFT_0285 | Chlorobactene, 1’-Hydroxychlorobacetene/  glycoside/glycoside laurate, Isorenieratene | N | N | [4] |
| **Chloroflexi** | | | | | | |
| *Chloroflexus aggregans* DSM 9485 | CrtI, CrtO, CrtY | CaggDRAFT_0197, CaggDRAFT_2957, CaggDRAFT_2028 | β-Carotene, γ-Carotene, 1-OH-γ-Carotene/glucoside, 4-Keto-1-OH-γ-Carotene | N | N | [4] |
| *Chloroflexus aurantiacus* J-10-fl | CrtB, CrtI, CrtO, CrtY | CaurDRAFT_2153, CaurDRAFT_2173, CaurDRAFT_270, CaurDRAFT_0959 | β-Carotene, γ-Carotene, 1-OH-γ-Carotene/glucoside/FA | N | Y | [4] |
|  |  |  |  |  |  |  |
| *Herpetosiphon aurantiacus* ATCC 23779 | CrtB, CrtI, CrtO, CruA | HaurDRAFT_5057, HaurDRAFT_1159, HaurDRAFT_3603, HaurDRAFT_1188 | β-Carotene, γ-Carotene, 1-OH-γ-Carotene/glucoside/FA | N | Y | [89] |
| *Roseiflexus castenholzii* DSM 13941 | CrtB, CrtI (2x), CrtL, CrtO | Rcas_1752, Rcas_1585, Rcas_3488, Rcas_2711, Rcas_1486 | Methoxy-keto-myxocoxanthin, Keto-myxocoxanthin glucoside fatty acid ester | N | Y | [90] |
| *Roseiflexus* sp. RS-1 | CrtB, CrtI (2x), CrtL, CrtO | RoseRS_2117, RoseRS_0943, RoseRS_2155, RoseRS_2643, RoseRS_3475 | Methoxy-keto-myxocoxanthin, Keto-myxocoxanthin glucoside fatty acid ester | Y | Y |  |
| **Deinococcus/Thermus** | | | | | | |
| *Deinococcus geothermalis* DSM 11300 | CrtB, CrtD, CrtI, CrtL, CrtO | Dgeo_0523, Dgeo_2306, Dgeo_0524, Dgeo_0857, Dgeo_2310 | Deinoxanthin | N | Y |  |
| *Deinococcus radiodurans* R1 | CrtB, CrtD, CrtI, CrtL, CrtO | DR0862, DR2250, AAF10439, AAF10377, AAF09686 | Deinoxanthin | N | Y | [56,57,91,92] |
| *Thermus thermophilus* HB27 | CrtB (plasmid), CrtI, CrtYcd, P450 | *P37270*, TT_P0066, *YP_00643*, *CYP175A1* | Zeaxanthin | N | Y | [93,94] |
| *Thermus thermophilus* HB8 | CrtB (plasmid), CrtI, CrtYcd, P450 | TTHB101, TTHB109, *YP_145343*, TTHB103 | Zeaxanthin | N | Y | [94] |
| **Planctomycetes** | | | | | | |
| *Gemmata obscuriglobus* UQM 2246 | CrtN, CrtNb | *ZP_02732075*, *ZP_02732075* | 4,4’-Diapolycopene oxide/4,4’-Diaponeurosporene oxide | N | ? |  |
| *Rhodopirellula baltica* SH 1 | CrtN, CrtNb | *NP_869339*, *NP_870237* | 4,4’-Diapolycopene oxide/4,4’-Diaponeurosporene oxide | N |  |  |
| Uncultured Marine Bacterium HF10_49E08 | CrtBI, CrtY | *ABL97829*, *ABL97830* | β-Carotene | Y | N/A |  |
| **Crenarchaeota** | | | | | | |
| *Metallosphaera sedula* DSM 5348 | CrtB, CrtI, CrtYcd, CrtZ | *YP_001191163*, Msed_1073, Msed_1076, *YP_001191162* | Zeaxanthin | N | N |  |
| *Picrophilus torridus* DSM 9790 | CrtB, CrtI, CrtYcd, CrtZ | *YP_024313*, PTO1532, PTO1534, *YP_024309* | Zeaxanthin | N | Y |  |
|  |  |  |  |  |  |  |
| *Sulfolobus acidocaldarius* DSM 639 | CrtB, CrtI, CrtYcd, CrtZ | *YP_256333*, Saci_1732, Saci_1735, *YP_256332* | Zeaxanthin | N | Y |  |
| *Sulfolobus solfataricus* P2 | CrtB, CrtI, CrtYcd, CrtZ | *NP_344224*, SSO2907, SSO2904, SSO2906 | Zeaxanthin | N | Y | [95] |
| **Euryarchaeota** | | | | | | |
| *Methanoculleus marisnigri* JR1 | CrtB, CrtEb, CrtI | *YP_001046034*, *YP_001046035*, Memar_0116 | Linear C50 carotenoids | N | Y |  |
| *Methanothermobacter thermautotrophicus* Delta H | CrtB, CrtEb, CrtI | *NP_276914*, *NP_276915*, MTH1807 | Linear C50 carotenoids | N | Y |  |
| Uncultured Marine Bacterium HF10_29C11 | CrtB, CrtI, CrtY | *ABL97779*, *ABL97778*, *ABL97780* | β-Carotene | N/A | N/A |  |
| **Haloarchaea** |  |  |  |  |  |  |
| *Haloarcula marismortui* ATCC 43049 | CrtB, CrtEb, CrtI (2x), CrtYcd | rrnAC2069, rrnAC0320, rrnAC0321, rrnAC1902, *YP_136628* | β-Carotene^c^, Linear C50 carotenoids | Y | Y |  |
| *Halobacterium* sp. NRC-1 | CrtB (2x), CrtEb, CrtI (2x) | VNG1458G, VNG1680G, VNG1682C, VNG1684G, VNG1755G | Linear C50 carotenoids | Y | Y |  |
| *Haloquadratum walsbyi* DSM 16790 | CrtB, CrtEb, CrtI (2x), CrtYcd | HQ2860A, HQ2862A, HQ1794A, HQ2863A, *YP_656805* | β-Carotene, Linear C50 carotenoids | Y | Y |  |
| *Halorubrum lacusprofundi* ATCC 49239 | CrtB, CrtEb, CrtI, CrtYcd | HlacDRAFT_0809, HlacDRAFT_1351, HlacDRAFT_1352, *ZP_02017097* | β-Carotene, Linear C50 carotenoids | Y | N |  |
| *Natronomonas pharaonis* DSM 2160 | CrtB, CrtEb, CrtI (2x), CrtYcd | NP4770A, NP4766A, NP0204A, NP4764A, *YP_325986* | β-Carotene, Linear C50 carotenoids | Y | Y |  |
| **Fungi** | | | | | | |
| *Aspergillus niger* | CrtBYcd, CrtI, CAO-2, YLO-1 | *XP_001391172*, *XP_001391204*, *XP_001401639*, *XP_001389346* | β-Carotene, Neurosporaxanthin | Y | Y |  |
| *Aspergillus oryzae* RIB40 | CrtBYcd, CrtI, CAO-2, YLO-1 | *XP_001824519*, *XP_001824518*, *XP_001821249*, *XP_001816713* | β-Carotene, Neurosporaxanthin | Y | Y |  |
| *Cercospora nicotianae* ATCC 18366 | CrtI | *AAB86988* | β-Carotene | N/A | N | [96,97] |
| *Gibberella fujikuroi* IMI58289 | CrtBYcd (CarRA), CrtI (CarB), CAO-2 (CrtT) | *CAD19988*, *CAD19989*, *CAL90971* | β-Carotene, Neurosporaxanthin | N/A | N | [98-100] |
| *Gibberella zeae* PH-1 | CrtBYcd, CrtI, CAO-2, YLO-1 | *XP_383242*, *XP_383241*, *XP_382801*, *XP_390136* | β-Carotene, Neurosporaxanthin | Y | Y |  |
| *Mucor circinelloides* | CrtBM (CarRP) | *Q9UUQ6* | β-Carotene | N/A | N | [101,102] |
| *Neurospora crassa* | CrtBYcd (AL-2), CrtI (AL-1), CAO-2, YLO-1 | *CAE76609*, *P21334*, *XP_001727958*, *XP_957628* | β-Carotene, Neurosporaxanthin | N/A | N | [103-106] |
| *Phaeosphaeria nodorum* SN15 | CrtBYcd, CrtI, CAO-2, YLO-1 | *XP_001791029*, *XP_001791032*, *XP_001792684*, *XP_001796173* | β-Carotene, Neurosporaxanthin | Y | N |  |
| *Phycomyces blakesleeanus* | CrtBYcd (CarRA), CrtI (CarB) | *CAB86388*, *P54982* | β-Carotene | N/A | N | [107-109] |
| *Podospora anserina* | CrtBYcd, CrtI, CAO-2, YLO-1 | *XP_00190692*, *XP_001906937*, *XP_00193611*, *XP_001903919* | β-Carotene, Neurosporaxanthin | Y | Y |  |
| *Ustilago maydis* 521 | CrtBYcd, CrtI | *XP_762434*, *XP_760357* | β-Carotene | Y | Y |  |
| *Xanthophyllomyces dendrorhous* | CrtBYcd (CrtYB), CrtI | *AAY33923*, *AAA19428* | Astaxanthin | N/A | N | [110-112] |
| **Photosynthetic Eukaryotes** | | | | | | |
| *Chlamydomonas reinhardtii* | PSY, CRTISO, LYCB, LYCE, PDS, ZDS, BKT, CHYB, CruP (2x), ZEP | *XP_001701192*, *XP_001698231*, *AAX54906*, *XP_001696529*, *XP_001690859*, *XP_001700786*, *XP_001698699*, *XP_001698698*, *XP_001692181*, *XP_001696289*, *AAO34404* | β-Carotene, Epoxides, Lutein | Y? | Y |  |
| *Cyanidioschyzon merolae* | PSY, CRTISO, LYCB, PDS, ZDS, CrtR, CruP | CMM166C, CMN268C, CMK050C, CMK151C, CMT061C, CMV041C, CMC032C | Zeaxanthin | N/A | Y | [113] |
| *Dunaliella salina* | LYCB | *ACA34345* | β-Carotene | N/A | Y | [114,115] |
| *Galdieria sulphuraria* | PSY, CRTISO, LYCB, PDS, CrtR | Contig06203.g3.t1, Gs12840.1, Contig02802.g56.t1, Gs46720.1, Gs39970.1 | Zeaxanthin | N/A | N |  |
| *Haematococcus pluvialis* | PSY, BKT, CHYB | *AAK15621*, *AAT35555*, *AAD54243* | Astaxanthin | N/A | N/A | [116-119] |
| *Ostreococcus lucimarinus* CCE9901 | PSY, LYCB, LYCE, PDS (2x), CHYB, CruP, ZEP, VDE | *XP_001418049*, *XP_001422490*, *XP_001422489*, *XP_001421697*, *XP_001420014*, *XP_001419973*, *XP_001415446*, *ABO99857*, *ABO99997* | β-Carotene, Epoxides, Lutein | N | Y |  |
| *Phaeodactylum tricornutum* | PSY, LYCB, PDS, ZDS, CruP, ZEP, VDE | estExt_fgenesh1_pg.C_chr_50182, estExt_Phatr1_ua_kg.C_chr_10036, fgenesh1_pg.C_chr_24000086 (short), estExt_gwp_gw1.C_chr_80030 (short), fgenesh1_pg.C_chr_4000121, fgenesh1_pg.C_chr_4000464, fgenesh1_pg.C_chr_4000463 | β-Carotene, Epoxides, Lutein | N/A | Y |  |
| *Thalassiosira pseudonana* | PSY, LYCB, PDS, ZDS, ZEP, VDE | estExt_thaps1_ua_kg.C_chr_50183, thaps1_ua_kg.chr_2000231, fgenesh1_pg.C_chr_6000679 (short), fgenesh1_pg.C_chr_1000517 (short), estExt_thaps1_ua_kg.C_chr_60282, thaps1_ua_kg.chr_8000103 | β-Carotene, Epoxides, Lutein | N/A | Y |  |

^a^Underlined data indicates evidene present in the literature, with the relative references cited in the right-most column.

^b^Only pathway endproducts are indicated, unless a specific literature reference is cited, due to the difficulty in identifying accumulatory intermediates solely from sequence homology.

^c^β-Carotene production is considered to be demonstrated in these organisms by the demonstration of a functional rhodopsin without the addition of exogenous carotenoid cofactors.

^d^*Myxococcus xanthus* DK 1622 is considered representative of *M. xanthus* DK 1050, for which experimental evidence but no genome sequence is available, due to nearly 100% sequence homology between strains.

^e^*Staphylococcus aureus* Newman is considered representative of *S. aureus* str. COL, JH1, JH9, MRSA252 (no CrtM), MSSA476, MW2, Mu3, Mu50, N315 (no CrtNb), NCTC 8325 and USA300 due to nearly 100% sequence homology between strains.

^f^*Corynebacterium glutamicum* ATCC 13032 (Bielefeld) is considered representative of *C. glutamicum* str. ATCC 13032 (Kitasato), R. and MJ233C, for which experimental evidence but no second CrtI homolog exists, due to nearly 100% sequence homology between strains.

^g^*Mycobacterium* sp. MCS is considered representative of *Mycobacterium* spp. KMS and JLS due to nearly 100% sequence homology between strains.

^h^*Mycobacterium avium* subsp. Paratuberculosis K-10 is considered representative of *M. avium* 104 due to nearly 100% sequence homology between strains.

^i^*Clavibacter michiganensis michiganensis* NCPPB 382 is considered representative of *C. michiganensis* subsp. *sepedonicus* due to nearly 100% sequence homology between strains.

^j^*Synechococcus elongaus* PCC 7942 is considered representative of *S. elongaus* PCC 6301 due to nearly 100% sequence homology between strains.

^l^*Prochlorococcus marinus* MIT 9313 is considered representative of *P. marinus* MIT 9303 due to nearly 100% sequence homology between strains.

^k^*Prochlorococcus marinus* NATL2A is considered representative of *P. marinus* NATL1A due to nearly 100% sequence homology between strains.

**References:**

1. Nishida Y, Adachi K, Kasai H, Shizuri Y, Shindo K, et al. (2005) Elucidation of a carotenoid biosynthesis gene cluster encoding a novel enzyme, 2,2*'*-β-hydroxylase, from *Brevundimonas* sp. strain SD212 and combinatorial biosynthesis of new or rare xanthophylls. Appl Environ Microbiol 71: 4286-4296.

2. Yokoyama A, Miki W, Izumida H, Shizuri Y (1996) New trihydroxy-keto-carotenoids isolated from an astaxanthin-producing marine bacterium. Biosci Biotechnol Biochem 60: 200-203.

3. Tao L, Rouvière PE, Cheng Q (2006) A carotenoid synthesis gene cluster from a non-marine *Brevundimonas* that synthesizes hydroxylated astaxanthin. Gene 379: 101-108.

4. Takaichi S (1999) Carotenoids and carotenogenesis in anoxygenic photosynthetic bacteria. In: Frank HA, Young AJ, Britton G, Cogdell RJ, editors. The photochemistry of carotenoids. New York, NY: Kluwer Academic Publishers. pp. 39-69.

5. Giraud E, Hannibal L, Fardoux J, Jaubert M, Jourand P, et al. (2004) Two distinct *crt* gene clusters for two different functional classes of carotenoid in *Bradyrhizobium*. J Biol Chem 279: 15076-15083.

6. Hannibal L, Lorquin J, D'Ortoli NA, Garcia N, Chaintreuil C, et al. (2000) Isolation and characterization of canthaxanthin biosynthesis genes from the photosynthetic bacterium *Bradyrhizobium* sp. strain ORS278. J Bacteriol 182: 3850-3853.

7. Van Dien SJ, Marx CJ, O'Brien BN, Lidstrom ME (2003) Genetic characterization of the carotenoid biosynthetic pathway in *Methylobacterium extorquens* AM1 and isolation of a colorless mutant. Appl Environ Microbiol 69: 7563-7566.

8. Larsen RA, Wilson MM, Guss AM, Metcalf WW (2002) Genetic analysis of pigment biosynthesis in *Xanthobacter autotrophicus* Py2 using a new, highly efficient transposon mutagenesis system that is functional in a wide variety of bacteria. Arch Microbiol 178: 193-201.

9. Biebl H, Allgaier M, Tindall BJ, Koblizek M, Lünsdorf H, et al. (2005) *Dinoroseobacter shibae* gen. nov., sp. nov., a new aerobic phototrophic bacterium isolated from dinoflagellates. Int J Syst Evol Microbiol 55: 1089-1096.

10. Lee JT, Kim YT (2006) Clonging and characterization of the astaxanthin biosynthesis gene cluster from the marine bacterium *Paracoccus haeundaensis*. Gene 370: 86-95.

11. Lee JH, Kim YS, Choi T-J, Lee WJ, Kim YT (2004) *Paracoccus haeundaensis* sp. nov., a Gram-negative, halophilic, astaxanthin-producing bacterium. Int J Syst Evol Microbiol 54: 1699-1702.

12. Misawa N, Satomi Y, Kondo K, Yokoyama A, Kajiwara S, et al. (1995) Structure and functional analysis of a marine bacterial carotenoid biosynthesis gene cluster and astaxanthin biosynthetic pathway proposed at the gene level. J Bacteriol 177: 6575-6584.

13. Yokoyama A, Izumida H, Miki W (1994) Production of astaxanthin and 4-ketozeaxanthin by the marine bacterium *Agrobacterium aurantiacum*. Biosci Biotechnol Biochem 58: 1842-1844.

14. Pasamontes L, Hug D, Tessier M, Hohmann H-P, Schierle J, et al. (1997) Isolation and characterization of the carotenoid biosynthesis genes of *Flavobacterium* sp. strain R1534. Gene 185: 35-41.

15. Berry A, Janssens D, Hümbelin M, Jore JPM, Hoste B, et al. (2003) *Paracoccus zeaxanthinifaciens* sp. nov., a zeaxanthin-producing bacterium. Int J Syst Evol Microbiol 53: 231-238.

16. Armstrong GA, Alberti M, Leach F, Hearst JE (1989) Nucleotide sequence, organization, and nature of the protein products of the carotenoid biosynthesis gene cluster of *Rhodobacter capsulatus*. Mol Gen Genet 216: 254-268.

17. Lang HP, Cogdell RJ, Gardiner AT, Hunter CN (1994) Early steps in carotenoid biosynthesis: sequences and transcriptional analysis of the *crtI* and *crtB* genes of *Rhodobacter sphaeroides* and overexpression and reactivation of *crtI* in *Escherichia coli* and *R. sphaeroides*. J Bacteriol 176: 3859-3869.

18. Lang HP, Cogdell RJ, Takaichi S, Hunter CN (1995) Complete DNA sequence, specific Tn*5* insertion map, and gene assignment of the carotenoid biosynthesis pathway of *Rhodobacter sphaeroides*. J Bacteriol 177: 2064-2073.

19. Béjà O, Aravind L, Koonin EV, Suzuki MT, Hadd A, et al. (2000) Bacterial rhodopsin: Evidence for a new type of phototrophy in the sea. Science 289: 1902-1906.

20. Takeyama H, Sunarjo J, Yamada A, Matsumura H, Kusakabe E, et al. (1996) β-Carotene production in a novel hydrogen-producing marine photosynthetic bacterium *Rhodovulum sulfidophilum* expressing the *Erythrobacter longus* OCh101 *crtI* and *crtY* genes. J Mar Biotechnol 4: 224-229.

21. Kobližek M, Béjà O, Bidigare RR, Christensen S, Benetiz-Nelson B, et al. (2003) Isolation and characterization of *Erythrobacter* sp. strains from the upper ocean. Arch Microbiol 180: 327-338.

22. Tao L, Schenzle A, Odom JM, Cheng Q (2005) Novel carotenoid oxidase involved in biosynthesis of 4,4*'*-diapolycopene dialdehyde. Appl Environ Microbiol 71: 3294-3301.

23. Kovács ÁT, Rákhely G, Kovács KL (2003) Genes involved in the biosynthesis of photosynthetic pigments in the purple sulfur photosynthetic bacterium *Thiocapsa roseopersicina*. Appl Environ Microbiol 69: 3093-3102.

24. Harada J, Nagashima KVP, Takaichi S, Misawa N, Matsuura K, et al. (2001) Phytoene desaturase, CrtI, of the purple photosynthetic bacterium, *Rubrivivax gelatinosus*, produces both neurosporene and lycopene. Plant Cell Physiol 42: 1112-1118.

25. Ouchane S, Picaud M, Vernotte C, Reiss-Husson F, Astier C (1997) Pleiotropic effects of *puf* interposon mutagenesis on carotenoid biosynthesis in *Rubrivivax gelatinosus*. J Biol Chem 272: 1670-1676.

26. Gerjets T, Steiger S, Sandmann G (2009) Catalytic properties of the expressed acyclic carotenoid 2-ketolases from *Rhodobacter capsulatus* and *Rubrivivax gelatinosus*. Biochim Biophys Acta 1791: 125-131.

27. Harada J, Takaichi S, Nagashima KVP, Matsuura K, Shimada K. Functional analysis of spheroidene mono-oxygenase, CrtA, of the purple photosynthetic bacterium, *Rubrivivax gelatinosus*; 2001; Brisbane, Australia. CSIRO. pp. S2-025.

28. Ouchane S, Picaud M, Vernotte C, Astier C (1997) Photooxidative stress stimulates illegitimate recombination and mutability in carotenoid-less mutants of *Rubrivivax gelatinosus*. The EMBO Journal 16: 4777-4787.

29. Pinta V, Ouchane S, Picaud M, Takaichi S, Astier C, et al. (2003) Characterization of unusual hydroxy- and ketocarotenoids in *Rubrivivax gelatinosus*: involvement of enzyme CrtF or CrtA. Arch Microbiol 179: 354-362.

30. Steiger S, Astier C, Sandmann G (2000) Substrate specificity of the expressed carotenoid 3,4-desaturase from *Rubrivivax gelatinosus* reveals the detailed reaction sequence to spheroidene and spirilloxanthin. Biochem J 349: 635-640.

31. Steiger S, Mazet A, Sandmann G (2003) Heterologous expression, purification, and enzymatic characterization of the acyclic carotenoid 1,2-hydratase from *Rubrivivax gelatinosus*. Arch Biochem Biophys 414: 51-58.

32. Stickforth P, Sandmann G (2007) Kinetic variations determine the product pattern of phytoene desaturase from *Rubrivivax gelatinosus*. Arch Biochem Biophys 461: 235-241.

33. Fuchs BM, Spring S, Teeling H, Quast C, Wulf J, et al. (2007) Characterization of a marine gammaproteobacterium capable of aerobic anoxygenic photosynthesis. Proc Nat Acad Sci USA 104: 2891-2896.

34. Lehner A, Grimm M, Rattei T, Ruepp A, Frishman D, et al. (2006) Cloning and characterization of *Enterobacter sakazakii* pigment genes and *in situ* spectroscopic analysis of the pigment. FEMS Microbiol Lett 265: 244-248.

35. Sedkova N, Tao L, Rouvière PE, Cheng Q (2005) Diversity of carotenoid synthesis gene clusters from environmental *Enterobacteriaceae* strains. Appl Environ Microbiol 71: 8141-8146.

36. Hundle B, Alberti M, Nievelstein V, Beyer P, Klening H, et al. (1994) Functional assignment of *Erwinina herbicola* Eho10 carotenoid genes expressed in *Escherichia coli*. Mol Gen Genet 245: 406-416.

37. Hundle BS, Beyer P, Kleinig H, Englert G, Hearst JE (1991) Carotenoids of *Erwinia herbicola* and an *Escherichia coli* HB101 strain carrying the *Erwinia herbicola* carotenoid gene cluster. Photochem Photobiol 54: 89-93.

38. Misawa N, Nakagawa M, Kobayashi K, Yamano S, Izawa Y, et al. (1990) Elucidation of the *Erwinia uredovora* carotenoid biosynthetic pathway by functional analysis of gene products expressed in *Escherichia coli*. J Bacteriol 172: 6704-6712.

39. Botella JA, Murillo FJ, Ruiz-vázquez R (1995) A cluster of structural and regulatory genes for light-induced carotenogenesis in *Myxococcus xanthus*. Eur J Biochem 233: 238-248.

40. Iniesta AA, Cervantes M, Murillo FJ (2007) Cooperation of two carotene desaturases in the production of lycopene in *Myxococcus xanthus*. FEBS J 274: 4306-4314.

41. Iniesta AA, Cervantes M, Murillo FJ (2008) Conversion of the lycopene monocyclase of *Myxococcus xanthus* into a bicyclase. Appl Microbiol Biotechnol 79: 793-802.

42. Gómez-Consarnau L, González JM, Coll-Lladó M, Gourdon P, Pascher T, et al. (2007) Light stimulates growth of proteorhodopsin-containing marine flavibacteria. Nature 445: 210-213.

43. Teramoto M, Rählert N, Misawa N, Sandmann G (2004) 1-Hydroxy monocyclic carotenoid 3,4-dehydrogenase from a marine bacterium that produces myxol. FEBS Lett 570: 184-188.

44. Teramoto M, Takaichi S, Inomata Y, Ikenaga H, Misawa N (2003) Structural and functional analysis of a lycopene β-monocyclase gene isolated from a unique marine bacterium that produces myxol. FEBS Lett 545: 120-126.

45. Yokoyama A, Miki W (1995) Isolation of myxol from a marine bacterium *Flavobacterium* sp. associated with a marine sponge. Fish Sci 61: 684-686.

46. Tao L, Yao H, Kasai H, Misawa N, Cheng Q (2006) A carotenoid synthesis gene cluster from *Algoriphagus* sp. KK10202C with a novel fusion-type lycopene β-cyclase gene. Mol Genet Genomics 276: 79-86.

47. Lutnaes BF, Oren A, Liaaen-Jensen S (2002) New C_40_-carotenoid acyl glycoside as principal carotenoid in *Salinibacter ruber*, an extremely halophilic eubacterium. J Nat Prod 65: 1340-1343.

48. Balashov SP, Imasheva ES, Boichenko VA, Antón J, Wang JM, et al. (2005) Xanthorhodopsin: a proton pump with a light-harvesting carotenoid antenna. Science 309: 2061-2064.

49. Wieland B, Feil C, Gloria-Maercker E, Thumm G, Lechner M, et al. (1994) Genetic and biochemical analyses of the biosynthesis of the yellow carotenoid 4,4*'*-diaponeurosporene of *Staphylococcus aureus*. J Bacteriol 176: 7719-7726.

50. Pelz A, Wieland K-P, Putzbach K, Hentschel P, Albert K, et al. (2005) Structure and biosynthesis of staphyloxanthin from *Staphylococcus aureus*. J Biol Chem 280: 32493-32498.

51. Takaichi S, Inoue K, Akaike M, Kobayashi M, Oh-oka H, et al. (1997) The major carotenoid in all known species of heliobacteria is the C30 carotenoid 4,4'-diaponeurosporene, not neurosporene. Arch Microbiol 168: 277-281.

52. Krubasik P, Kobayashi M, Sandmann G (2001) Expression and functional analysis of a gene cluster involved in the synthesis of decaprenoxanthin reveals the mechanisms for C_50_ carotenoid formation. Eur J Biochem 268: 3702-3708.

53. Tao L, Yao H, Cheng Q (2007) Genes from a *Dietzia* sp. for synthesis of C_40_ and C_50_ ß-cyclic carotenoids. Gene 386: 90-97.

54. Matsui T, Maruhashi K (2004) Isolation of carotenoid-deficient mutant from alkylated dibenzothiophene desulfurizing nocardioform bacteria, *Gordonia* sp. TM414. Curr Microbiol 48: 130-134.

55. Viveiros M, Krubasik P, Sandmann G, Houssaini-Iraqui M (2000) Structural and functional analysis of the gene cluster encoding carotenoid biosynthesis in *Mycobacterium aurum* A+. FEMS Microbiol Lett 187: 95-101.

56. Tao L, Picataggio S, Rouvière PE, Cheng Q (2004) Asymmetrically acting lycopene β-cyclases (CrtLm) from non-photosynthetic bacteria. Mol Genet Genomics 271: 180-188.

57. Tao L, Cheng Q (2004) Novel ß-carotene ketolases from non-photosynthetic bacteria for canthaxanthin synthesis. Mol Genet Genomics 272: 530-537.

58. Kohl W, Achenbach H, Reichenbach H (1983) The pigments of *Brevibacterium linens*: aromatic carotenoids. Phytochemistry 22: 207-210.

59. Krubasik P, Sandmann G (2000) A carotenogenic gene cluster from *Brevibacterium linens* with novel lycopene cyclase genes involved in the synthesis of aromatic carotenoids. Mol Gen Genet 263: 423-432.

60. Cheng Q (2006) Structural diversity and functional novelty of new carotenoid biosynthesis genes. J Ind Microbiol Biotechnol 33: 552-559.

61. Krügel H, Krubasik P, Weber K, Saluz HP, Sandmann G (1999) Functional analysis of genes from *Streptomyces griseus* involved in the synthesis of isorenieratene, a carotenoid with aromatic end groups, revealed a novel type of carotenoid desaturase. Biochim Biophys Acta 1439: 57-64.

62. Maresca JA, Graham JE, Wu M, Eisen JA, Bryant DA (2007) Identification of a fourth family of lycopene cyclases in photosynthetic bacteria. Proc Nat Acad Sci USA 104: 11784-11789.

63. Maresca JA, Graham JE, Bryant DA (2008) The biochemical basis for structural diversity in the carotenoids of chlorophototrophic bacteria. Photosynth Res 97: 121-140.

64. Takaichi S, Mochimaru M (2007) Carotenoids and carotenogenesis in cyanobacteria: unique ketocarotenoids and carotenoid glycosides. Cell Mol Life Sci 64: 2607-2619.

65. Chamovitz D, Pecker I, Hirschberg J (1991) The molecular basis of resistance to the herbicide norflurazon. Plant Mol Biol 16: 967-974.

66. Chamovitz D, Misawa N, Sandmann G, Hirschberg J (1992) Molecular cloning and expression in *Escherichia coli* of a cyanobacterial gene coding for phytoene synthase, a carotenoid biosynthesis enzyme. FEBS Lett 296: 305-310.

67. Cunningham Jr. FX, Sun Z, Chamovitz D, Hirschberg J, Gantt E (1994) Molecular structure and enzymatic function of lycopene cyclase from the cyanobacterium *Synechococcus* sp. strain PCC7942. Plant Cell 6: 1107-1121.

68. Martínez-Férez IM, Vioque A (1992) Nucleotide sequence of the phytoene desaturase gene from *Synechocystis* sp. PCC 6803 and characterization of a new mutation which confers resistance to the herbicide norflurazon. Plant Mol Biol 18: 981-983.

69. Breitenbach J, Fernández-González B, Vioque A, Sandmann G (1998) A higher-plant type ζ-carotene desaturase in the cyanobacterium *Synechocystis* PCC6803. Plant Mol Biol 36: 725-732.

70. Breitenbach J, Vioque A, Sandmann G (2001) Gene *sll0033* from *Synechocystis* 6803 encodes a carotene isomerase involved in the biosynthesis of all-*E* lycopene. Z Naturforsch C 56: 915-917.

71. Fernández-González B, Sandmann G, Vioque A (1997) A new type of asymmetrically acting β-carotene ketolase is required for the synthesis of echinenone in the cyanobacterium *Synechocystis* sp. PCC 6803. J Biol Chem 272: 9728-9733.

72. Martínez-Férez I, Fernández-González B, Sandmann G, Vioque A (1994) Cloning and expression in *Escherichia coli* of the gene for phytoene synthase from the cyanobacterium *Synechocystis* sp. PCC6803. Biochim Biophys Acta 1218: 145-152.

73. Masamoto K, Misawa N, Kaneko T, Kikuno R, Toh H (1998) β-Carotene hydroxylase gene from the cyanobacterium *Synechocystis* sp. PCC6803. Plant Cell Physiol 39: 560-564.

74. Masamoto K, Wada H, Kaneko T, Takaichi S (2001) Identification of a gene required for *cis-*to*-trans* carotene isomerization in carotenogenesis of the cyanobacterium *Synechocystis* sp. PCC 6803. Plant Cell Physiol 42: 1398-1402.

75. Iwai M, Maoka T, Ikeuchi M, Takaichi S (2008) 2,2'-β-Hydroxylase (CrtG) is involved in carotenogenesis of both nostoxanthin and 2-hydroxymyxol 2'-fucoside in *Thermosynechococcus elongatus* strain BP-1. Plant Cell Physiol 49: 1678-1687.

76. Steiger S, Jackisch Y, Sandmann G (2005) Carotenoid biosynthesis in *Gloeobacter violaceus* PCC4721 involves a single crtI-type phytoene desaturase instead of typical cyanobacterial enzymes. Arch Microbiol 184: 207-214.

77. Tsuchiya T, Takaichi S, Misawa N, Maoka T, Miyashita H, et al. (2005) The cyanobacterium *Gloeobacter violaceus* PCC 7421 uses bacterial-type phytoene desaturase in carotenoid biosynthesis. FEBS Lett 579: 2125-2129.

78. Takaichi S, Mochimaru M, Moaka T (2006) Presence of free myxol and 4-hydroxymyxol and Absence of myxol Glycosides in *Anabaena variabilis* ATCC 29413, and proposal of biosynthetic pathway of carotenoids. Plant Cell Physiol 47: 211-216.

79. Linden H, Vioque A, Sandmann G (1993) Isolation of a carotenoid biosynthesis gene coding for ζ-carotene desaturase from *Anabaena* PCC 7120 by heterologous complementation. FEMS Microbiol Lett 106: 99-104.

80. Mochimaru M, Masukawa H, Takaichi S (2005) The cyanobacterium *Anabaena* sp PCC 7120 has two distinct beta-carotene ketolases: CrtO for echinenone and CrtW for ketomyxol synthesis. FEBS Lett 579: 6111-6114.

81. Takaichi S, Mochimaru M, Moaka T, Katoh H (2005) Myxol and 4-ketomyxol 2'-fucosides, not rhamnosides, from *Anabaena* sp. PCC 7120 and *Nostoc punctiforme* PCC 73102, and proposal for the biosynthetic pathway of carotenoids. Plant and Cell Physiology 46: 497-504.

82. Jung K-H, Trivedi VD, Spudich JL (2003) Demonstration of a sensory rhodopsin in eubacteria. Mol Microbiol 47: 1513-1522.

83. Steiger S, Sandmann G (2004) Cloning of two carotenoid ketolase genes from *Nostoc punctiforme* for the heterologous production of canthaxanthin and astaxanthin. Biotechnol Lett 26: 813-817.

84. Stickforth P, Steiger S, Hess WR, Sandmann G (2003) A novel type of lycopene ε-cyclase in the marine cyanobacterium *Prochlorococcus marinus* MED4. Arch Microbiol 179: 409-415.

85. Miyashita H, Adachi K, Kurano N, Ikemoto H, Chihara M, et al. (1997) Pigment composition of a novel oxygenic photosynthetic prokaryote containing chlorophyll *d* as the major chlorophyll. Plant Cell Physiol 38: 274-281.

86. Maresca JA, Bryant DA (2006) Two genes encoding new carotenoid-modifying enzymes in the green sulfur bacterium *Chlorobium tepidum*. J Bacteriol 188: 6217-6223.

87. Frigaard N-U, Maresca JA, Yunker CE, Jones AD, Bryant DA (2004) Genetic manipulation of carotenoid biosynthesis in the green sulfur bacterium *Chlorobium tepidum*. J Bacteriol 186: 5210-5220.

88. Maresca JA, Romberger SP, Bryant DA (2008) Isorenieratene biosynthesis in green sulfur bacteria requires the cooperative actions of two carotenoid cyclases. J Bacteriol 190: 6384-6391.

89. Kleining H, Reichenbach H (1977) Carotenoid glucosides and menaquinones from the gliding bacterium *Herptosiphon giganteus* Hp. a2. Arch Microbiol 112: 307-310.

90. Takaichi S, Moaka T, Yamada M, Matsuura K, Haikawa Y, et al. (2001) Absence of carotenes and presence of a tertiary methoxy group in a carotenoid from a thermophilic filamentous photosynthetic bacterium *Roseiflexus castenholzii*. Plant Cell Physiol 42: 1355-1362.

91. Xu Z, Tian B, Sun Z, Lin J, Hua Y (2007) Identification and functional analysis of a phytoene desaturase gene from the extremely radioresistant bacterium *Deinococcus radiodurans*. Microbiology 153: 1642-1652.

92. Tian B, Sun Z, Xu Z, Shen S, Wang H, et al. (2008) Carotenoid 3',4'-desaturase is involved in carotenoid biosynthesis in the radioresistant bacterium *Deinococcus radiodurans*. Microbiology 154: 3697-3706.

93. Blasco F, Kauffmann L, Schmid RD (2004) CYP175A1 from *Thermus thermophilus* HB27, the first β-carotene hydroxylase of the P450 superfamily. Appl Microbiol Biotechnol 64: 671-674.

94. Tabata K, Ishida S, Nakahara T, Hoshino T (1994) A carotenogenic gene cluster exists on a large plasmid in *Thermus thermophilus*. FEBS Lett 341: 251-255.

95. Hemmi H, Ikejiri S, Nakayama T, Nishino T (2003) Fusion-type lycopene β-cyclase from a thermoacidophilic archaeon *Sulfolobus solfataricus*. Biochem Biophys Res Commun 305: 586-591.

96. Daub ME, Payne GA (1989) The role of carotenoids in resistance of fungi to cercosporin. Phytopathology 79: 180-185.

97. Ehrenshaft M, Daub ME (1994) Isolation, sequence, and characterization of the *Cercospora nicotianae* phytoene dehydrogenase gene. Appl Environ Microbiol 60: 2766-2771.

98. Avalos J, Casadesús J, Cerdá-Olmedo E (1985) *Gibberella fujikuroi* mutants obtained with UV radiation and *N-*methyl-*N'-*nitro-*N*-nitrosoguanidine. Appl Environ Microbiol 49: 187-191.

99. Linnemannstöns P, Prado MM, Fernández-Martín R, Tudzynski B, Avalos J (2002) A carotenoid biosynthesis gene cluster in *Fusariuim fujikuroi*: the genes *carB* and *carRA*. Mol Genet Genomics 267: 593-602.

100. Prado-Cabrero A, Estrada AF, Al-Babili S, Avalos J (2007) Identification and biochemical characterization of a novel carotenoid oxygenase: elucidation of the cleavage step in the *Fusarium* carotenoid pathway. Mol Microbiol 64: 448-460.

101. Fraser PD, Ruiz-Hidalgo MJ, Lopez-Matas MA, Alvarez MI, Eslava AP, et al. (1996) Carotenoid biosynthesis in wild type and mutant strains of *Mucor circinelloides*. Biochim Biophys Acta 1289: 203-208.

102. Velayos A, Eslava AP, Iturriaga EA (2000) A bifunctional enzyme with lycopene cyclase and phytoene synthase activities is encoded by the *carRP* gene of *Mucor circinelloides*. Eur J Biochem 267: 5509-5519.

103. Estrada AF, Youssar L, Scherzinger D, Al-Babili S, Avalos J (2008) The *ylo-1* gene encodes an aldehyde dehydrogenase responsible for the last reaction in the *Neurospora* carotenoid pathway. Mol Microbiol 69: 1207-1220.

104. Schmidhauser TJ, Lauter FR, Russo VEA, Yanofsky C (1990) Cloning, sequence, and photoregulation of *al-1*, a carotenoid biosynthetic gene of *Neurospora crassa*. Mol Cell Biol 10: 5064-5070.

105. Schmidhauser TJ, Lauter F-R, Schumacher M, Zhou W, Russo VEA, et al. (1994) Characterization of *al-2*, the phytoene synthase gene of *Neurospora crassa*. J Biol Chem 269: 12060-12066.

106. Saelices L, Youssar L, Holdermann I, Al-Babili S, Avalos J (2007) Identification of the gene responsible for torulene cleavage in the *Neurospora* carotenoid pathway. Mol Genet Genomics 278: 527-537.

107. Shlomai P, Ben-Amotz A, Margalith P (1991) Production of carotene stereoisomers by *Phycomyces blakesleeanus*. Appl Microbiol Biotechnol 34: 458-462.

108. Ruiz-Hidalgo MJ, Benito EP, Sandmann G, Eslava AP (1997) The phytoene dehydrogenase gene of *Phycomyces*: regulation of its expression by blue light and vitamin A. Mol Gen Genet 253: 734-744.

109. Arrach N, Fernández-Martín R, Cardá-Olmedo E, Avalos J (2001) A single gene for lycopene cyclase, phytoene synthase, and regulation of carotene biosynthesis in *Phycomyces*. Proc Nat Acad Sci USA 98: 1687-1692.

110. Andrews AG, Starr MP (1976) (3R,3R')-Astaxanthin from the yeast *Phaffia rhodozyma*. Phytochemistry 15: 1009-1011.

111. Verdoes JC, Krubasik P, Sandmann G, van Ooyen AJJ (1999) Isolation and functional characterisation of a novel type of carotenoid biosynthetic gene from *Xanthophyllomyces dendrorhous*. Mol Gen Genet 262: 453-461.

112. Verdoes JC, Misawa N, van Ooyen AJJ (1999) Cloning and characterization of the astaxanthin biosynthetic gene encoding phytoene desaturase of *Xanthophyllomyces dendrorhous*. Biotechnol Bioeng 63: 750-755.

113. Cunningham Jr. FX, Lee H, Gantt E (2007) Carotenoid biosynthesis in the primitive red alga *Cyanidioschyzon merolae*. Eukaryotic Cell 6: 533-545.

114. Ramos A, Coesel S, Marques A, Rodrigues M, Baumgartner A, et al. (2008) Isolation and characterization of a stress-inducible *Dunaliella salina Lcy-β* gene encoding a functional lycopene β-cyclase. Appl Microbiol Biotechnol 79: 819-828.

115. Mil'ko ES (1963) Effect of various environmental factors on pigment production in the alga *Dunaliella salina*. Mikrobiolgyia 32: 299-307.

116. Kajiwara S, Kakizono T, Saito T, Kondo K, Ohtani T, et al. (1995) Isolation and functional identification of a novel cDNA for astaxanthin biosynthesis from *Haematococcus pluvialis*, and astaxanthin synthesis in *Escherichia coli*. Plant Mol Biol 29: 343-352.

117. Linden H (1999) Carotenoid hydroxylase from *Haematococcus pluvialus*: cDNA sequence, regulation and functional complementation. Biochim Biophys Acta 1446: 203-212.

118. Lotan T, Hirschberg J (1995) Cloning and expression in *Escherichia coli* of the gene encoding β-C-4-oxygenase, that converst β-carotene to the ketocarotenoid canthaxanthin in *Haematococcus pluvialis*. FEBS Lett 364: 125-128.

119. Steinbrenner J, Linden H (2001) Regulation of two carotenoid biosynthesis genes coding for phytoene synthase and carotenoid hydroxylase during stress-induced astaxanthin formation in the green alga *Haematococcus pluvialis*. Plant Physiol 125: 810-817.
