# Supplementary material for: Phylogenetic and Evolutionary Patterns in Microbial Carotenoid Biosynthesis Are Revealed by Comparative Genomics
Source: PLoS One. 2010 Jun 22;5(6):e11257. doi: 10.1371/journal.pone.0011257 (PMC2889829; doi:10.1371/journal.pone.0011257)
Supplement: Table S2 — Start and end amino acids for used in this study for carotenoid biosynthesis fusion proteins. (0.05 MB DOC) [file pone.0011257.s002.doc]

Table S2. Start and end amino acids for used in this study for carotenoid biosynthesis fusion proteins.

| Organism | Protein | CrtB | | CrtY | |
| --- | --- | --- | --- | --- | --- |
|  |  | Start | End | Start | End |
| *Aspergillus niger* | CrtBYcd | 275 | 582 | 1 | 271 |
| *Aspergillus oryzae* RIB40 | CrtBYcd | 252 | 587 | 1 | 246 |
| *Gibberella fujikuroi* IMI58289 | CrtBYcd (CarRA) | 283 | 612 | 1 | 277 |
| *Gibberella zeae* PH-1 | CrtBYcd | 251 | 581 | 1 | 246 |
| *Mucor circinelloides* | CrtBYcd (CarRP) | 257 | 614 | 1 | 249 |
| *Neurospora crassa* | CrtBYcd (AL-2) | 256 | 595 | 1 | 251 |
| *Phaeosphaeria nodorum* SN15 | CrtBYcd | 258 | 585 | 1 | 253 |
| *Phycomyces blakesleeanus* | CrtBYcd (CarRA) | 254 | 602 | 1 | 248 |
| *Podospora anserina* | CrtBYcd | 257 | 593 | 1 | 252 |
| *Ustilago maydis* 521 | CrtBYcd | 283 | 690 | 1 | 265 |
| *Xanthophyllomyces dendrorhous* | CrtBYcd (CrtYB) | 269 | 673 | 1 | 261 |
|  |  | CrtB | | CrtI | |
|  |  | Start | End | Start | End |
| *Clavibacter michiganensis michiganensis* NCPPB 382 | CrtBI | 1 | 302 | 350 | 881 |
| Uncultured Marine Bacterium HF10_49E08 | CrtBI | 467 | 764 | 1 | 466 |
