# Supplementary material for: Phylogenetic and Evolutionary Patterns in Microbial Carotenoid Biosynthesis Are Revealed by Comparative Genomics
Source: PLoS One. 2010 Jun 22;5(6):e11257. doi: 10.1371/journal.pone.0011257 (PMC2889829; doi:10.1371/journal.pone.0011257)
Supplement: Table S3 — Known microbial carotenoid biosynthetic proteins used for in silico carotenoid biosynthetic pathway reconstruction, their synonyms and biochemical functions. (0.08 MB DOC) [file pone.0011257.s003.doc]

Table S3. Known microbial carotenoid biosynthetic proteins used for *in silico* carotenoid biosynthetic pathway reconstruction, their synonyms and biochemical functions.

| Protein Name | Synonyms*^a^* | E.C. Number*^b^* | Biochemical Function |
| --- | --- | --- | --- |
| Ald | - | - | 4,4′-Diapolycopene-4,4’-dial oxidase |
| CAO-2 | - | - | 3′,4′-Didehydrolycopene or Toulene (3′,4′-didehydro-γ-carotene) 3′,4′-oxidase |
| CHYB | - | 1.14.13.- | β-Carotene 3(,3′)-(di)hydroxylase |
| CHYE | - | 1.14.13.- | ε-Carotene 3(,3′)-(di)hydroxylase |
| CrtA | - | 1.-.-.- | 1’-Methoxy linear xanthophyll 2’-ketolase or -hydroxylase |
| CrtB | PSY, CarA*^c^*, CarP*^c^*, AL-2 | 2.5.1.32 | Phytoene synthase |
| CrtC | - | - | Lycopene 1-hydroxylase, γ-carotene 1′-hydroxylase |
| CrtD | - | 1.14.99.- | 1,2-Dihydrolycopene-3,4-desaturase, 1′,2′-dihydro- γ-carotene-3′,4′-desaturase |
| CrtEb | LitC*^d^* | - | Lycopene prenyl transferase |
| CrtF | - | 2.1.1.- | Linear xanthophyll methyltransferase |
| CrtG | - | - | β-Carotene 2(,2′)-(di)hydroxylase |
| CrtH | CRTISO | 5.-.-.- | 7,9,7′,9′-*cis*-lycopene isomerase |
| CrtI | CarB, AL-1 | 1.14.99.- | Phytoene desaturase |
| CrtL | CrtLb, LYCB | 1.14.-.- | Lycopene β-mono- or β-bicyclase |
| CrtLe | LYCE | 1.14.-.- | Lycopene ε-bicyclase |
| CrtM | - | 2.5.1.- | 4,4’-Diapophytoene synthase |
| CrtN | - | 1.14.99.- | 4,4’-Diapophytoene desaturase |
| CrtNb | CrtP | 1.-.-.- | 4,4’-Diaponeurosporene or 4,4′-Diapolycopene oxidase |
| CrtO | - | - | β-Carotene 4(,4′)-(di)ketolase |
| “CrtOat” | - | 2.3.1.- | 4,4’-Diaponeurosporen-4-oic acid glycosyl transferase |
| CrtP | PDS | 1.14.99.- | Phytoene desaturase |
| CrtQ | ZDS | 1.14.99.30 | ζ-Carotene desaturase |
| “CrtQgt” | - | 2.4.1 | 4,4′-Diaponeurosporen-4-oic acid glycoside acyl transferase |
| CrtR | - | 1.14.13.- | β-Carotene 3(,3′)-(di)hydroxylase |
| CrtU | - | - | β-Carotene φ-desaturase |
| CrtW | BKT | - | β-Carotene 4(,4′)-(di)ketolase |
| CrtX | - | - | Zeaxanthin glycosyl transferase |
| CrtY | CrtYm | 1.14.-.- | Lycopene β-mono- or β-bicyclase |
| CrtYcd*^e^* | CarR*^c^* | - | Lycopene β-mono- or β-bicyclase |
| CrtYef | - | - | Flavuxanthin (linear C50 carotenoid) ε-bicyclase |
| CrtZ | - | 1.14.13.- | β-Carotene 3(,3′)-(di)hydroxylase |
| CruA | - | - | Lycopene β-bicyclase |
| CruB | - | - | γ-Carotene β-monocyclase |
| CruC | - | - | 1′-Hydroxylchlorobactene glycosyl transferase |
| CruD | - | - | 1′-Hydroxylchlorobactene glycoside acyl transferase |
| CruE | - | - | β-Carotene χ-desaturase |
| CruF | - | - | γ-Carotene 1′-hydroxylase |
| CruG | - | - | Myxol or ketomyxol glycotransferase |
| CruH | - | - | Renierapurpurin (χ,χ-carotene) dicarboxylase |
| CruP | - | - | Lycopene β-monocyclase |
| CYP175A1 | - | - | β-Carotene 3(,3′)-(di)hydroxylase |
| LitAB*^d^* | - | - | Flavuxanthin (linear C50 carotenoid) β-bicyclase |
| ORF10 | - | - | Isorenieratene (φ,φ-carotene) 3(,3′)-(di)hydroxylase |
| VDE | - | 1.10.99.3 | Violaxanthin (diepoxyzeaxanthin) de-epoxidase |
| YLO-1 | - | - | Apo-4′-lycopenal or β-apo-4′-carotenal oxidase |
| ZEP | - | 1.14.13.90 | Zeaxanthin epoxidase |

*^a^*Synonyms beginning with “AL” refer to *Neurospora crassa*; synonyms beginning with “Car” refer to most other fungi; CrtP in *Staphylococcus aureus* is synonymous with CrtNb; all synonyms in block capital letters refer to photosynthetic eukaryotes

*^b^*E. C. numbers (where available) were obtained from Kyoto Encyclopaedia of Genes and Genomes pathway map00906, last updated February 24, 2009

*^c^*CarAP and CarPR exist as heterodimers

*^d^*LitBC exist in *Dietzia* sp. CQ4 as a heterodimer, although separated homologs exist in other organisms

*^e^*In some, but not all, organisms CrtYcd exists as a heterodimer
