# Supplementary figures and images for: Phylogenetic and Evolutionary Patterns in Microbial Carotenoid Biosynthesis Are Revealed by Comparative Genomics
Source: PLoS One. 2010 Jun 22;5(6):e11257. doi: 10.1371/journal.pone.0011257 (PMC2889829; doi:10.1371/journal.pone.0011257)

A
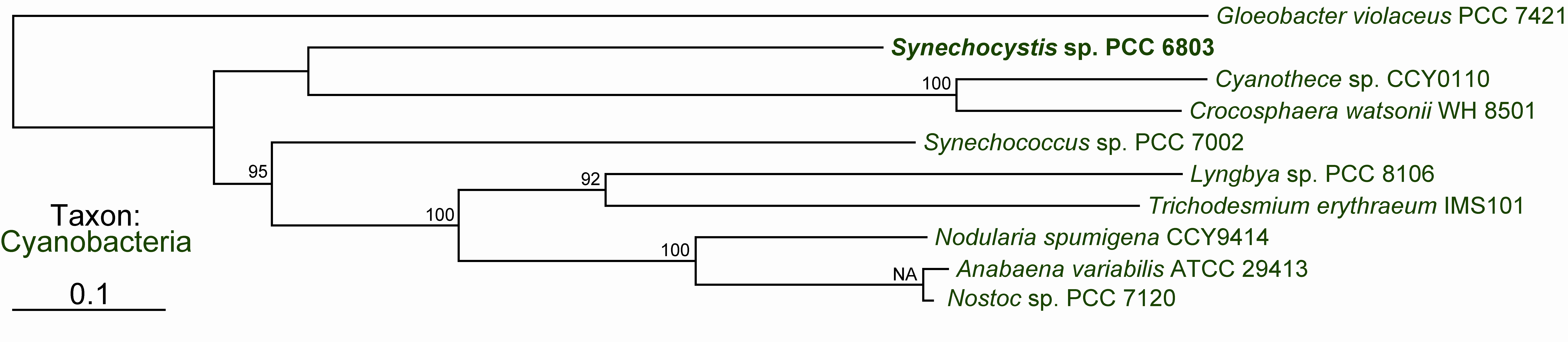


B
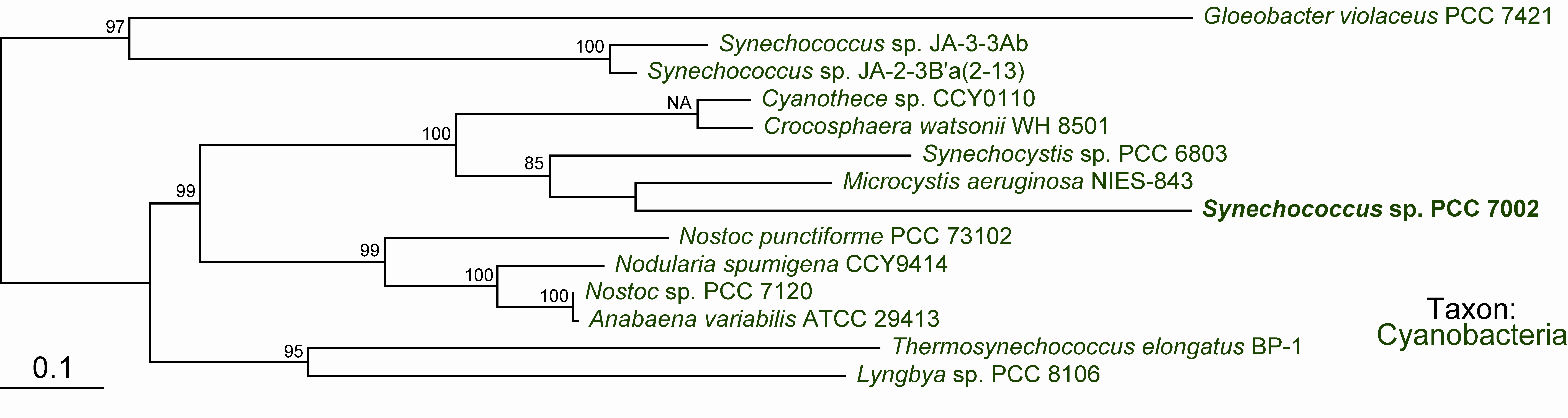


C
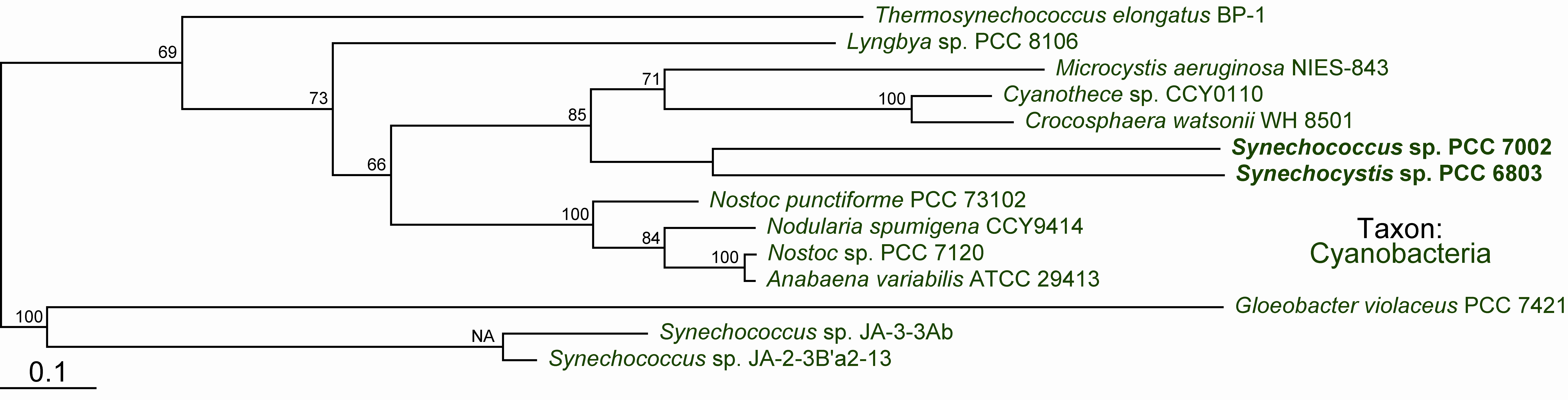


D
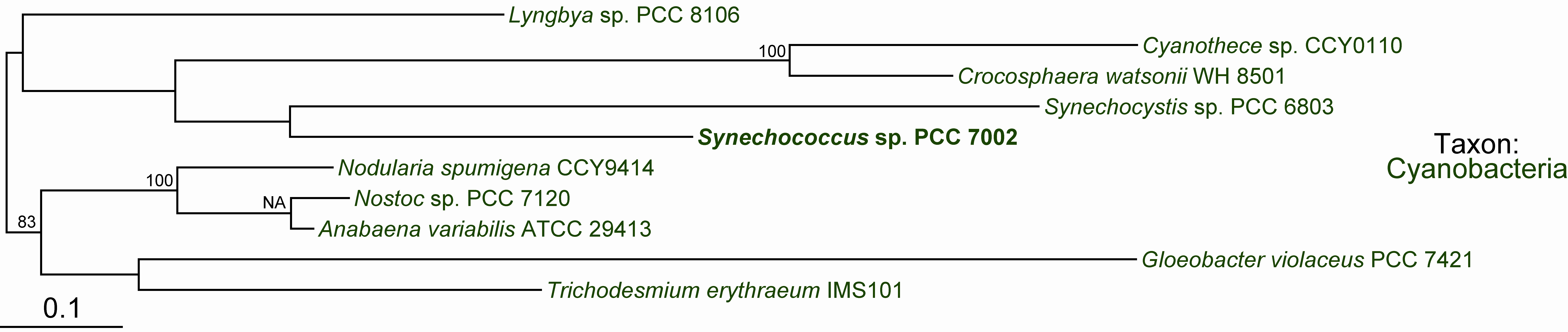

Supplement: Figure S13 — Phylogenetic trees of (A) CruE, (B) CruF, (C) CruG and (D) CruH protein sequences constructed using RAxML. Bootstrap values ≥60% are indicated as a percentage of the automatically determined number of replicates determined using the CIPRES web portal. Sequences with genetically or biochemically demonstrated functions are bolded. Carotenoids typical of each lineage are indicated to the right of each clade, with exceptions indicated by asterisks. The scale bar represents 10% sequence divergence. The trees shown are rooted to their midpoint to maximise the clarity of intraclade relationships. NA indicates the ML basal node for which no bootstrap value was given. (2.54 MB DOC) [file pone.0011257.s016.doc]

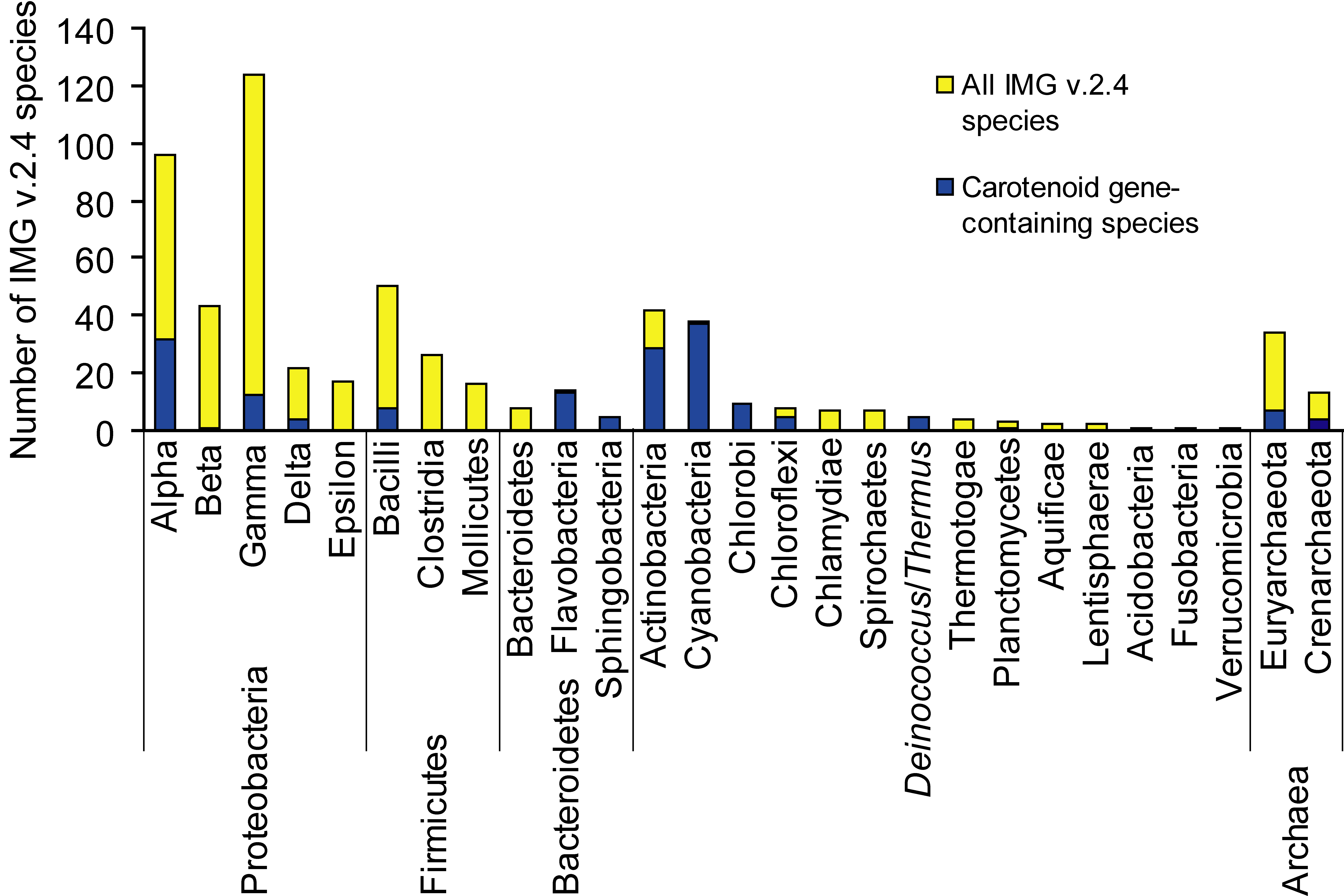

Supplement: Figure S16 — Distribution of carotenoid biosynthetic pathways (as inferred from Supplementary Table S1) in genome sequences of the IMG database, version 2.4. Except Cyanobacteria, each species was considered only once despite the presence of multiple strains. Because incomplete genomes were included this analysis represents an underestimate. (1.72 MB TIF) [file pone.0011257.s019.tif]
