# Supplementary material for: Phylogenetic and Evolutionary Patterns in Microbial Carotenoid Biosynthesis Are Revealed by Comparative Genomics
Source: PLoS One. 2010 Jun 22;5(6):e11257. doi: 10.1371/journal.pone.0011257 (PMC2889829; doi:10.1371/journal.pone.0011257)
Supplement: Figure S15 — Pairwise dn/ds values for: (A) C40 carotenoid-producing Actinobacteria crtYcd; (B) C50 carotenoid-producing Actinobacteria crtYef and myxobacterial crtB (C), crtC (D), crtD (E) and crtI (F). Matrices are one-sided, with cells of the opposite side filled with a dash. Bolded values are those highlighted in the text. In some cases a pairwise comparison of two sequences otherwise determined to have a high dn/ds values yielded an unexpectedly low dn/ds value; these ratios are iticized. NC indicates comparisons for which MEGA 4.0 could not calculate ds value. (0.08 MB DOC) [file pone.0011257.s018.doc]

*A. C_40_ Carotenoid-producing Actinobacteria crtYcd*

|  | *Frankia* sp. CcI3 | *Frankia* *alni* ACN14a | *Frankia* sp. EAN1pec | *Mycobacterium* *aurum* A+ | *Mycobacterium* *avium* subsp. *paratuberculosis* K-10 | *Mycobacterium* sp. MCS |
| --- | --- | --- | --- | --- | --- | --- |
| *Frankia alni* ACN14a | **1.523022** | - | - | - | - | - |
| *Frankia* sp. EAN1pec | 0.196231 | **1.866216** | - | - | - | - |
| *Mycobacterium aurum* A+ | **1.668994** | *0.855869* | **2.099379** | - | - | - |
| *Mycobacterium avium* subsp. *paratuberculosis* K-10 | 0.643956 | **1.522868** | 0.816431 | **2.123245** | - | - |
| *Mycobacterium* sp. MCS | 0.747638 | **2.198642** | 0.861472 | **1.596491** | 0.414443 | - |
| *Mycobacterium ulcerans* Agy99 | 0.501406 | **1.392157** | 0.559184 | **1.485261** | 0.23934 | 0.308671 |

B. C_50_ Carotenoid-producing Actinobacteria *crtYef*

|  | *Corynebacterium efficiens* YS-314 | *Corynebacterium glutamicum* ATCC 13032 | *Clavibacter michiganensis* subsp. *michiganensis* NCPPB 382 | Marine Actinobacterium PHSC20C1 | *Leifsonia xyli* subsp. *xyli* str. CTCB07 |
| --- | --- | --- | --- | --- | --- |
| *Corynebacterium glutamicum* ATCC 13032 | NC | - | - | - | - |
| *Clavibacter michiganensis* subsp. *michiganensis* NCPPB 382 | 0.810289 | NC | - | - | - |
| Marine Actinobacterium PHSC20C1 | d_s_ > 1.5 | NC | 0.818693 | - | - |
| *Leifsonia xyli* subsp. *xyli* str. CTCB07 | 0.657615 | NC | 0.44763 | 0.948099 | - |
| *Dietzia* sp. CQ4 | **1.955801** | NC | **1.998487** | d_s_ > 1.5 | **1.770889** |

C. Myxobacteria *crtB*

|  | *Sorangium cellulosum* 'So ce 56' | *Myxococcus xanthus* DK 1622 | *Stigmatella aurantiaca* DW4/3-1 |
| --- | --- | --- | --- |
| *Myxococcus xanthus* DK 1622 | **1.317324** | - | - |
| *Stigmatella aurantiaca* DW4/3-1 | **1.012788** | *0.301676* | - |
| *Plesiocystis pacifica* SIR-1 | 0.471429 | **1.171018** | **0.946352** |

*D. Myxobacteria* crtC

|  | *Sorangium cellulosum* 'So ce 56' | *Myxococcus xanthus* DK 1622 | *Stigmatella aurantiaca* DW4/3-1 |
| --- | --- | --- | --- |
| *Myxococcus xanthus* DK 1622 | **1.132099** | - | - |
| *Stigmatella aurantiaca* DW4/3-1 | **1.411848** | *0.392027* | - |
| *Plesiocystis pacifica* SIR-1 | 0.855153 | **1.087117** | **1.167082** |

*E. Myxobacteria* crtD

|  | *Sorangium cellulosum* 'So ce 56' | *Myxococcus xanthus* DK 1622 | *Plesiocystis pacifica*  SIR-1 |
| --- | --- | --- | --- |
| *Myxococcus xanthus* DK 1622 | **1.080882** | - | - |
| *Plesiocystis pacifica* SIR-1 | 0.59375 | **1.056098** | - |
| *Stigmatella aurantiaca* DW4/3-1 | **1.009467** | *0.323124* | **1.046838** |

*F. Myxobacteria* crtI

|  | *Sorangium cellulosum*  'So ce 56' | *Myxococcus xanthus* DK 1622 (*crtI*-like) | *Plesiocystis pacifica* SIR-1 | *Myxococcus xanthus* DK 1622 (*crtI*) | *Myxococcus xanthus* DK 1622 (*crtIb*) | *Stigmatella aurantiaca* DW4/3-1 (*crtIb*) |
| --- | --- | --- | --- | --- | --- | --- |
| *Myxococcus xanthus* DK 1622 (*crtI*-like) | 0.432967 | - | - | - | - | - |
| *Plesiocystis pacifica* SIR-1 | 0.392694 | 0.412391 | - | - | - | - |
| *Myxococcus xanthus* DK 1622 (*crtI*) | **1.041018** | **0.985788** | **1.055707** | - | - | - |
| *Myxococcus xanthus* DK 1622 (*crtIb*) | **1.166667** | **0.911435** | **1.092288** | **1.025526** | - | - |
| *Stigmatella aurantiaca* DW4/3-1 (*crtIb*) | **1.184828** | **1.00618** | **1.013746** | **0.969697** | *0.296296* | - |
| *Stigmatella aurantiaca* DW4/3-1 (*crtI*) | **1.139104** | **0.909305** | **1.073816** | *0.26087* | **1.117293** | **1.098431** |
