# Supplementary material for: Phylogenetic and Evolutionary Patterns in Microbial Carotenoid Biosynthesis Are Revealed by Comparative Genomics
Source: PLoS One. 2010 Jun 22;5(6):e11257. doi: 10.1371/journal.pone.0011257 (PMC2889829; doi:10.1371/journal.pone.0011257)
Supplement: Supplementary file 6 [file pone.0011257.s004.doc]

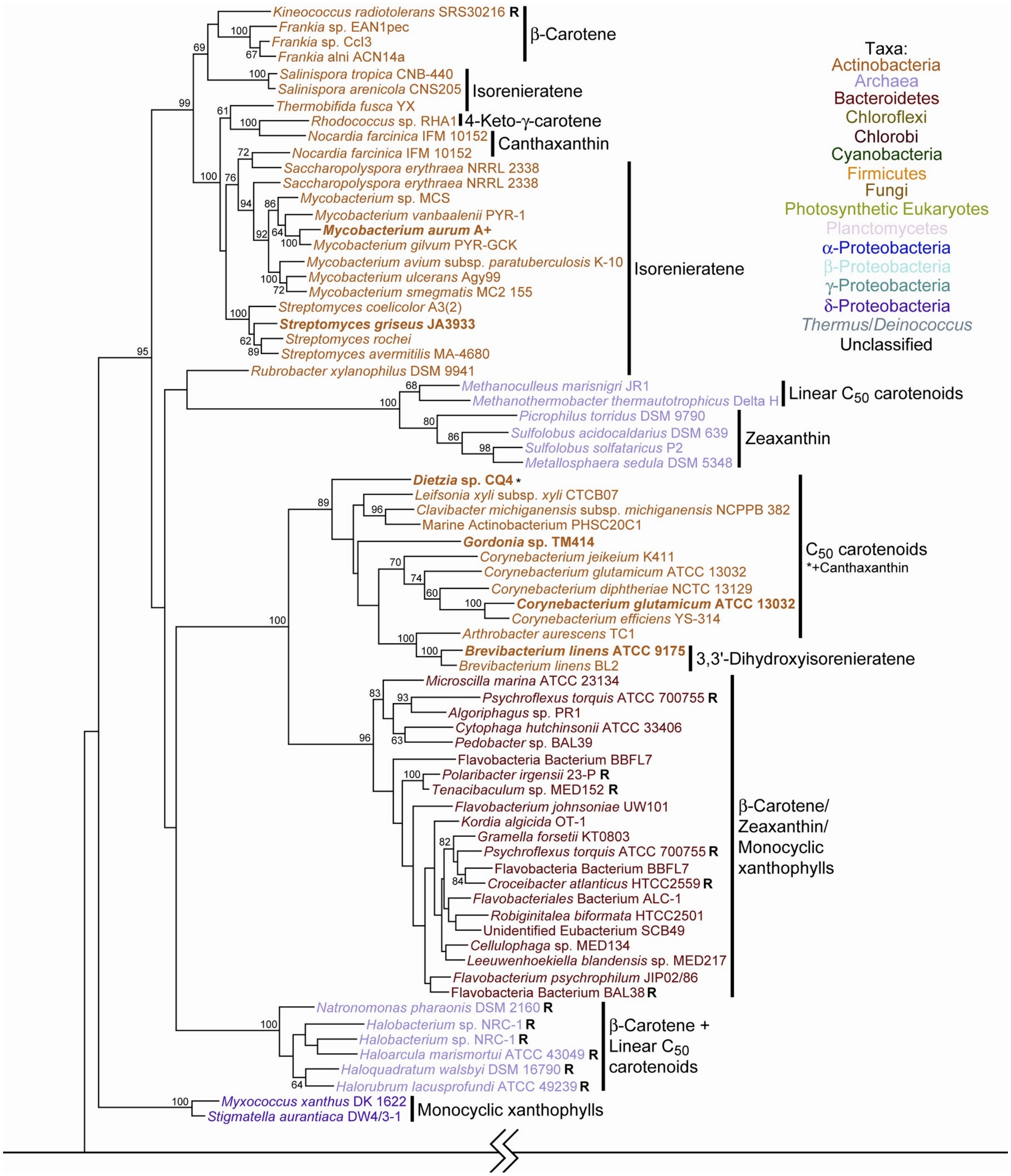

Kineococcus radiotolerans SRS30216 R
Frankia sp. EAN1pec
Frankia sp. Ccl3
Frankia alni ACN14a
β-Carotene
Salinispora tropica CNB-440
Salinispora arenicola CNS205
Isorenieratene
Thermobifida fusca YX
Rhodococcus sp. RHA1
4-Keto-γ-carotene
Nocardia farcinica IFM 10152
Canthaxanthin
Nocardia farcinica IFM 10152
Saccharopolyspora erythraea NRRL 2338
Saccharopolyspora erythraea NRRL 2338
Mycobacterium sp. MCS
Mycobacterium vanbaalenii PYR-1
Mycobacterium aurum A+
Mycobacterium gilvum PYR-GCK
Mycobacterium avium subsp. paratuberculosis K-10
Mycobacterium ulcerans Agy99
Mycobacterium smegmatis MC2 155
Streptomyces coelicolor A3(2)
Streptomyces griseus JA3933
Streptomyces rochei
Streptomyces avermitilis MA-4680
Isorenieratene
Rubrobacter xylanophilus DSM 9941
Methanoculleus marisnigri JR1
Methanothermobacter thermautotrophicus Delta H
Linear C50 carotenoids
Picrophilus torridus DSM 9790
Sulfolobus acidocaldarius DSM 639
Sulfolobus solfataricus P2
Metallosphaera sedula DSM 5348
Zeaxanthin
Dietzia sp. CQ4 *
Leifsonia xyli subsp. xyli CTCB07
Clavibacter michiganensis subsp. michiganensis NCPPB 382
Marine Actinobacterium PHSC20C1
Gordonia sp. TM414
Corynebacterium jeikeium K411
Corynebacterium glutamicum ATCC 13032
Corynebacterium diphtheriae NCTC 13129
Corynebacterium glutamicum ATCC 13032
Corynebacterium efficiens YS-314
Arthrobacter aurescens TC1
C50 carotenoids
*+Canthaxanthin
Brevibacterium linens ATCC 9175
Brevibacterium linens BL2
3,3'-Dihydroxyisorenieratene
Microscilla marina ATCC 23134
Psychroflexus torquis ATCC 700755 R
Algoriphagus sp. PR1
Cytophaga hutchinsonii ATCC 33406
Pedobacter sp. BAL39
Flavobacteria Bacterium BBFL7
Polaribacter irgensii 23-P R
Tenacibaculum sp. MED152 R
Flavobacterium johnsoniae UW101
Kordia algicida OT-1
Gramella forsetii KT0803
Psychroflexus torquis ATCC 700755 R
Flavobacteria Bacterium BBFL7
Croceibacter atlanticus HTCC2559 R
Flavobacteriales Bacterium ALC-1
Robiginitalea biformata HTCC2501
Unidentified Eubacterium SCB49
Cellulophaga sp. MED134
Leeuwenhoekiella blandensis sp. MED217
Flavobacterium psychrophilum JIP02/86
Flavobacteria Bacterium BAL38 R
β-Carotene/
Zeaxanthin/
Monocyclic
xanthophylls
Natronomonas pharaonis DSM 2160 R
Halobacterium sp. NRC-1 R
Halobacterium sp. NRC-1 R
Haloarcula marismortui ATCC 43049 R
Haloquadratum walsbyi DSM 16790 R
Halorubrum lacusprofundi ATCC 49239 R
β-Carotene +
Linear C50
carotenoids
Myxococcus xanthus DK 1622
Stigmatella aurantiaca DW4/3-1
Monocyclic xanthophylls
Taxa:
Actinobacteria
Archaea
Bacteroidetes
Chloroflexi
Chlorobi
Cyanobacteria
Firmicutes
Fungi
Photosynthetic Eukaryotes
Planctomycetes
α-Proteobacteria
β-Proteobacteria
γ-Proteobacteria
δ-Proteobacteria
Thermus/Deinococcus
Unclassified

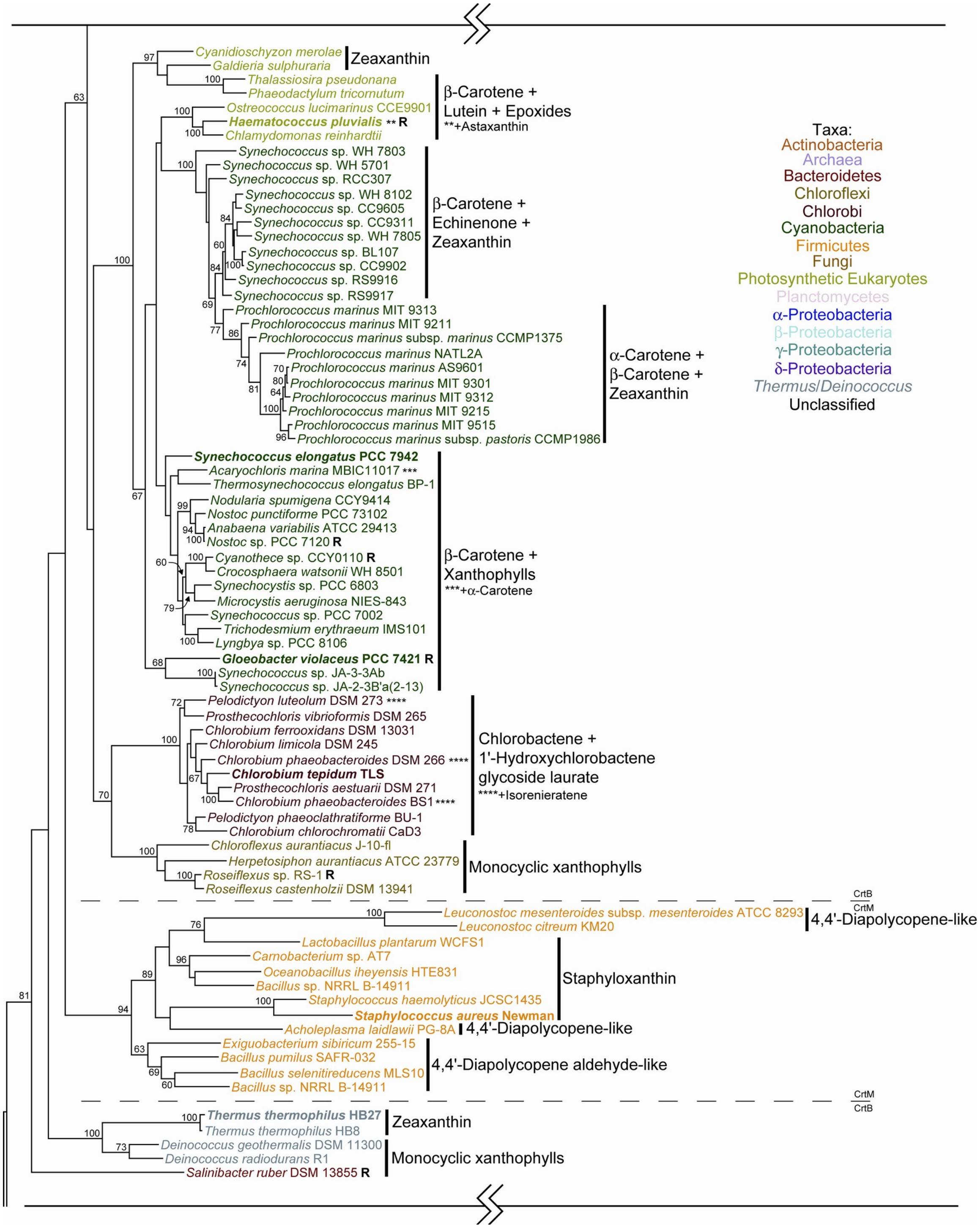

Cyanidioschyzon merolae
Galdieria sulphuraria
Zeaxanthin
Thalassiosira pseudonana
Phaeodactylum tricornutum
Ostreococcus lucimarinus CCE9901
Haematococcus pluvialis ** R
Chlamydomonas reinhardtii
β-Carotene + Lutein + Epoxides
**+Astaxanthin
Synechococcus sp. WH 7803
Synechococcus sp. WH 5701
Synechococcus sp. RCC307
Synechococcus sp. WH 8102
Synechococcus sp. CC9605
Synechococcus sp. CC9311
Synechococcus sp. WH 7805
Synechococcus sp. BL107
Synechococcus sp. CC9902
Synechococcus sp. RS9916
Synechococcus sp. RS9917
β-Carotene + Echinenone + Zeaxanthin
Prochlorococcus marinus MIT 9313
Prochlorococcus marinus MIT 9211
Prochlorococcus marinus subsp. marinus CCMP1375
Prochlorococcus marinus NATL2A
Prochlorococcus marinus AS9601
Prochlorococcus marinus MIT 9301
Prochlorococcus marinus MIT 9312
Prochlorococcus marinus MIT 9215
Prochlorococcus marinus MIT 9515
Prochlorococcus marinus subsp. pastoris CCMP1986
α-Carotene + β-Carotene + Zeaxanthin
Synechococcus elongatus PCC 7942
Acaryochloris marina MBIC11017 ***
Thermosynechococcus elongatus BP-1
Nodularia spumigena CCY9414
Nostoc punctiforme PCC 73102
Anabaena variabilis ATCC 29413
Nostoc sp. PCC 7120 R
Cyanothece sp. CCY0110 R
Crocosphaera watsonii WH 8501
Synechocystis sp. PCC 6803
Microcystis aeruginosa NIES-843
Synechococcus sp. PCC 7002
Trichodesmium erythraeum IMS101
Lyngbya sp. PCC 8106
Gloeobacter violaceus PCC 7421 R
Synechococcus sp. JA-3-3Ab
Synechococcus sp. JA-2-3B'a(2-13)
β-Carotene + Xanthophylls
***+α-Carotene
Pelodictyon luteolum DSM 273 ****
Prosthecochloris vibrioformis DSM 265
Chlorobium ferrooxidans DSM 13031
Chlorobium limicola DSM 245
Chlorobium phaeobacteroides DSM 266 ****
Chlorobium tepidum TLS
Prosthecochloris aestuarii DSM 271
Chlorobium phaeobacteroides BS1 ****
Pelodictyon phaeoclathratiforme BU-1
Chlorobium chlorochromatii CaD3
Chlorobactene + 1'-Hydroxychlorobactene glycoside laurate
****+Isorenieratene
Chloroflexus aurantiacus J-10-fl
Herpetosiphon aurantiacus ATCC 23779
Roseiflexus sp. RS-1 R
Roseiflexus castenholzii DSM 13941
Monocyclic xanthophylls
CrtB
CrtM
Leuconostoc mesenteroides subsp. mesenteroides ATCC 8293
Leuconostoc citreum KM20
4,4'-Diapolycopene-like
Lactobacillus plantarum WCFS1
Carnobacterium sp. AT7
Oceanobacillus iheyensis HTE831
Bacillus sp. NRRL B-14911
Staphylococcus haemolyticus JCSC1435
Staphylococcus aureus Newman
Staphyloxanthin
Acholeplasma laidlawii PG-8A
4,4'-Diapolycopene-like
Exiguobacterium sibiricum 255-15
Bacillus pumilus SAFR-032
Bacillus selenitireducens MLS10
Bacillus sp. NRRL B-14911
4,4'-Diapolycopene aldehyde-like
CrtM
CrtB
Thermus thermophilus HB27
Thermus thermophilus HB8
Zeaxanthin
Deinococcus geothermalis DSM 11300
Deinococcus radiodurans R1
Salinibacter ruber DSM 13855 R
Monocyclic xanthophylls
Taxa:
Actinobacteria
Archaea
Bacteroidetes
Chloroflexi
Chlorobi
Cyanobacteria
Firmicutes
Fungi
Photosynthetic Eukaryotes
Planctomycetes
α-Proteobacteria
β-Proteobacteria
γ-Proteobacteria
δ-Proteobacteria
Thermus/Deinococcus
Unclassified

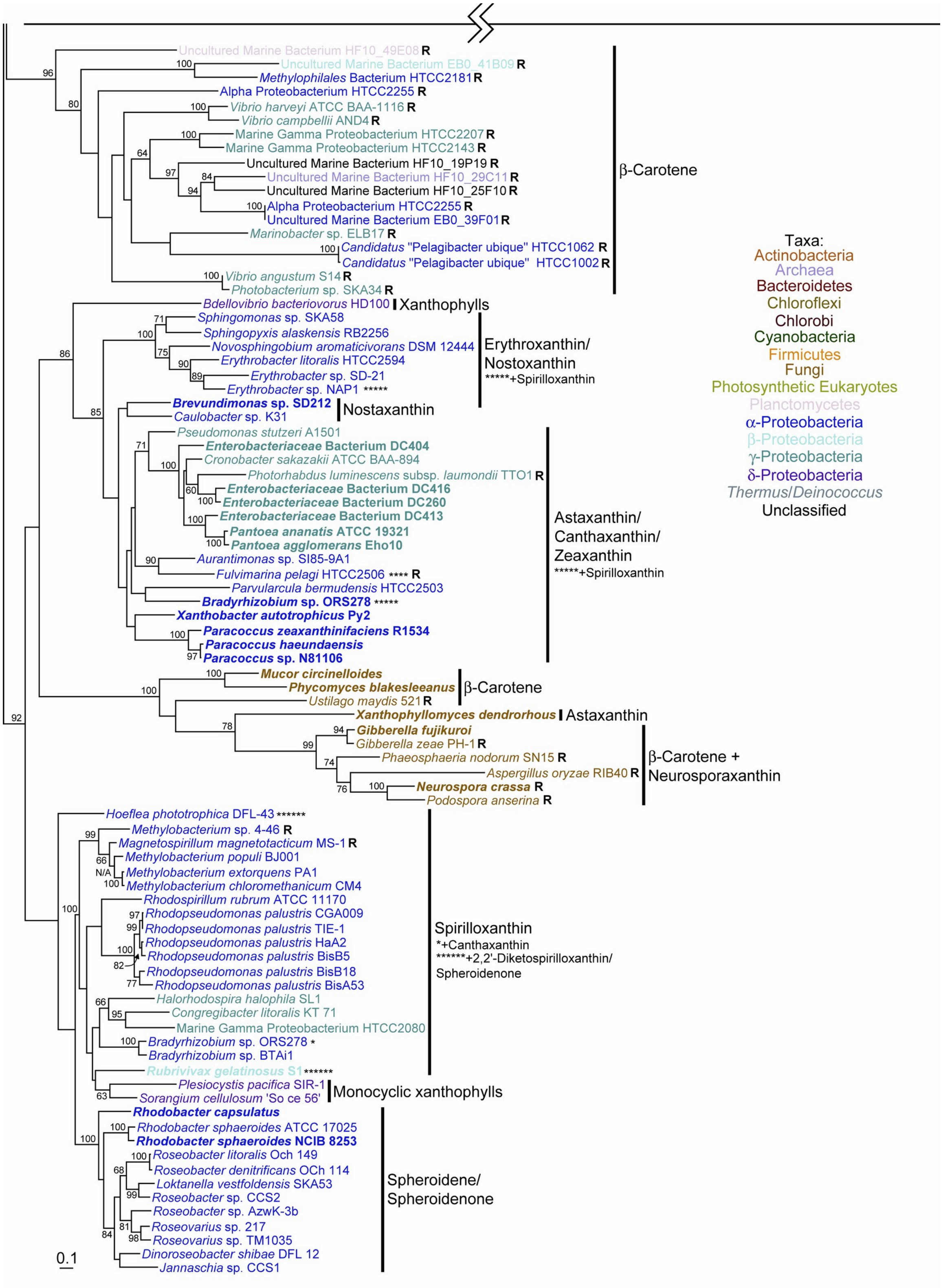

Uncultured Marine Bacterium HF10_49E08 R
Uncultured Marine Bacterium EB0_41B09 R
Methylophilales Bacterium HTCC2181 R
Alpha Proteobacterium HTCC2255 R
Vibrio harveyi ATCC BAA-1116 R
Vibrio campbellii AND4 R
Marine Gamma Proteobacterium HTCC2207 R
Marine Gamma Proteobacterium HTCC2143 R
Uncultured Marine Bacterium HF10_19P19 R
Uncultured Marine Bacterium HF10_29C11 R
Uncultured Marine Bacterium HF10_25F10 R
Alpha Proteobacterium HTCC2255 R
Uncultured Marine Bacterium EB0_39F01 R
Marinobacter sp. ELB17 R
Candidatus "Pelagibacter ubique" HTCC1062 R
Candidatus "Pelagibacter ubique" HTCC1002 R
Vibrio angustum S14 R
Photobacterium sp. SKA34 R
β-Carotene
Bdellovibrio bacteriovorus HD100
Xanthophylls
Sphingomonas sp. SKA58
Sphingopyxis alaskensis RB2256
Novosphingobium aromaticivorans DSM 12444
Erythrobacter litoralis HTCC2594
Erythrobacter sp. SD-21
Erythrobacter sp. NAP1 *****
Erythroxanthin/
Nostoxanthin
*****+Spirilloxanthin
Brevundimonas sp. SD212
Caulobacter sp. K31
Nostaxanthin
Pseudomonas stutzeri A1501
Enterobacteriaceae Bacterium DC404
Cronobacter sakazakii ATCC BAA-894
Photorhabdus luminescens subsp. laumondii TTO1 R
Enterobacteriaceae Bacterium DC416
Enterobacteriaceae Bacterium DC260
Enterobacteriaceae Bacterium DC413
Pantoea ananatis ATCC 19321
Pantoea agglomerans Eho10
Aurantimonas sp. SI85-9A1
Fulvimarina pelagi HTCC2506 **** R
Parvularcula bermudensis HTCC2503
Bradyrhizobium sp. ORS278 *****
Xanthobacter autotrophicus Py2
Paracoccus zeaxanthinifaciens R1534
Paracoccus haeundaensis
Paracoccus sp. N81106
Astaxanthin/
Canthaxanthin/
Zeaxanthin
*****+Spirilloxanthin
Mucor circinelloides
Phycomyces blakesleeanus
Ustilago maydis 521 R
β-Carotene
Xanthophyllomyces dendrorhous
Astaxanthin
Gibberella fujikuroi
Gibberella zeae PH-1 R
Phaeosphaeria nodorum SN15 R
Aspergillus oryzae RIB40 R
Neurospora crassa R
Podospora anserina R
β-Carotene +
Neurosporaxanthin
Hoeflea phototrophica DFL-43 ******
Methylobacterium sp. 4-46 R
Magnetospirillum magnetotacticum MS-1 R
Methylobacterium populi BJ001
Methylobacterium extorquens PA1
Methylobacterium chloromethanicum CM4
Rhodospirillum rubrum ATCC 11170
Rhodopseudomonas palustris CGA009
Rhodopseudomonas palustris TIE-1
Rhodopseudomonas palustris HaA2
Rhodopseudomonas palustris BisB5
Rhodopseudomonas palustris BisB18
Rhodopseudomonas palustris BisA53
Halorhodospira halophila SL1
Congregibacter litoralis KT 71
Marine Gamma Proteobacterium HTCC2080
Bradyrhizobium sp. ORS278 *
Bradyrhizobium sp. BTAi1
Rubrivivax gelatinosus S1 ******
Spirilloxanthin
*+Canthaxanthin
******+2,2'-Diketospirilloxanthin/
Spheroidenone
Plesiocystis pacifica SIR-1
Sorangium cellulosum 'So ce 56'
Monocyclic xanthophylls
Rhodobacter capsulatus
Rhodobacter sphaeroides ATCC 17025
Rhodobacter sphaeroides NCIB 8253
Roseobacter litoralis Och 149
Roseobacter denitrificans OCh 114
Loktanella vestfoldensis SKA53
Roseobacter sp. CCS2
Roseobacter sp. AzwK-3b
Roseovarius sp. 217
Roseovarius sp. TM1035
Dinoroseobacter shibae DFL 12
Jannaschia sp. CCS1
Spheroidene/
Spheroidenone
0.1
Taxa:
Actinobacteria
Archaea
Bacteroidetes
Chloroflexi
Chlorobi
Cyanobacteria
Firmicutes
Fungi
Photosynthetic Eukaryotes
Planctomycetes
α-Proteobacteria
β-Proteobacteria
γ-Proteobacteria
δ-Proteobacteria
Thermus/Deinococcus
Unclassified
