# Supplementary material for: Phylogenetic and Evolutionary Patterns in Microbial Carotenoid Biosynthesis Are Revealed by Comparative Genomics
Source: PLoS One. 2010 Jun 22;5(6):e11257. doi: 10.1371/journal.pone.0011257 (PMC2889829; doi:10.1371/journal.pone.0011257)
Supplement: Supplementary file 7 [file pone.0011257.s005.doc]

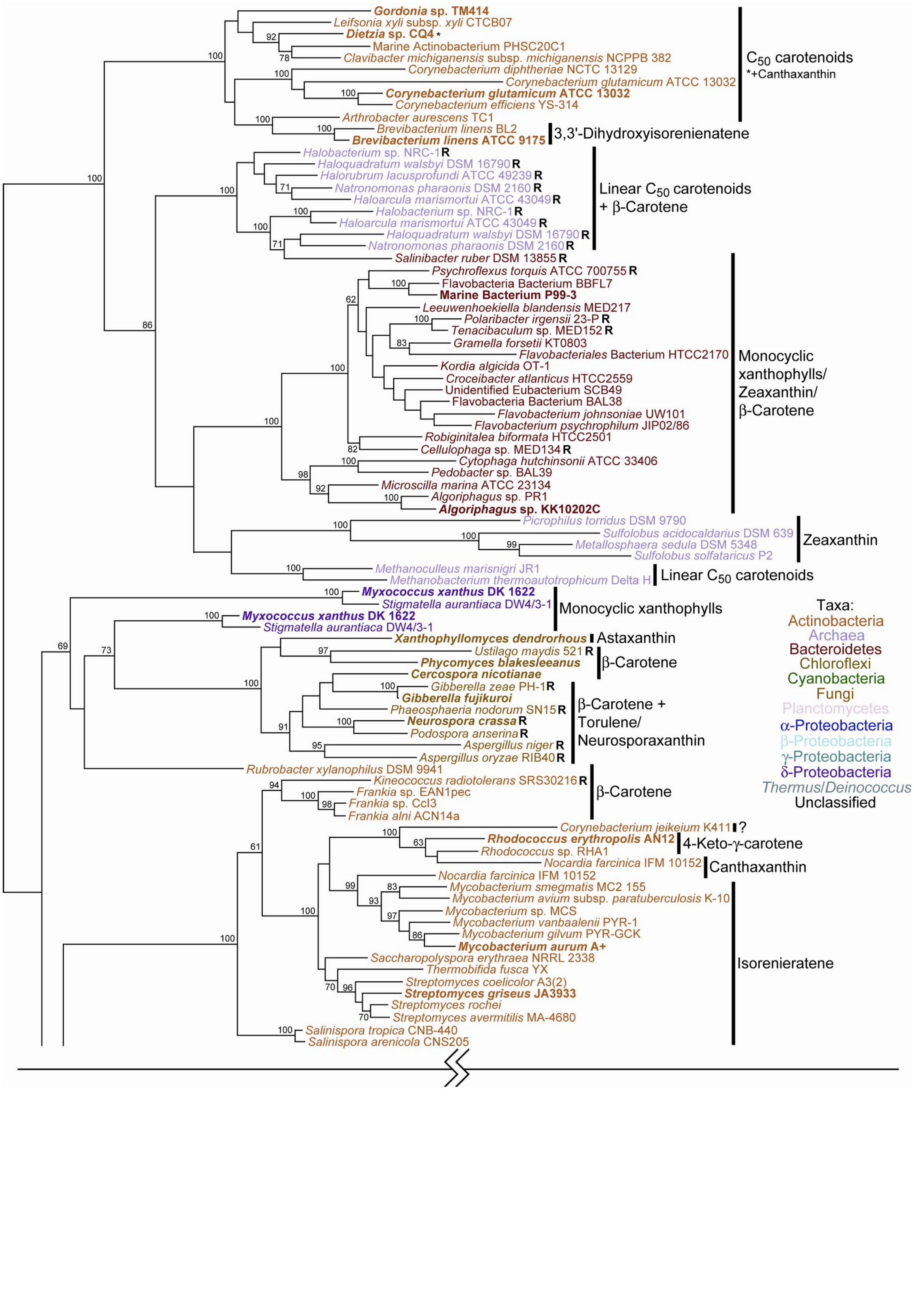

Gordonia sp. TM414
Leifsonia xyli subsp. xyli CTCB07
Dietzia sp. CQ4 *
Marine Actinobacterium PHSC20C1
Clavibacter michiganensis subsp. michiganensis NCPPB 382
Corynebacterium diphtheriae NCTC 13129
Corynebacterium glutamicum ATCC 13032
Corynebacterium glutamicum ATCC 13032
Corynebacterium efficiens YS-314
Arthrobacter aurescens TC1
Brevibacterium linens BL2
Brevibacterium linens ATCC 9175
C_{50} carotenoids
*+Canthaxanthin
3,3'-Dihydroxyisorenienatene
Halobacterium sp. NRC-1 R
Haloquadratum walsbyi DSM 16790 R
Halorubrum lacusprofundi ATCC 49239 R
Natronomonas pharaonis DSM 2160 R
Haloarcula marismortui ATCC 43049 R
Halobacterium sp. NRC-1 R
Haloarcula marismortui ATCC 43049 R
Haloquadratum walsbyi DSM 16790 R
Natronomonas pharaonis DSM 2160 R
Salinibacter ruber DSM 13855 R
Linear C_{50} carotenoids + β-Carotene
Psychroflexus torquis ATCC 700755 R
Flavobacteria Bacterium BBFL7
Marine Bacterium P99-3
Leeuwenhoekiella blandensis MED217
Polaribacter irgensii 23-P R
Tenacibaculum sp. MED152 R
Gramella forsetii KT0803
Flavobacteriales Bacterium HTCC2170
Kordia algicida OT-1
Croceibacter atlanticus HTCC2559
Unidentified Eubacterium SCB49
Flavobacteria Bacterium BAL38
Flavobacterium johnsoniae UW101
Flavobacterium psychrophilum JIP02/86
Robiginitalea biformata HTCC2501
Cellulophaga sp. MED134 R
Cytophaga hutchinsonii ATCC 33406
Pedobacter sp. BAL39
Microscilla marina ATCC 23134
Algoriphagus sp. PR1
Algoriphagus sp. KK10202C
Monocyclic xanthophylls/ Zeaxanthin/ β-Carotene
Picrophilus torridus DSM 9790
Sulfolobus acidocaldarius DSM 639
Metallosphaera sedula DSM 5348
Sulfolobus solfataricus P2
Zeaxanthin
Methanoculleus marisnigri JR1
Methanobacterium thermoautotrophicum Delta H
Linear C_{50} carotenoids
Myxococcus xanthus DK 1622
Stigmatella aurantiaca DW4/3-1
Myxococcus xanthus DK 1622
Stigmatella aurantiaca DW4/3-1
Monocyclic xanthophylls
Xanthophyllomyces dendrorhous
Astaxanthin
Ustilago maydis 521 R
Phycomyces blakesleeanus
β-Carotene
Cercospora nicotianae
Gibberella zeae PH-1 R
Gibberella fujikuroi
Phaeosphaeria nodorum SN15 R
Neurospora crassa R
Podospora anserina R
Aspergillus niger R
Aspergillus oryzae RIB40 R
β-Carotene + Torulene/ Neurosporaxanthin
Rubrobacter xylanophilus DSM 9941
Kineococcus radiotolerans SRS30216 R
Frankia sp. EAN1pec
Frankia sp. CcI3
Frankia alni ACN14a
β-Carotene
Corynebacterium jeikeium K411
?
Rhodococcus erythropolis AN12
Rhodococcus sp. RHA1
4-Keto-γ-carotene
Nocardia farcinica IFM 10152
Canthaxanthin
Nocardia farcinica IFM 10152
Mycobacterium smegmatis MC2 155
Mycobacterium avium subsp. paratuberculosis K-10
Mycobacterium sp. MCS
Mycobacterium vanbaalenii PYR-1
Mycobacterium gilvum PYR-GCK
Mycobacterium aurum A+
Saccharopolyspora erythraea NRRL 2338
Thermobifida fusca YX
Streptomyces coelicolor A3(2)
Streptomyces griseus JA3933
Streptomyces rochei
Streptomyces avermitilis MA-4680
Salinispora tropica CNB-440
Salinispora arenicola CNS205
Isorenieratene
Taxa:
Actinobacteria
Archaea
Bacteroidetes
Chloroflexi
Cyanobacteria
Fungi
Planctomycetes
α-Proteobacteria
β-Proteobacteria
γ-Proteobacteria
δ-Proteobacteria
Thermus/Deinococcus
Unclassified

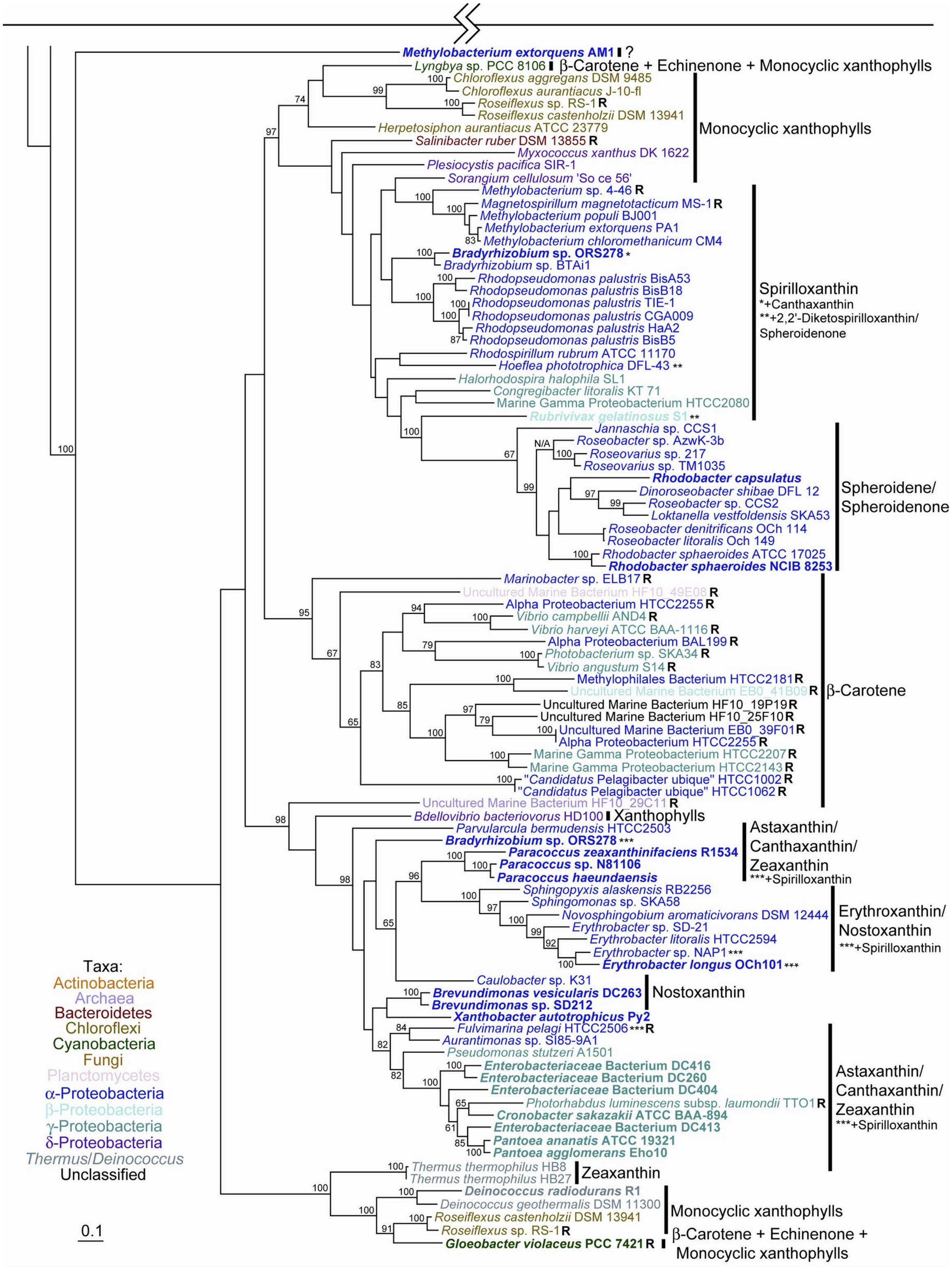

Methylobacterium extorquens AM1 ?
Lyngbya sp. PCC 8106 β-Carotene + Echinenone + Monocyclic xanthophylls
Chloroflexus aggregans DSM 9485
Chloroflexus aurantiacus J-10-fl
Roseiflexus sp. RS-1 R
Roseiflexus castenholzii DSM 13941
Herpetosiphon aurantiacus ATCC 23779
Salinibacter ruber DSM 13855 R
Myxococcus xanthus DK 1622
Plesiocystis pacifica SIR-1
Sorangium cellulosum 'So ce 56'
Monocyclic xanthophylls
Methylobacterium sp. 4-46 R
Magnetospirillum magnetotacticum MS-1 R
Methylobacterium populi BJ001
Methylobacterium extorquens PA1
Methylobacterium chloromethanicum CM4
Bradyrhizobium sp. ORS278 *
Bradyrhizobium sp. BTAi1
Rhodopseudomonas palustris BisA53
Rhodopseudomonas palustris BisB18
Rhodopseudomonas palustris TIE-1
Rhodopseudomonas palustris CGA009
Rhodopseudomonas palustris HaA2
Rhodopseudomonas palustris BisB5
Rhodospirillum rubrum ATCC 11170
Hoeflea phototrophica DFL-43 **
Halorhodospira halophila SL1
Congregibacter litoralis KT 71
Marine Gamma Proteobacterium HTCC2080
Rubrivivax gelatinosus S1 **
Spirilloxanthin
*+Canthaxanthin
**+2,2'-Diketospirilloxanthin/
Spheroidenone
Jannaschia sp. CCS1
Roseobacter sp. AzwK-3b
Roseovarius sp. 217
Roseovarius sp. TM1035
Rhodobacter capsulatus
Dinoroseobacter shibae DFL 12
Roseobacter sp. CCS2
Loktanella vestfoldensis SKA53
Roseobacter denitrificans OCh 114
Roseobacter litoralis Och 149
Rhodobacter sphaeroides ATCC 17025
Rhodobacter sphaeroides NCIB 8253
Spheroidene/
Spheroidenone
Marinobacter sp. ELB17 R
Uncultured Marine Bacterium HF10_49E08 R
Alpha Proteobacterium HTCC2255 R
Vibrio campbellii AND4 R
Vibrio harveyi ATCC BAA-1116 R
Alpha Proteobacterium BAL199 R
Photobacterium sp. SKA34 R
Vibrio angustum S14 R
Methylophilales Bacterium HTCC2181 R
Uncultured Marine Bacterium EB0_41B09 R
Uncultured Marine Bacterium HF10_19P19 R
Uncultured Marine Bacterium HF10_25F10 R
Uncultured Marine Bacterium EB0_39F01 R
Alpha Proteobacterium HTCC2255 R
Marine Gamma Proteobacterium HTCC2207 R
Marine Gamma Proteobacterium HTCC2143 R
"Candidatus Pelagibacter ubique" HTCC1002 R
"Candidatus Pelagibacter ubique" HTCC1062 R
β-Carotene
Uncultured Marine Bacterium HF10_29C11 R
Bdellovibrio bacteriovorus HD100 Xanthophylls
Parvularcula bermudensis HTCC2503
Bradyrhizobium sp. ORS278 ***
Paracoccus zeaxanthinifaciens R1534
Paracoccus sp. N81106
Paracoccus haeundaensis
Astaxanthin/
Canthaxanthin/
Zeaxanthin
***+Spirilloxanthin
Sphingopyxis alaskensis RB2256
Sphingomonas sp. SKA58
Novosphingobium aromaticivorans DSM 12444
Erythrobacter sp. SD-21
Erythrobacter litoralis HTCC2594
Erythrobacter sp. NAP1 ***
Erythrobacter longus OCh101 ***
Erythroxanthin/
Nostoxanthin
***+Spirilloxanthin
Caulobacter sp. K31
Brevundimonas vesicularis DC263
Brevundimonas sp. SD212
Nostoxanthin
Xanthobacter autotrophicus Py2
Fulvimarina pelagi HTCC2506 *** R
Aurantimonas sp. SI85-9A1
Pseudomonas stutzeri A1501
Enterobacteriaceae Bacterium DC416
Enterobacteriaceae Bacterium DC260
Enterobacteriaceae Bacterium DC404
Photorhabdus luminescens subsp. laumondii TTO1 R
Cronobacter sakazakii ATCC BAA-894
Enterobacteriaceae Bacterium DC413
Pantoea ananatis ATCC 19321
Pantoea agglomerans Eho10
Astaxanthin/
Canthaxanthin/
Zeaxanthin
***+Spirilloxanthin
Thermus thermophilus HB8
Thermus thermophilus HB27
Zeaxanthin
Deinococcus radiodurans R1
Deinococcus geothermalis DSM 11300
Roseiflexus castenholzii DSM 13941
Roseiflexus sp. RS-1 R
Gloeobacter violaceus PCC 7421 R
Monocyclic xanthophylls
β-Carotene + Echinenone +
Monocyclic xanthophylls
Taxa:
Actinobacteria
Archaea
Bacteroidetes
Chloroflexi
Cyanobacteria
Fungi
Planctomycetes
α-Proteobacteria
β-Proteobacteria
γ-Proteobacteria
δ-Proteobacteria
Thermus/Deinococcus
Unclassified
0.1
