# Supplementary material for: Phylogenetic and Evolutionary Patterns in Microbial Carotenoid Biosynthesis Are Revealed by Comparative Genomics
Source: PLoS One. 2010 Jun 22;5(6):e11257. doi: 10.1371/journal.pone.0011257 (PMC2889829; doi:10.1371/journal.pone.0011257)
Supplement: Supplementary file 8 [file pone.0011257.s017.doc]

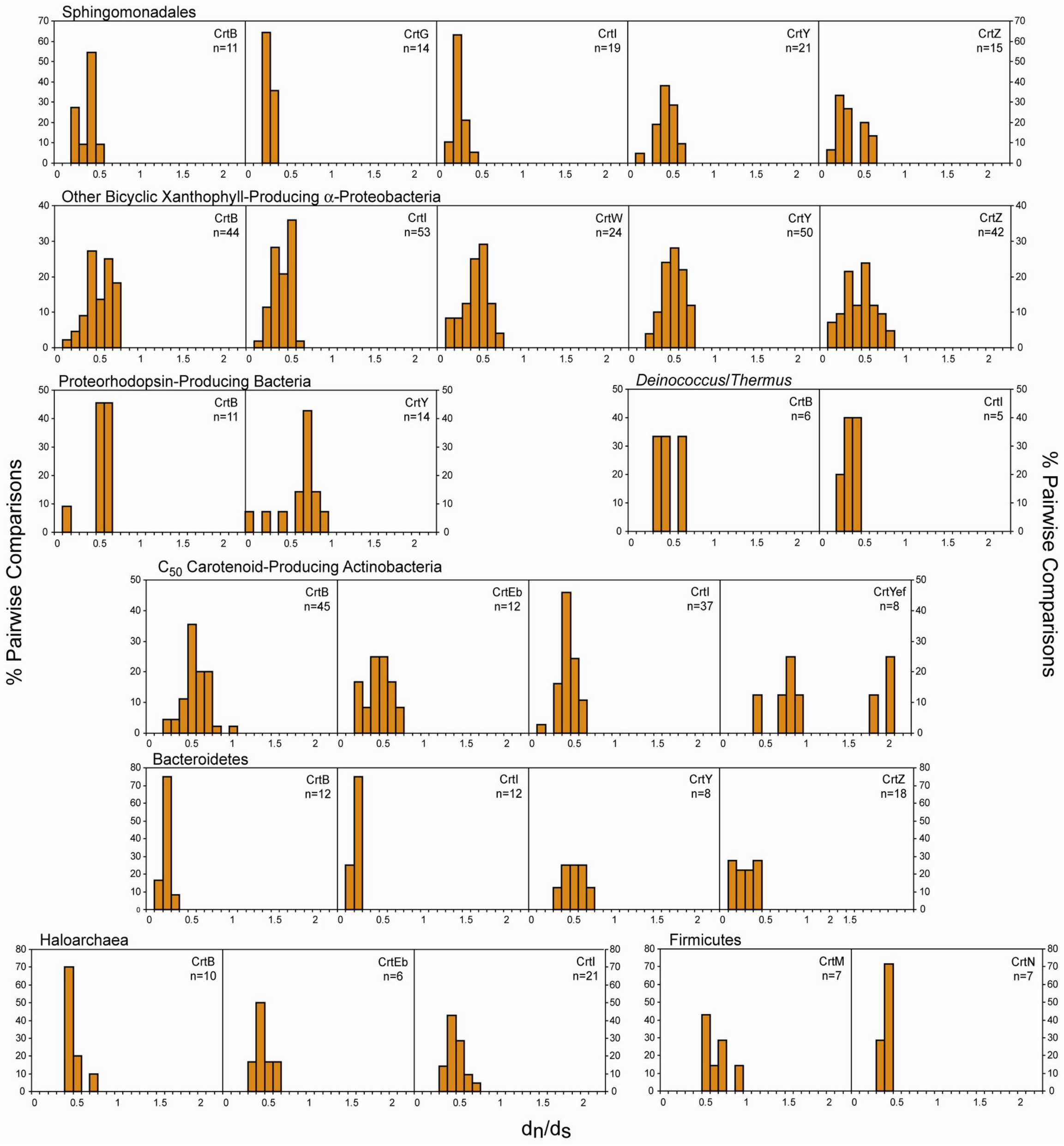

Sphingomonadales
CrtB n=11
CrtG n=14
CrtI n=19
CrtY n=21
CrtZ n=15
Other Bicyclic Xanthophyll-Producing α-Proteobacteria
CrtB n=44
CrtI n=53
CrtW n=24
CrtY n=50
CrtZ n=42
Proteorhodopsin-Producing Bacteria
CrtB n=11
CrtY n=14
Deinococcus/Thermus
CrtB n=6
CrtI n=5
C50 Carotenoid-Producing Actinobacteria
CrtB n=45
CrtEb n=12
CrtI n=37
CrtYef n=8
Bacteroidetes
CrtB n=12
CrtI n=12
CrtY n=8
CrtZ n=18
Haloarchaea
CrtB n=10
CrtEb n=6
CrtI n=21
Firmicutes
CrtM n=7
CrtN n=7
% Pairwise Comparisons
dN/dS

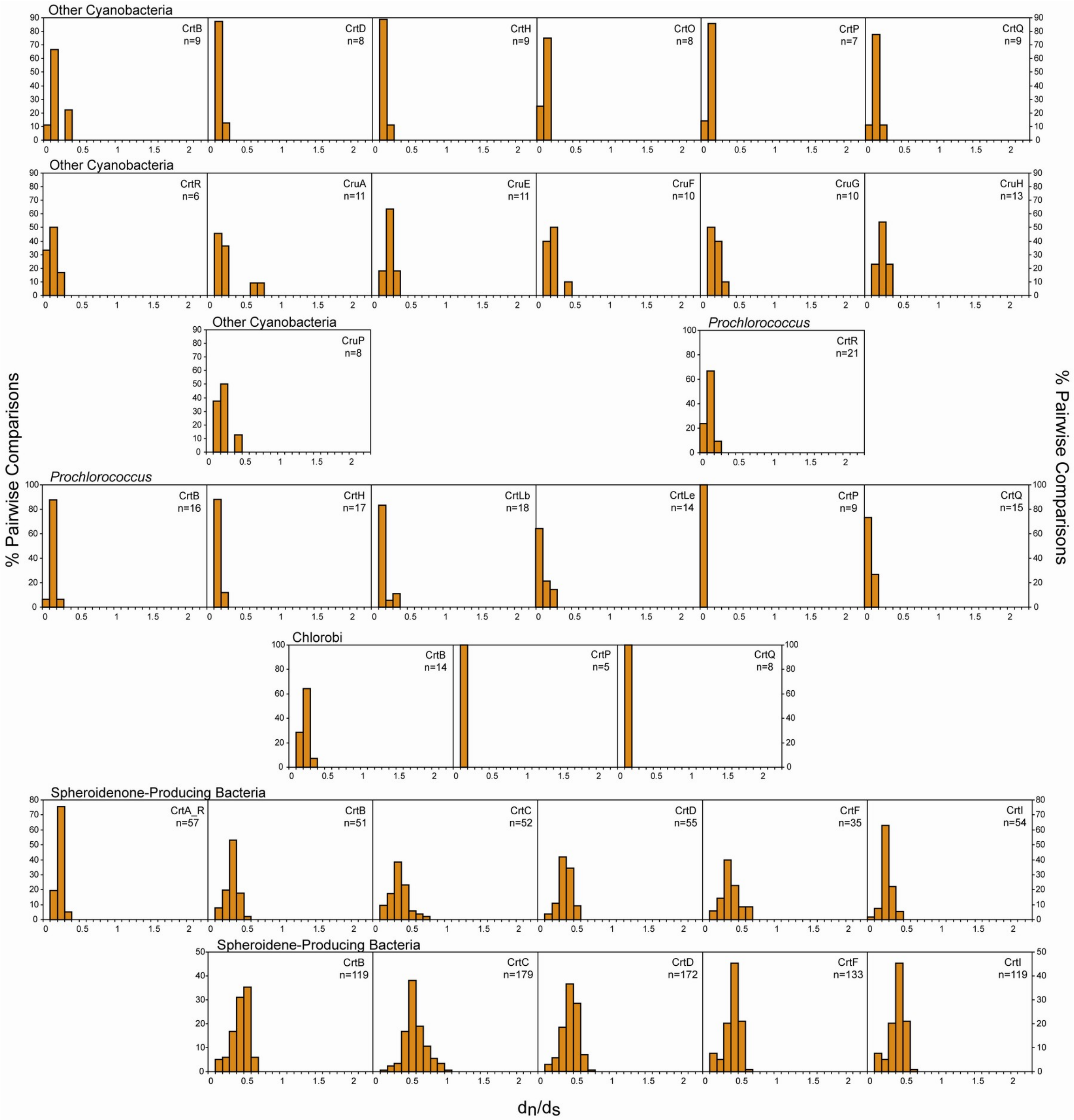
Other Cyanobacteria
CrtB n=9
CrtD n=8
CrtH n=9
CrtO n=8
CrtP n=7
CrtQ n=9
Other Cyanobacteria
CrtR n=6
CruA n=11
CruE n=11
CruF n=10
CruG n=10
CruH n=13
Other Cyanobacteria
CruP n=8
Prochlorococcus
CrtR n=21
Prochlorococcus
CrtB n=16
CrtH n=17
CrtLb n=18
CrtLe n=14
CrtP n=9
CrtQ n=15
Chlorobi
CrtB n=14
CrtP n=5
CrtQ n=8
Spheroidenone-Producing Bacteria
CrtA_R n=57
CrtB n=51
CrtC n=52
CrtD n=55
CrtF n=35
CrtI n=54
Spheroidene-Producing Bacteria
CrtB n=119
CrtC n=179
CrtD n=172
CrtF n=133
CrtI n=119
% Pairwise Comparisons
% Pairwise Comparisons
dn/ds
